# RegionScan: a comprehensive R package for region-level genome-wide association testing with integration and visualization of multiple-variant and single-variant hypothesis testing

**DOI:** 10.1093/bioadv/vbaf052

**Published:** 2025-03-13

**Authors:** Myriam Brossard, Delnaz Roshandel, Kexin Luo, Fatemeh Yavartanoo, Andrew D Paterson, Yun J Yoo, Shelley B Bull

**Affiliations:** Lunenfeld-Tanenbaum Research Institute, Sinai Health, Toronto, ON M5T 3L9, Canada; Genetics and Genome Biology Program, The Hospital for Sick Children, Toronto, ON M5G 0A4, Canada; Lunenfeld-Tanenbaum Research Institute, Sinai Health, Toronto, ON M5T 3L9, Canada; Department of Mathematics Education, Seoul National University, Seoul 08826, South Korea; Genetics and Genome Biology Program, The Hospital for Sick Children, Toronto, ON M5G 0A4, Canada; Dalla Lana School of Public Health, University of Toronto, Toronto, ON M5T 3M7, Canada; Department of Mathematics Education, Seoul National University, Seoul 08826, South Korea; Lunenfeld-Tanenbaum Research Institute, Sinai Health, Toronto, ON M5T 3L9, Canada; Dalla Lana School of Public Health, University of Toronto, Toronto, ON M5T 3M7, Canada

## Abstract

**Summary:**

RegionScan is designed for scalable genome-wide association testing of both multiple-variant and single-variant region-level statistics, with visualization of the results. For detection of association under various regional architectures, it implements three classes of state-of-the-art region-level tests, including multiple-variant linear/logistic regression (with and without dimension reduction), a variance-component score test, and region-level min*P* tests. RegionScan also supports the analysis of multi-allelic variants and unbalanced binary phenotypes and is compatible with widely used variant call format (VCF) files for both genotyped and imputed variants. Association testing leverages linkage disequilibrium (LD) structure in pre-defined regions, for example, LD-adaptive regions obtained by genomic partitioning, and accommodates parallel processing to improve computational and memory efficiency. Detailed outputs (with allele frequencies, variant-LD bin assignment, single/joint variant effect estimates and region-level results) and utility functions are provided to assist comparison, visualization, and interpretation of results. Thus, RegionScan analysis offers valuable insights into region-level genetic architecture, which supports a wide range of potential applications.

**Availability and implementation:**

RegionScan is freely available for download on GitHub (https://github.com/brossardMyriam/RegionScan).

## 1 Introduction

Complex trait genetic architecture is characterized by polygenicity, where multiple genetic variants, each contributing a small effect, collectively influence the trait and possibly interact with other variants and/or environmental factors. The effects of these variants may vary across different populations or environments, complicating the understanding and prediction of complex traits. Genetic architecture also varies locally across regions of the genome; these regional differences arise from selective pressures and unique population histories (Slatkin 2008). Local genetic architecture is characterized by variations in the number of causal variants (Hormozdiari *et al.* 2017), patterns of linkage disequilibrium (LD), genetic heritability (Smith *et al.* 2022), and regional polygenicity (Johnson *et al.* 2021).

The standard approach for genome-wide association study (GWAS) is based on single-variant testing. This approach is generally effective at detecting variants with larger effect sizes and higher allele frequencies that meet the stringent GWAS significance criterion, particularly in studies with large sample sizes (such as biobanks). The single-variant GWAS approach has led to the identification of numerous genome-wide significant associations for many complex traits. However, challenges remain, including unexplained missing genetic heritability for many traits and uncertainty surrounding functional variants at discovered loci. Application of fine-mapping approaches in each of the GWAS loci to prioritize candidate sets of potentially functional variants requires specification of locus boundaries (e.g. often an arbitrary region around detected variants), and application of conditional analysis approaches or more sophisticated statistical fine-mapping methods in each region (e.g. Wang *et al.* 2020, Benner *et al.* 2025). Furthermore, for traits with low prevalence (e.g. some cancers) or rare diseases, case numbers are still limited; this applies to underrepresented ancestry groups as well, where large datasets are still lacking. In such cases, the statistical power of single-variant analysis remains limited.

In contrast, region-level testing offers a complementary approach to single-variant analysis which can: (i) enhance power to uncover regions missed by single-variant analysis (e.g. that harbor multiple independent variants with weaker effects, or in studies with limited sample size, by aggregating multiple variant effects while accounting for local LD via multiple regression approaches); (ii) inform fine-mapping efforts by offering insights into the regional genetic complexity; (iii) facilitate cross-ancestry investigations, where allele frequencies and effects within regions may differ across populations; (iv) combine results from region-level tests based on rare variants with those from common variants, providing a more comprehensive understanding of the regional genetic landscape (Neale and Sham 2004, Gauderman *et al.* 2007, Yoo *et al.* 2015, 2017, Hormozdiari *et al.* 2017).

Region-level approaches nevertheless face challenges in defining regions for a comprehensive genome-wide analysis (including intergenic variants) and addressing the analytical complexities associated with high-dimensional data and multicollinearity within regions. The increasing variant density enabled by current and emerging sequencing technologies adds to these challenges. Existing region-level tests differ in underlying assumptions and test statistic construction, making them differentially sensitive to variation in regional architectures. The power of these tests is influenced by factors including allele frequencies, LD structure, marginal effect sizes of causal and non-causal variants, and the ratio of causal to non-causal variants within a region. Because no one region-level test consistently outperforms other tests across all scenarios of regional complexity, various multiple-variant tests have been developed to improve discovery. Regression-based tests are robust across a range of causal variant complexities but benefit from dimension reduction using region-level principal components (e.g. PC80, Gauderman *et al.* 2007), especially when multicollinearity is extreme. SKAT-type variance-component score tests are computationally efficient and applicable for both rare and common variant analysis (e.g. Wu *et al.* 2011, Lee *et al.* 2012), although simulation studies have reported reduced power for SKAT compared to other tests, depending on local genetic architecture. For example, SKAT has lower power in comparison to single-variant tests in simulated regions with multiple common causal variants (Uemoto *et al.* 2013) and to other region-level tests in regions with a high proportion of non-causal variants (Derkach *et al.* 2014, Yoo *et al.* 2017). Min*P-type* tests based on marginal single-variant analysis are more powerful when there is a single causal variant with a large effect, but local LD needs to be considered for Type I error control. Combining the results of multiple tests within each region, using, for example, the Cauchy combination test (Liu *et al.* 2019), can enhance region discovery across various regional architectures, while reducing false negatives and accounting for correlation among test statistics.

We introduce the RegionScan R package designed for comprehensive genome-wide region-level analysis and compatibility with regions defined by blocks of high LD obtained through genomic LD-partitioning implemented in BigLD/gpart software (Kim *et al.* 2018, 2019). This approach reduces the level of multiple testing while examining the whole genome, in contrast to gene-based definitions which exclude intergenic regions.

## 2 Overview and key features

In what follows, we describe the implementation of RegionScan and illustrate its capabilities through application in a complex quantitative trait GWAS. Methodological details for the region-level tests implemented are specified in Supplementary Information S1, while the steps involved in variant processing and generating outputs are detailed in Supplementary Information S2. Supplementary Information S3 details the GWAS application and results. Supplementary Information S4 reports computational time evaluation, while Supplementary Information S5 comments on potential research applications.

### 2.1 Implementation

The main function, *regscan* (Table 1, and Supplementary Information S2) takes four main user-provided input files: “data” which includes genotypes; “SNPinfo” with variant information; “phenocov” with phenotypes as well as potential covariates; and “REGIONinfo” with region positions as produced by the BigLD algorithm in gpart R package (Kim *et al.* 2018, 2019) or any other user-specified regions input file including gene or window start/end positions. Unlike window-based definitions, LD-adaptive regions are characterized by high within-region and low between-region correlation and quasi-independence. User-specified definitions such as fixed-window or gene-based regions that do not share the quasi-independence property can be subject to high between-region correlation among test statistics. Furthermore, because region-level analysis reduces multiple testing and LD-adaptive regions are quasi-independent, it permits a less stringent genome-wide significance threshold based on a Bonferroni correction for the number of regions, without sacrificing Type I error control. *regscan* can also generate “data” and “SNPinfo” input files by region from large VCF4.0 files to improve memory efficiency using the function *recodeVCF* (Table 1).

**Table 1.** Main and utility functions in the RegionScan package.

Functions	Short description
*regscan*	Main function for region processing, single-variant and region-level analysis (details in Supplementary Information S2).
*recodeVCF*	Auxiliary function to extract information from VCF file for each region, recode variants (including multiallelic variants) and return “data” and “SNPinfo” input files used by *regscan*.
*MiamiPlot*	Produces a Miami plot genome-wide (or in a subset of regions) for any specified pair of tests, e.g. Fig. 1A. Can also be used to visualize potential colocalization between region-level tests obtained for two independent studies.
*qqregscanPlot*	Creates a Quantile-Quantile plot for a specified region-level test and calculates genomic inflation factor, e.g. Supplementary Fig. S5.
*LocusPlot*	Plots region-level test results for a specified set of contiguous regions, e.g. Fig. 1C.
*MLCbinsnpPlot*	Visualization of genomic positions of the variants assigned to within-region LD bins, e.g. right panel in Fig. 1D.

The processing steps for each region include variant filtering and recoding based on minor allele frequency and options to (i) process multi-allelic variants in addition to bi-allelic variants and (ii) reduce multicollinearity by pruning variants based on within-region LD. In addition to conventional single-variant tests, *regscan* applies multiple-variant tests from three classes of state-of-the-art region-level tests (see Supplementary Information S1), including multiple-variant linear/logistic regression tests with and without dimension reduction (Li and Lagakos 2006, Gauderman *et al.* 2007, Yoo *et al.* 2017), a variance-component score test (Kwee *et al.* 2008, Wu *et al.* 2011, Lee *et al.* 2012), and region-level min*P* tests [e.g. simpleM (Gao *et al.* 2008), GATES (Li *et al.* 2011)]; these approaches exhibit differential sensitivity for regional detection, as demonstrated in simulation studies (Yoo *et al.* 2017). To obtain the reduced-*df* MLC region-level test (Yoo *et al.* 2015, 2017), variants within each region are assigned to LD bins using clique-based clustering of correlated variants based on pairwise correlation (Yoo *et al.* 2015). Within each bin, variants are recoded to maximize positive correlation among variant pairs, followed by construction of multiple linear combination contrasts corresponding to the LD bins (Supplementary Information S1); bin-level tests within a region are computed in addition to MLC region-level tests. The 1-*df* region-level LC test is a burden-type test obtained by assigning all variants in a region to one bin and recoding variants to maximize positive correlation. *regscan* also includes an option to reduce finite-sample bias in logistic regression of unbalanced binary traits and/or variants with low minor allele counts, using a Jeffreys-prior penalized likelihood (Kosmidis *et al.* 2020, Kosmidis and Firth 2021).

### 2.2 Outputs and visualization

*regscan* produces four output files detailing results for each region (Supplementary Information S2): (i) “region-level” file with multiple region-level test results for all regions; (ii) “bin-level” file including MLC bin-level test results for all LD bins within each region; (iii) “variant-level” file with variant positions, LD-bin assignments, and corresponding effect sizes and *P* values from multiple-variant region-level and single-variant regression models; within-region variance inflation factor values (VIFs) are calculated to identify sources of multi-collinearity; (iv) a list of variants pruned out with reasons for exclusions. Two optional output files are reported: one with single-variant test results for all the variants (available before pruning) and another with covariate effect estimates and *P* values extracted from single- and/or multiple-variant regression models. Utility functions are also implemented to assist with comparison, visualization, and interpretation of the results (Table 1).

### 2.3 Illustration

**Figure 1.**
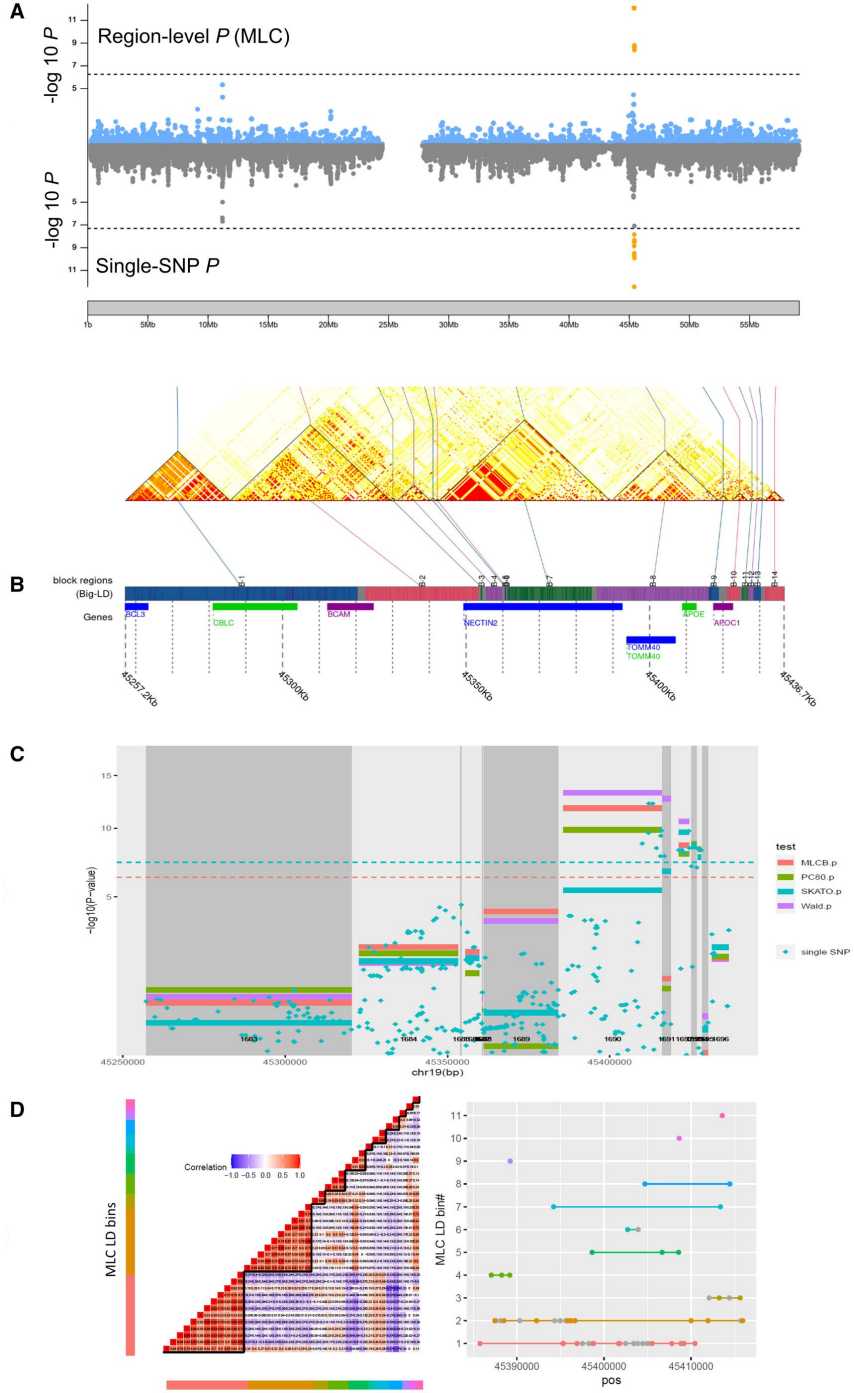
RegionScan results for genome-wide region-level association analysis of LDL-C at baseline in 1340 individuals from the DCCT/EDIC study. (A) Miami plot of chromosome 19 that compares region-level MLC test (top panel) with single-variant results (bottom panel) in 2548 regions tested; (B) LD heatmap for 14 consecutive regions with boundary delimiters and annotated with gene positions produced using the *LDblockHeatmap* function in BigLD/gpart; (C) Region-level test results for the regions shown in (B); changes in grey shading highlight the region boundaries. Dashed horizontal lines indicate GW significance thresholds: 5E−8 for single-SNP analysis and the genome-wide region-level significance threshold of 5.62E−7 for region-level tests. Region numbers on chromosome 19 appear at the bottom of the plots. (D) Visualization of the LD bins in region #1690 that includes *APOE* (labelled “B8” on panel B); the left panel displays pairwise variant correlation with variants grouped by LD bin (shown by distinct color bands); the right panel displays variant positions (*X* axis) according to the LD-bin assignment (*Y* axis) for the variants kept after LD pruning (variants pruned out are shown in grey). Details of the analysis and results are available in Supplementary Information S3.

**Figure 2.**
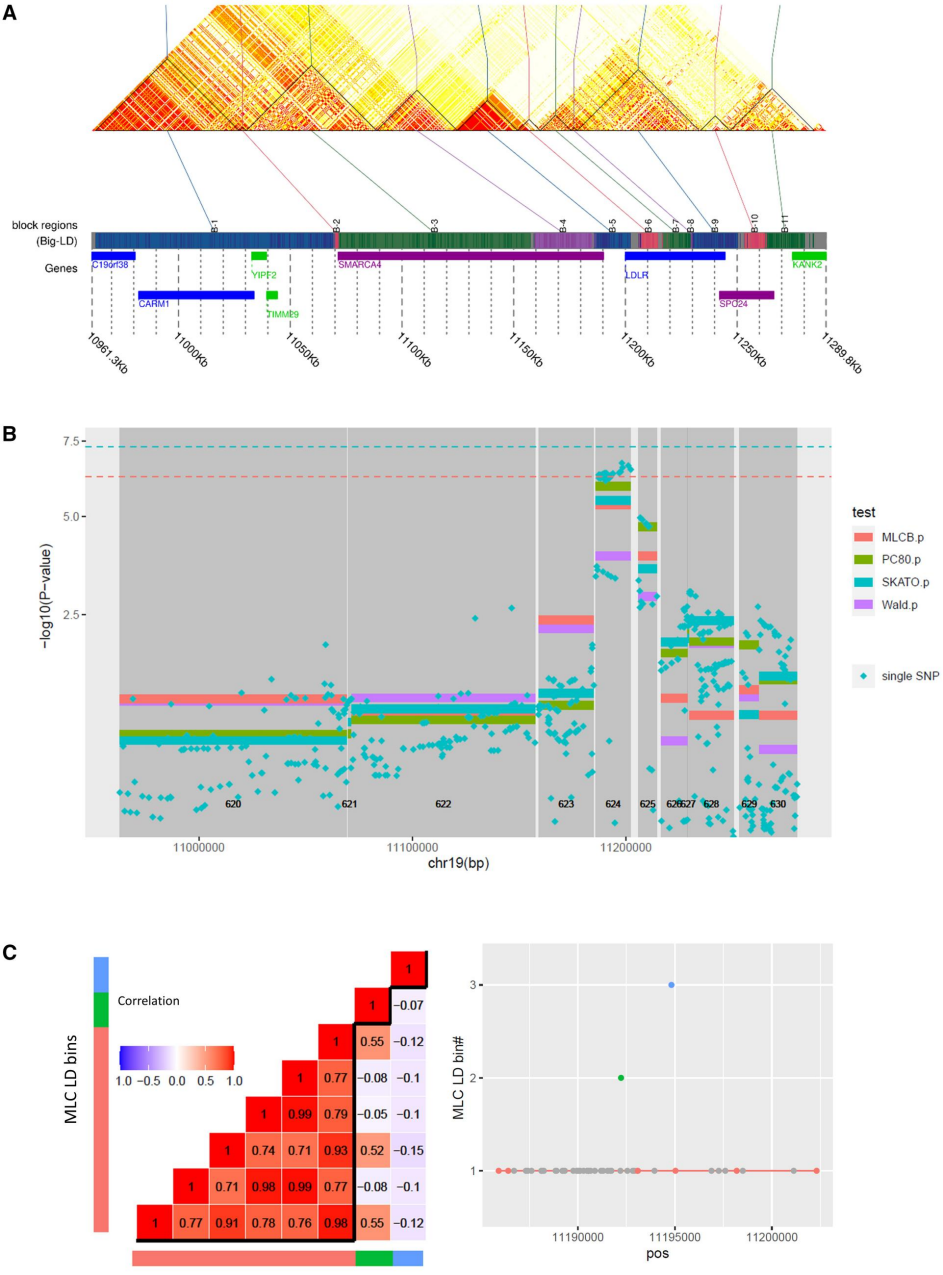
RegionScan results in the well-established *LDLR* region for LDL-C in chromosome 19. Panel (A) shows LD heatmap for 11 consecutive regions with boundary delimiters and annotated with gene positions produced using the *LDblockHeatmap* function in BigLD/gpart; (B) Region-level test results for the regions shown in (A); changes in grey shading highlight the region boundaries. Dashed horizontal lines indicate GW significance thresholds: 5E−8 for single-SNP analysisand the genome-wide region-level significance threshold of 5.62E−7 for region-level tests. Region numbers on chromosome 19 appear at the bottom of the plots. (C) Visualization of the LD bins in region #624 (partially overlapping *LDLR* and including well established variant rs6511720 for LDL-C; labelled “B5” on panel A); the left panel displays pairwise variant correlation with variants grouped by LD bin (shown by distinct color bands); for the 8 SNPs kept after LD pruning, with SNPs ordered by LD bin; rs6511720 is part of the largest LD bin. Details of the analysis and results are available in Supplementary Information S3.

We applied *regscan* in a GWAS of low-density lipoprotein-cholesterol (LDL-C) at baseline in the DCCT/EDIC Genetics study (DCCT Research Group 1986, Paterson *et al.* 2010) analyzing 89 001 autosomal regions obtained by BigLD/gpart. We identified a total of five regions that exhibit significant region-level test results for at least one test at the Bonferroni-corrected threshold of 5.62E−7. These five regions are all located in chr19: 45 385 759–45 428 234 bp, encompassing the well-known *APOE* locus associated with LDL-C. Figure 1 gives an overview of the results and plots produced by utility functions described in Table 1. Figure 1A compares results for region-level MLC test (top panel) with single-variant results (bottom panel) in 2548 regions tested in chromosome 19; both MLC region-level and single-SNP tests detect genome-wide significant associations in the same locus. Figure 1B shows the LD heatmap for 14 consecutive regions around *APOE* with boundary delimiters and annotated with gene positions. Region-level and single-variant results for the same regions are shown in Fig. 1C (changes in grey shading highlight the region boundaries). In Table 2, we summarize results from region #1690 (Fig. 1C and D) and region #624 (Fig. 2), which overlap two well-established genes for LDL-C (*APOE* for region #1690 and *LDLR* for region #624) in chromosome 19. These regions exhibit contrasting regional complexity features and region-level tests with differing strength of association (Table 2).

**Table 2.** Insights from RegionScan in two well-established genetic association regions in chromosome 19 for LDL-C with different regional complexities.^a^

Region	Regional complexity	Summary of insights from RegionScan
#624 (*LDLR*)	16 kb-long, 51 SNPs (8 SNPs kept after pruning on LD) assigned to three LD bins; High LD focused within the region (Fig. 2A–C); LD structure differs from region #1690 (Fig. 1B–D); Potentially one causal variant; Partially overlaps *LDLR*.	Suggestive association for MLC, Wald, SKAT-O and Uncorrected top single-SNP tests (Fig. 2B). Improved *P* values for reduced-*df* MLC and PC80 compared to full multiple regression Wald test (Fig. 2B). Within the region, MLC bin-level tests detect an association for the largest bin (Fig. 2C), which includes the top single variant associated with LDL-C in previous large GWAS (rs6511720).
#1690 (*APOE*)	30 kb-long, almost twice as long as region #624; 61 SNPs (38 SNPs kept after pruning on LD) assigned to 11 LD bins, suggesting a more complex LD structure than region #624; Extended LD pattern with two independent well-established *APOE* causal SNPs (rs429358 and rs7412); Fully overlaps with *APOE*.	Wald, MLC, PC80, and single-SNP tests all reach genome-wide significance thresholds, SKAT-O does not (Fig. 1C). Stronger *P* values for Wald test compared to MLC, PC80 and SKAT-O (Fig. 1C) Two well-established *APOE* variants are assigned to two LD bins (bin 2 and 3, Fig. 1D); bin-level association is detected in LD bin 3, including the *APOE* variant rs7412; the bin-level association in LD bin 2 for the other *APOE* variant (rs429358) is weaker due to complex LD & haplotype effects in this region. Haplotype heterogeneity resulting from joint effects of two independent SNPs with complex minor allele effects.

a Detailed results for these two regions are shown in Supplementary Information S3.

### 2.4 Computation

We assessed the elapsed and CPU times of the *regscan* function across regions of varying complexity, defined by the number of variants analyzed and LD bins within each region. This was evaluated in a binary trait analysis with a balanced case/control ratio (for details, see Supplementary Information S4) performed on a high-performance computer platform (HPC) using a single node with 40 CPUs and 202 GB RAM. The *regscan* function was executed for each region using default settings, which included default options for variant pre-processing (pruning, LD bin construction), calculation of eight region-level tests, and single-variant analysis. As anticipated, our results show a clear increase in both elapsed and CPU times with larger sample sizes and greater region complexities. Specifically, for a region containing 100 SNPs, *regscan* takes 10.3 s of elapsed time (3.02 min of CPU time) when analyzing 10 000 individuals and 33.36 s of elapsed time (12.92 min of CPU time) when analyzing 40 000 individuals. In a larger region with greater complexity, including 594 SNPs, *regscan* takes 2.29 min of elapsed time (28.42 min of CPU time) for 10 000 samples, and 4.93 min of elapsed time (86.40 min of CPU time) for 40 000 individuals. Given that most regions analyzed contain fewer than 100 SNPs (Supplementary Figs S3 and S12) and considering that binary traits generally require longer computational times than quantitative traits due to slower convergence of fitting algorithms, these evaluations highlight the feasibility and scalability of *regscan* for genome-wide analyses.

## 3 Conclusion

RegionScan is a flexible and versatile R package designed for scalable and comprehensive genome-wide region-level analysis that leverages region definition derived from chromosome-level LD structure (or other user-specified region definitions). It implements multiple region-level tests sensitive to heterogeneous genetic architecture, including reduced-*df* region-level tests constructed using within-region LD structure, which facilitates comparisons among region-level and single-variant test results.

A major advantage of RegionScan over other software that apply only a single testing method is the additional information obtained about the region-level LD bin structure, the implementation of regression-based tests including the MLC region-level test which is not available in any other software, and capacity to compare the results of alternative test statistics at the genome-wide level. Using a single run of the main function (*regscan*), a user obtains several valuable pieces of information to facilitate characterization of genomic resolution, including LD bins within regions and LD bin-level tests. Functions to visualize and compare signals across region-level tests and neighboring regions are also implemented.

In summary, RegionScan can be used to facilitate region discovery for complex trait association, investigate associations within regions, and provide insights into regional complexities relevant for subsequent statistical fine-mapping investigations. Information obtained for regions detected by RegionScan can also be used to guide region-level heritability estimation (Smith *et al.* 2022), regional polygenicity characterization (Johnson *et al.* 2021), and local genetic correlation estimation (Shi *et al.* 2017) as well as inform region-level colocalization analysis between traits using colocalization approaches [e.g. SharePro (Zhang *et al.* 2024)] and integration of common and rare variant analyses (discussed in Supplementary Information S5). The modular design of RegionScan permits flexibility for the development of future extensions, including mapping of region- and LD bin-level association signals.

## Supplementary Material

vbaf052_Supplementary_Data

## Data Availability

The R package RegionScan is available on GitHub (https://github.com/brossardMyriam/RegionScan) and includes a vignette on how to install and run RegionScan in a realistic artificial dataset provided. The DCCT/EDIC data are available to authorized users at https://repository.niddk.nih.gov/studies/edic/ and https://www.ncbi.nlm.nih.gov/projects/gap/cgi-bin/study.cgi?study_id=phs000086.v3.p1. Data analysis, software development, and computation time estimation were performed using the Hospital for Sick Children High-performance Computing Facility, the Lunenfeld-Tanenbaum Research Institute High-performance Computing platform, and/or the Niagara supercomputer (with support from the Canada Foundation for Innovation under the auspices of Compute Canada; the Government of Ontario, Ontario Research Fund—Research Excellence and the University of Toronto). For Niagara hardware specifications, see https://docs.computecanada.ca/wiki/Niagara#Niagara_hardware_specifications and https://docs.scinet.utoronto.ca/index.php/Niagara_Quickstart.
