# Supplementary material for: RegionScan: a comprehensive R package for region-level genome-wide association testing with integration and visualization of multiple-variant and single-variant hypothesis testing
Source: Bioinform Adv. 2025 Mar 13;5(1):vbaf052. doi: 10.1093/bioadv/vbaf052 (PMC11951254; doi:10.1093/bioadv/vbaf052)
Supplement: vbaf052_Supplementary_Data [file vbaf052_supplementary_data.pdf]

Supplementary Information

RegionScan: A comprehensive R package for region-level genome-wide association testing with integration and visualization of multiple-variant and single-variant hypothesis testing

Myriam Brossard[1], Delnaz Roshandel[2], Kexin Luo[1], Fatemeh Yavartanoo[3], Andrew D. Paterson[2,4] Yun J. Yoo[3], Shelley B. Bull[1,4]
[1]Lunenfeld-Tanenbaum Research Institute, Sinai Health, Toronto, Ontario, Canada;
[2]Genetics & Genome Biology Program, The Hospital for Sick Children, Toronto, Ontario, Canada;
[3]Seoul National University, Seoul, South Korea;
[4]Dalla Lana School of Public Health, University of Toronto, Toronto, Ontario, Canada.

Contents

Supplementary Information 1. Region-level tests implemented 2
1.1 Methods 2
1.2 Summary of region-level tests performances previously reported 6
Supplementary Information 2. Details on the main and auxiliary functions 7
2.1 *regscan*: main function to process and analyze regions 7
2.1.1 Steps involved in processing and analysis of each region 7
2.1.2 List of main arguments and default values 12
2.1.3 Description of the main outputs of *regscan* function 14
2.2 *recodeVCF*: auxiliary function to extract and process SNPs in VCF files 17
2.2.1 Processing of the bi-allelic SNPs (multiallelic = FALSE) 17
2.2.2 Processing of multi-allelic SNPs (multiallelic = TRUE) 17
2.2.3 Description of outputs 20
2.3 Vignette and illustration of the main functions in a small example 20
Supplementary Information 3. Application in the DCCT/EDIC genetic study 21
3.1 DCCT/EDIC analysis methods 21
3.2 Results 24
3.3 Two regions exhibiting association signals 28
3.3.1 “APOE locus” (chr19: 45257201- 45436657) 28
3.3.2 “LDLR locus” (chr19: 10962974- 11280183) 38
3.4 List of the participants of the DCCT/EDIC Research Group (as of January 1, 2023) 44
Supplementary Information 4. Computation time evaluations in an artificial dataset 47
4.1 Dataset generation 47
4.2 Assessment of computational efficiency 47
Supplementary Information 5. Comments on potential utility of RegionScan for other applications 52
5.1 Colocalization analysis for two traits 52
5.2 Combination of region-level tests obtained for common and rare variants 52
References 53
Annex. Vignette of RegionScan 55

Supplementary Information 1. Region-level tests implemented

1.1 Methods

In this section, we assume a region with K SNPs (genotyped and/or imputed) in N individuals, and we define:

- $\boldsymbol{G}$, a N-by-K genotypes matrix, where each genotype is coded as the number of copies of the minor allele,
- $\boldsymbol{Y}$, a N-by-1 phenotypic vector (qualitative or binary),
- $\boldsymbol{X}$, a N-by-C matrix of C covariates (if applicable).

We classify the region-level tests implemented in RegionScan into three categories: multi-SNP linear/logistic regression-based tests, variance component score tests, and region-level minimum P-like tests (**Table S1**).

Regression-based tests: For the category of multiple-SNP linear/logistic regression tests, we assume a region-level multi-SNP regression model:

$$g(E[\boldsymbol{Y}]) = \beta_0 + \boldsymbol{G\beta} + \boldsymbol{X\alpha} \quad \text{(Equation 1)}$$

Where:

- $g(.)$ is a link function and can be set to the identity function, if $\boldsymbol{Y}$ is quantitative, or to the logit function if $\boldsymbol{Y}$ is binary,
- $\boldsymbol{\beta} = (\beta_1, \dots, \beta_k, \dots, \beta_K)^T$ is the K-length vector of SNP effects,
- $\boldsymbol{\alpha}$ is a vector of covariate effects (if applicable).

The multi-SNP linear/logistic regression-based tests, Generalized Wald test, LC and MLC tests, described in **Table S1** are all based on the effect estimates vector $\widehat{\boldsymbol{\beta}}$ and variance-covariance matrix $\boldsymbol{\Sigma}$ estimated from the regression model defined in Equation 1.

Single-variant regression minP-like tests: For the region-level min$P$-like tests, we define $\boldsymbol{Z} = (z_1, \dots, z_k, \dots, z_K)$, as the vector of Z scores from single-SNP regression models applied to each of the K SNPs ($\boldsymbol{G_k}, 1 \le k \le K$) in the region, that is:

$$g(E[\boldsymbol{Y}]) = \beta_{0k} + \boldsymbol{G_k}\beta_k + \boldsymbol{X\alpha_k}$$

We define p_k as the P-value from the 1 degree of freedom (*df*) Wald test applied to each SNP effect β_k (H0: $\beta_k = 0$ *vs* H1: $\beta_k \neq 0$), and denote $p_{(1)} \le \cdots \le p_{(k)} \le \cdots \le p_{(K)}$ as the single-SNP P-values for all K SNPs ranked by increasing order.

Variance component score tests: The region-level tests are defined by a statistic $Q = (\boldsymbol{Y} - \widehat{\boldsymbol{\mu_0}})^T \boldsymbol{H} (\boldsymbol{Y} - \widehat{\boldsymbol{\mu_0}})$ where:

- $\widehat{\boldsymbol{\mu_0}}$ corresponds to the predicted phenotype vector obtained by fitting the following null genetic model (with only covariates): $g(E[\boldsymbol{Y}]) = \beta_0 + \boldsymbol{X\alpha}$,
- $\boldsymbol{H} = \boldsymbol{GWWG}^T$ is a N-by-N kernel matrix (positive semidefinite) and $\boldsymbol{W} = diag(w_1, \dots, w_k, \dots, w_K)$ is a diagonal matrix of specified SNP weights.

Table S1. Main characteristics of the region-level tests implemented to capture various types of region-level architectures

**Region-level test**	**Method/Features**	**Test Statistic**	**Default parameter values in *regscan***
**Multi-SNP linear/logistic regression tests[a]**			
Wald<br>(default)	Generalized Wald test<br>(Quadratic test, multi-directional)	$G_{Wald} = \hat{\boldsymbol{\beta}}^T \boldsymbol{\Sigma}^{-1} \hat{\boldsymbol{\beta}}$<br>with $\boldsymbol{\Sigma} = \mathbf{Var}[\hat{\boldsymbol{\beta}}]$<br>H0: β_k =0 **for *all K* SNPs**<br>*vs* H1: ***at least* one** $\beta_k \neq 0$<br>which is a ***multi-directional* alternative**<br>Under H0: $G_{Wald} \sim \chi^2$ with *K df*	None
LC<br>(Pocock et al., 1987; Stram et al., 1988; Li and Lagakos, 2006)<br>(default)	Linear Combination test<br>(Linear test, uni-directional)<br>The *K* SNPs in the region are recoded to maximize the number of SNP pairs with **positive correlation.**	$G_{LC} = (\boldsymbol{C}^T\hat{\boldsymbol{\beta}})^T (\boldsymbol{C}^T \boldsymbol{\Sigma}\, \boldsymbol{C})^{-1} (\boldsymbol{C}^T\hat{\boldsymbol{\beta}})$<br>Where:<br>• $\boldsymbol{C}$: (*K*-by-1) linear contrast vector, with $\boldsymbol{C} = (\boldsymbol{\Sigma}^{-1}\boldsymbol{J})(\boldsymbol{J}^T\boldsymbol{\Sigma}^{-1}\boldsymbol{J})^{-1}$ → $\boldsymbol{C}^T = (c_1, \ldots, c_k, \ldots c_K)$<br>• $\boldsymbol{J}$: is the (*K*-by-1) vector of 1*'s* that assigns all *K* SNPs to one single bin.<br>For $\delta = \boldsymbol{C}^T\boldsymbol{\beta}$, H0: $\delta = 0$ *vs* H1: $\delta \neq 0$<br>which is a ***uni-directional* alternative**<br>Under H0: $G_{LC} \sim \chi^2$ with 1 *df*	None<br>If *alltests*=TRUE, additional equivalent LC test but based on *Z*-scores (noted LCZ) is provided in output (see (Yoo *et al.*, 2017) for mathematical details)
MLC<br>(Yoo *et al.*, 2017)<br>(default)	Multiple Linear Combinations test, Reduced-*df* test adaptive to LD within region<br>(Hybrid linear – quadratic combination test)<br>The *K* SNPs are clustered into *L* LD bins of correlated SNPs using the CLQ algorithm(Yoo *et al.*, 2015); within each LD bin, the SNPs are **recoded** to ***maximize*** the number of SNP pairs with ***positive*** **correlation.**	$G_{MLC} = (\boldsymbol{W}^T\hat{\boldsymbol{\beta}})^T (\boldsymbol{W}^T \boldsymbol{\Sigma}\, \boldsymbol{W})^{-1} (\boldsymbol{W}^T\hat{\boldsymbol{\beta}})$<br>Where:<br>• $\boldsymbol{\beta}^T = (\boldsymbol{\beta}_1^T \mid \ldots \mid \boldsymbol{\beta}_l^T \mid \ldots \mid \boldsymbol{\beta}_L^T)$ **&** $\boldsymbol{\Sigma}$, are ordered by LD bin, such that each $\boldsymbol{\beta}_l = (\beta_{k_{l,1}}, \ldots, \ldots, \ldots, \beta_{K_l})$ corresponds to the vector of SNP effects for all K_l SNPs assigned to the lth LD bin;<br>• $\boldsymbol{W} = (\boldsymbol{\Sigma}^{-1}\boldsymbol{J})(\boldsymbol{J}^T\boldsymbol{\Sigma}^{-1}\boldsymbol{J})^{-1}$ is a (*K*-by-*L*) matrix of linear contrasts;<br>• $\boldsymbol{J} = [\, \boldsymbol{J}_1 \mid \boldsymbol{J}_2 \mid \ldots \mid \boldsymbol{J}_L \,]$ is the (*K*-by-*L*) indicator *matrix* that assigns *K* SNPs to *L* LD bins $\boldsymbol{W}^T = [\, \boldsymbol{W}_1 \mid \ldots \mid \boldsymbol{W}_l \mid \ldots \mid \boldsymbol{W}_L \,]^T$, where $\boldsymbol{W}_l^T = [\, \boldsymbol{W}_{l1}^T \mid \boldsymbol{W}_{l2}^T \mid \ldots \mid \boldsymbol{W}_{ll}^T \mid \ldots \mid \boldsymbol{W}_{lL}^T \,]$ is (1-by-*K*) vector of linear contrasts for each LD bin *l;*<br>• $\boldsymbol{W}_{ll}$ and $\boldsymbol{W}_{lm}$: LD matrices *within* and *between* LD bins.	Clustering parameter:<br>*edgecut*=0.50<br>(minimum absolute correlation between SNPs in a LD bin, note: corresponds to r2 =0.25)<br>If *alltests*=TRUE, additional equivalent MLC test but based on *Z*-scores (noted MLCZ) is provided in output (Yoo *et al.*, 2017) for mathematical details).

	Note: MLC includes as special cases, the Generalized Wald test (when L=K) and the LC test (when L=1).	For $\boldsymbol{\delta} = \boldsymbol{W^T\beta}$, where each $\delta_l = \boldsymbol{W_l}^T\boldsymbol{\beta}$ corresponds to the bin-level effect for l^{th} bin.<br><br>H0: $\delta_l = 0$ **for all *L* LD bins** *vs* H1: **at least *one*** $\delta_l \neq 0$, which is a ***reduced-dimension multi-directional alternative*** (sensitive to directional effects defined by each LD bin)<br><br>$\boldsymbol{G_{MLC}} \sim \boldsymbol{\chi^2}$ with $\boldsymbol{L \leq K}$ ***df***	
PC80<br>(Gauderman *et al.*, 2007)<br><br>(default)	**Dimension reduction step:** Principal component analysis of the *K* SNPs, selection of the *S* PC-SNPs, that explain at least PCcut=80% of the total SNP variance.<br><br>Multiple regression of the *S* PC-SNPs & Generalized Wald test.<br><br>Reduced-df test ($S \leq K$)<br>(multi-directional)	$G_{PC80} = \hat{\boldsymbol{\gamma}}^T \boldsymbol{\Pi}^{-1} \hat{\boldsymbol{\gamma}}$<br><br>$G_{PC80} \sim \chi^2_S$ with $S \leq K$ *df*<br><br>Where: $\hat{\boldsymbol{\gamma}} = (\widehat{\gamma_1}, \ldots, \widehat{\gamma_s}, \ldots, \widehat{\gamma_S})$ is the vector of PC-SNPs effects and $\boldsymbol{\Pi}$ is the corresponding variance-covariance matrix, estimated by multiple regression.<br><br>H0: $\gamma_s = 0$ **for *all S* PC-SNPs** *vs* H1: **at *least* one** $\gamma_s \neq 0$<br>which is a ***reduced-dimension multi-directional alternative***.	*PCcut*: Variation explained by the PCs selected (default PCcut=80%)
**Variance component score tests**			
SKAT<br>(Kwee *et al.*, 2008)<br><br>(default)	Variance-like component test under a logistic/linear mixed model	**Variance-component score statistic:**<br>$Q_{SKAT} = (\boldsymbol{Y} - \widehat{\boldsymbol{\mu_0}})^T \boldsymbol{H} (\boldsymbol{Y} - \widehat{\boldsymbol{\mu_0}})$<br>Where:<br>• $\widehat{\boldsymbol{\mu_0}}$: predicted phenotypes obtained by fitting the null genetic model (without SNP);<br>• $\boldsymbol{H} = \boldsymbol{GWWG}^T$: a N-by-N kernel matrix (positive semidefinite);<br>• $\boldsymbol{W} = diag(w_1, \ldots, w_k, \ldots, w_K)$, a diagonal matrix of specified SNP weights.<br><br>Q_{SKAT} can be rewritten as:<br>$Q_{SKAT} = \sum_{k=1}^{K} w_k^2 \left[\sum_{i=1}^{N} (y_i - \widehat{\mu_{0,i}}) g_{i,k} \right]$<br><br>H0: $\tau = 0$ ($\Leftrightarrow \boldsymbol{b} = 0$)<br>H1: $\tau > 0$ ($\Leftrightarrow \boldsymbol{b} \neq 0$) (at least one genetic effect)<br><br>Under H0: Q_{SKAT} follows a complicated mixture of χ^2_1 distributions(Kwee *et al.*, 2008).	Based on SKAT function R SKAT (Package 'SKAT' Title SNP-Set (Sequence) Kernel Association Test, 2023) R package with following options:<br><br>SKAT_kernel="linear.weighted",<br>SKAT_weights_beta= c(a_1, a_2)<br><br>$a_1 = 1$ and $a_2 = 25$ which gives very little weight, if any, to the common variants and is used as default in SKAT and SKATO.<br><br>In "region output",<br>columns named:<br>• "SKAT, SKAT.pLiu and SKAT.pDavies" corresponds to SKAT results with $\boldsymbol{W} = \boldsymbol{I_K}$;<br>*P*-values computed using both: an exact method ("davies") and an approximation method ("liu").
SKATO	Combined test of SKAT and burden test (Q_{SKAT-B})	$Q_{SKAT-O} = \rho\, Q_{SKAT} + (1-\rho) Q_{SKAT-B}$	Based on SKAT function from "SKAT(Package 'SKAT' Title SNP-Set

(Lee, Mary J. Emond, *et al.*, 2012)<br><br>(default)	Q_{SKAT-B} power is optimized when SNPs have the same direction of effects and high correlation.<br><br>SKAT-O includes SKAT ($\rho = 1$) and burden tests as special cases ($\rho = 0$).	Where:<br>• $0 \leq \rho \leq 1$, an optimal ρ is computed from a grid search to maximize the power(Lee, Mary J Emond, *et al.*, 2012)<br>• $Q_{SKAT-B} = \left[\sum_{i=1}^{N}(y_i - \widehat{\mu_{0,i}})\left(\sum_{k=1}^{K} w_k g_{i,k}\right)\right]^2$<br><br>Under H0: Q_{SKATO} ~ mixture of χ_1^2 distributions (as for the SKAT statistic) + $\chi^2(1df)$	(Sequence) Kernel Association Test, 2023)" R package with following options:<br>*method*="optimal.adj".<br>*SKAT_kernel*="linear.weighted",<br>*SKAT_weights_beta*=c(1,25)<br><br>"optimal.adj" computes Q_{SKAT-O} *P*-values based on a grid search of ρ and then uses the minimum *P*-value as a test statistic, as previously recommended(SKAT Package).
**Region-level min*P-like* tests[a]**			
GATES<br>(Li *et al.*, 2011)<br><br>(default)	Minimum single-SNP *P*-value corrected for the effective # of SNPs tested in the region using the Extended Simes procedure.	$P_{GATES} = min\left(\frac{m \times p_{(j)}}{m_{(j)}}\right)$<br>Where:<br>• m: effective number of independent *P*-values in the region, with $m = M - \sum_{j=1}^{M}[I(\lambda_j > 1)(\lambda_j - 1)]\lambda_j$, where *I*(x) is an indicator function and $(\lambda_1, \dots, \lambda_j, \dots, \lambda_M)$ correspond to the eigenvalues of the correlation matrix of the single-SNP *P*-values. This correlation matrix is approximated by a sixth-order polynomial function of the pair-wise allelic correlation coefficient of the region-level SNP matrix(Li *et al.*, 2011);<br>• m_j: effective number of independent *P*-values in the region, calculated as for *m* but for the top *j* single-SNP *P*-values in the region.	None
SimpleM<br>(Gao *et al.*, 2008)<br>(default)	Minimum single-SNP *P*-value corrected for the effective # of SNPs tested in the region.	$P_{SimpleM} = 1 - (1 - p_{(1)})^m$<br><br>Where: m= number of eigenvalues from the SNP correlation matrix of the *K* SNPs ***ranked*** by descending order, that explain cumulatively at least *SimpleM_cut* of the SNP variation in the region.	*SimpleMcut*=0.995

[a]SNP effects and their variance-covariances estimated using the generalized "glm" function from the R *stats* package or, when *firthreg* =TRUE from the (CRAN: Package brglm2).

1.2 Summary of region-level tests performances previously reported

Prior literature provides good evidence that the region-level tests we have implemented in *regscan* are sensitive to different underlying region-level genetic architectures (Yoo et al., 2017). This study compares the relative performance of Wald, LC, MLC, PC80, SKAT, SKAT-O and Min-*P* region-level tests in 1,000 genes randomly chosen in the autosomal genome to obtain a representative panel of region sizes and within-region LD. For each gene, 1,000 replicates of a quantitative trait were generated under an additive genetic model (see scenarios 0-6 in Table S2). Under the null hypothesis (with no genetic effects in each region, scenario 0), the Type I error rates for MLC, LC, SKAT and PC80 are close to the nominal level of 0.05, while the Wald, SKAT-O and Min*P* tests tend to be inflated (see (Yoo *et al.*, 2017) for details). Under an alternative hypothesis, in which there is at least one causal variant in each region (scenarios 1-6 in Table S2), MLC, PC80, and SKAT show remarkably similar powers in regions simulated with one or two causal variants with same direction of effects and tend to outperform the Wald test power for most scenarios. However, in scenario 4 where two causal SNPs in the same LD bin have opposite effects, most of the region-level tests lose power relative to the Wald tests compared to scenarios 1-3,5 and 6. In more complex scenario 6 with up to ten deleterious or protective causal SNPs, randomly assigned within a gene, again MLC and PC80 outperform the Wald test and exhibit comparable power, but MLC exhibits the least variability in power regardless of the number of causal SNPs; this suggests that MLC is more robust than PC80 across different region complexities.

Table S2. Scenarios of complexities within regions investigated by (Yoo *et al.*, 2017)

Scenarios	Within region genetic architecture
0	No SNP association
1	One causal SNP within a gene
2	Two causal SNPs in the same LD bin, both deleterious
3	Two causal SNPs in different LD bins, both deleterious
4	Two causal SNPs in the same LD bin, one deleterious, and one protective
5	Two causal SNPs in different LD bins, one deleterious, and one protective
6	Up to ten deleterious or protective causal SNPs, randomly assigned within a gene

In **Supplementary Information 2**, we describe steps involved in variant processing and analysis, as well as options implemented in the main *regscan* function to help diagnose and reduce multicollinearity in the region-level multi-SNP regression models.

<u>Supplementary Information 2</u>. Details on the main and auxiliary functions

2.1 *regscan*: main function to process and analyze regions

2.1.1 Steps involved in processing and analysis of each region

In **Fig. S1**, we summarize the main steps implemented in *regscan* from data processing to analysis of each region, with details described thereafter.

<u>Fig. S1</u>. Overview of the steps in *regscan*

Step1: SNP recoding

- **recoding** of all the SNPs such that each genotype corresponds to the number of copies of the minor allele
- If multiallelic = TRUE, each multi-allelic variant is coded as multiple biallelic variables for each minor allele, where each genotype corresponds to the number of copies of the corresponding minor allele

Step2: SNP filtering/selection

- **filtering** of the SNPs with MAF < *mafcut* (default *mafcut*= 0.05)

Step3: Clustering & Recoding (for the MLC test)

- **Clustering of the bialleic SNPs into LD bins** (default *edgecut*=0.5);
- **Recoding of the biallelic SNPs** to maximize the # of SNP pairs with positive correlation **within each LD bin;**
- If multiallelic = TRUE, assign each of the biallelic SNP alternative allele of each multiallelic-SNP to a LD bin based on correlation.

Step4: SNP pruning

if LDpruning=TRUE (optional): **Pruning of *highly* correlated SNPs** ($|r| > rcut$, default *rcut*=0.99);

Pruning of the SNPs in complete linear dependency (aliasing) to reduce fitting issues in multi-SNP regional regression models.

Step5: Region-level & SNP-level analysis

- Region-level & single-SNP tests applied to all *K* remaining SNPs after **Step4;**
- if singleSNPall=TRUE: report single-SNP test results applied to all the SNPs kept at **Step2** (including all the SNPs pruned out on LD/complete linear dependency)

Step1. Recoding of all the SNPs such as baseline allele = major allele

In *regscan,* all the SNPs within each region are recoded such that each genotype corresponds to the number of copies of the minor allele, as some of the region-level tests are sensitive to the direction of the SNP effects (**Supplementary Information 1**). When the analysis also includes multi-allelic SNPs (multiallelic=TRUE), then a VCF file name must be specified and *regscan* will use internally *recodeVCF* to automatically create *geno/SNPinfo* inputs for each region and apply an algorithm we implemented to recode the multi-allelic SNPs to create a biallelic SNP variable for each of the minor allele(s); for each biallelic SNP variable each genotype will correspond to the number of copies of the corresponding minor allele (see details in **section 2.2**).

Step2. SNP filtering/selection

Because SNPs with a low minor allele frequency (MAF) can lead to numerical instability in the multi-SNP region-level regression models fitting in studies with small sample sizes, *regscan* prunes out the SNPs with MAF < 0.05 (Step1) by default. However, for studies with large sample sizes, the default *mafcut* value can be relaxed or specified according to the minor allele count of each variant.

Step3. SNP clustering and recoding for the constrained MLC region-level test

i. **Clustering of the biallelic SNPs in LD bins** is based on the Pearson correlation coefficient metric (see Yoo et *al*(Yoo *et al.*, 2015) for details on the CliQue-Based (CLQ) clustering algorithm implemented in RegionScan). By default, the clustering parameter (*edgecut*) is set to 0.5 based on recommendations from previous numerical experimentations (Yoo *et al.*, 2017); edgecut=0.5 corresponds to LD as defined by r^2 = 0.25.

ii. **Recoding of the biallelic SNPs** to maximize the number of biallelic SNP pairs with **positive correlation** within each LD bin for the MLC region-level test, and within the whole region for the LC region-level test. The variant-level output includes indicator variables to identify the SNPs that have been recoded for MLC (MLC.flip) or for LC (LC.flip) region-level tests (see **section 2.1.3**).

iii. if multiallelic = TRUE: **assign each bi-allelic SNP variable derived from a multi-allelic SNP to a LD bin** based on the correlation with SNPs from each LD bin:
   a. Identify the LD bin(s) with a minimum correlation with the bi-allelic SNP variable ≥ edgecut & assign the biallelic SNP variable to that LD bin;
   b. Otherwise, if no bin matches the condition in a): assign the bi-allelic SNP variable to a new LD bin;
   c. Repeat the previous steps for all remaining bi-allelic SNP variables (note that the binary indicator variables from the same multi-allelic SNP can be assigned to different LD bins).

Step4. Pruning of highly correlated SNPs & SNPs in complete linear dependency

Perfect correlation/high multicollinearity in regions can lead to the inability to estimate the region-level multi-SNP regression models (e.g. ill-conditional region-level genotypes matrix). Therefore, by default in *regscan,* within each region we prune out SNPs with Pearson correlation (in absolute value) larger than 0.99 (*rcut*). The *rcut* value can be adjusted, for example based on the study sample size, and type of trait studied (quantitative or binary). LD pruning can be optionally skipped if LDpruning=FALSE. In any case, aliases are checked before fitting the multi-SNP region-level regression model and removed from each set of analyzed SNPs for the region-level tests. In the variant-level output file, we also report variance inflation factor (VIF) values for each of the SNPs analyzed in region-level multi-SNP regression models to diagnose potential multicollinearity issues. Below, we describe the steps involved in the pruning:

i. **Pruning of the SNPs *highly* correlated within each LD bin** (default *rcut*=0.99):

   If *multiallelic* = FALSE: pruning of the bi-allelic SNPs,

   If *multiallelic* = TRUE:

   a) For the multi-allelic SNPs: Distinguish two sets of multi-allelic SNPs:

      setA: multi-allelic SNPs with *only one* bi-allelic SNP variable;

      setB: multi-allelic SNPs with at *least two* bi-allelic SNP variables.

      Then, prune the SNPs in the following order:

      Prune the bi-allelic SNPs,

      Prune the SNPs in setA

      If a bi-allelic SNP has abs(r)≥*rcut* with a SNP from setA,

      keep the bi-allelic SNP and exclude the setA SNP.

      Prune the SNPs in setB

ii. **Removal of the alias(es) within the region** using the *alias* function from the base R "stats" package.

Step5. Region-level & Single-SNP tests

By default, all the region-level and single-SNP tests are based on the exact same set of K SNPs kept after ***Step4*** to facilitate the comparison of the results across the different tests. If the option singleSNPall=TRUE, the single-SNP results are reported for all the SNPs kept after ***Step1*** (including the later SNPs pruned out based on correlation or complete linear dependency).

<u>Fig. S2</u>. Illustration of the clustering and recoding steps for the SNPs in an arbitrary region on chr16 (region #13 in the artificial dataset provided with the RegionScan package); these plots were produced with the option MLCheatmap=TRUE in the *regsan* function.

Panel (A) shows the heatmap of the SNP correlation matrix at ***Step2***; with SNPs ordered by genomic positions on chr16.

Panel (B) illustrates the heatmap of the SNP correlation matrix at ***Step3***, with the **SNPs ordered by LD bin** (from the largest to the smallest LD bin based on number of SNPs assigned) and by genomic position within each LD bin. Each LD bin is represented by a unique color band on the left and bottom of the heatmap.

Panel (C) illustrates the heatmap of the SNP correlation matrix at ***Step4*** **but before SNP recording**; with **SNPs ordered by LD bin** as in panel B; color bands on the left and bottom of the heatmap match with the colors of the LD bins shown in panel B.

Panel (D) is the same heatmap as panel (C) but **after recording of the SNPs;** color bands on the left and bottom of the heatmap match with the color of the LD bins in panels (B) and (C).

(see **Fig S2** on the next page)

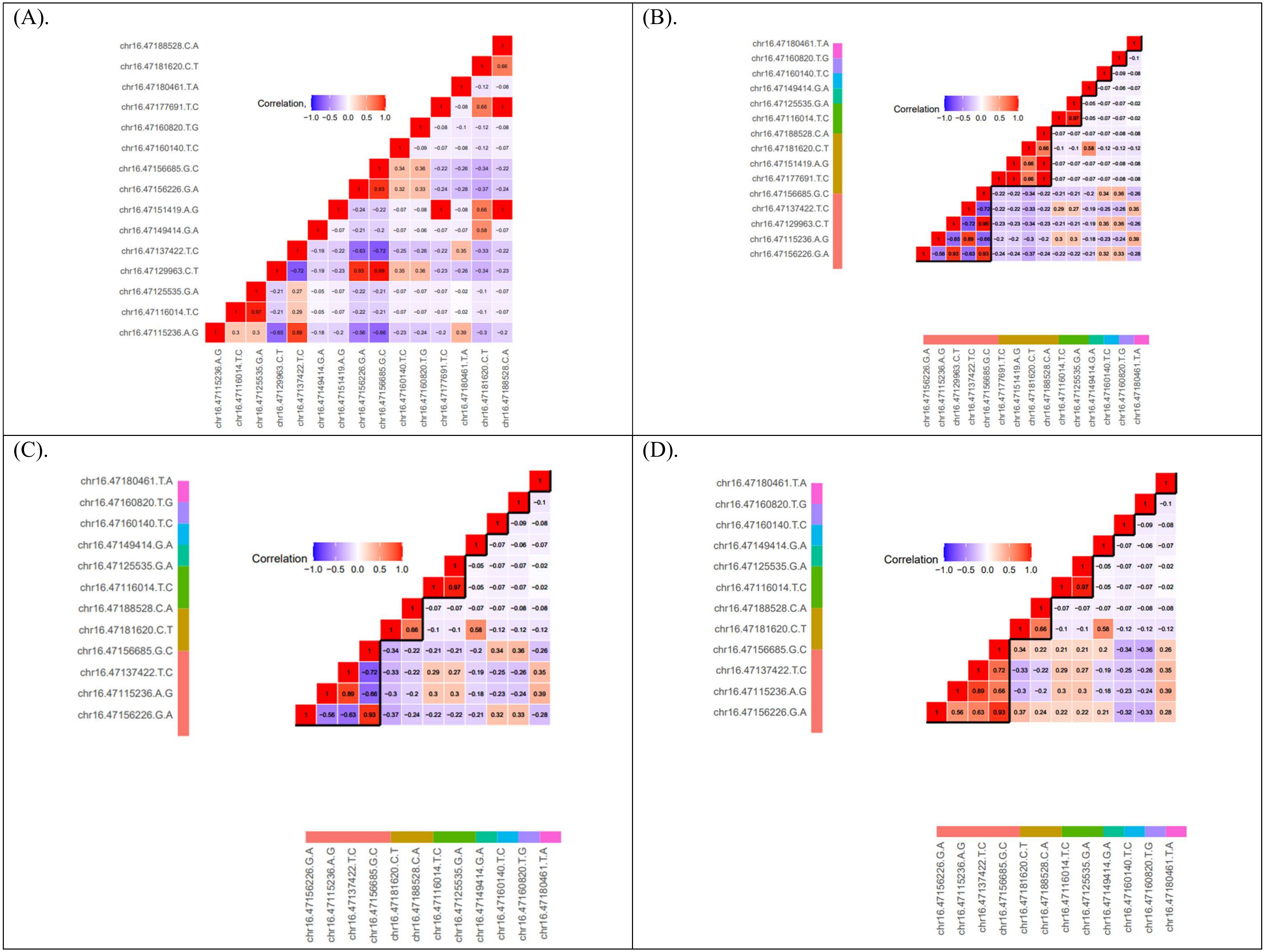
(A).
chr16.47188528.C.A
chr16.47181620.C.T
chr16.47180461.T.A
chr16.47177691.T.C
chr16.47160820.T.G
chr16.47160140.T.C
chr16.47156685.G.C
chr16.47156226.G.A
chr16.47151419.A.G
chr16.47149414.G.A
chr16.47137422.T.C
chr16.47129963.C.T
chr16.47125535.G.A
chr16.47116014.T.C
chr16.47115236.A.G
Correlation
−1.0 −0.5 0.0 0.5 1.0
(B).
chr16.47180461.T.A
chr16.47160820.T.G
chr16.47160140.T.C
chr16.47149414.G.A
chr16.47125535.G.A
chr16.47116014.T.C
chr16.47188528.C.A
chr16.47181620.C.T
chr16.47151419.A.G
chr16.47177691.T.C
chr16.47156685.G.C
chr16.47137422.T.C
chr16.47129963.C.T
chr16.47115236.A.G
chr16.47156226.G.A
Correlation
−1.0 −0.5 0.0 0.5 1.0
(C).
chr16.47180461.T.A
chr16.47160820.T.G
chr16.47160140.T.C
chr16.47149414.G.A
chr16.47125535.G.A
chr16.47116014.T.C
chr16.47188528.C.A
chr16.47181620.C.T
chr16.47156685.G.C
chr16.47137422.T.C
chr16.47115236.A.G
chr16.47156226.G.A
Correlation
−1.0 −0.5 0.0 0.5 1.0
(D).
chr16.47180461.T.A
chr16.47160820.T.G
chr16.47160140.T.C
chr16.47149414.G.A
chr16.47125535.G.A
chr16.47116014.T.C
chr16.47188528.C.A
chr16.47181620.C.T
chr16.47156685.G.C
chr16.47137422.T.C
chr16.47115236.A.G
chr16.47156226.G.A
Correlation
−1.0 −0.5 0.0 0.5 1.0

2.1.2 List of main arguments and default values

Table S3 List of main arguments of *regscan*

Arguments	Definition	Default value
REGIONinfo	dataframe including region positions. This dataframe must include the four following columns: "chr", "region", "start.bp", "end.bp". This file can include LD block regions positions as generated using for example, by the "BigLD" function from the *gpart* R package or any type of genes/regions positions.	Required
phenocov	dataframe including the covariates (if applicable) and the phenotype(s) in columns. Individuals (in rows) must be in the same order as in the *data* input.	
covlist	list of covariates to account for in single-SNP and multiple-SNP regression models, including for example, ancestry PCs and/or non-genetic covariates (must be included in *data* or *phenocov* if applicable)	NULL
covout	option to output the covariate coefficients, standard errors and their *P*-values from multi-SNP region-level regression models (used to construct MLC, LC and Generalized Wald tests)	FALSE
pheno	name of the phenotype column in *phenocov* to consider for the analysis	Required
pheno_type	pheno_type="C" if phenotype is **c**ontinuous,<br>or pheno_type="D" if phenotype is **d**ichotomous	Required
geno_type	Format of the genotypes: geno_type="D" if genotypes are in allele dosage format , or genotype format (geno_type="G")<br>– argument required for SKAT/SKATO tests	Required
***If data/info data frames are specified* (in this case, recodeVCF is not used)**		
data	dataframe that includes the genotypes (in columns) and individuals (in rows)	Required if *vcfname* is left empty
SNPinfo	dataframe that includes the information for each variant (in rows), must include the following columns:<br>"chr", "pos", "variant", "ref", "alt", "multialSNP", "multialSNP.rec","freq.alt"<br>• "multialSNP": binary variable=0, if the SNP is bi-allelic (two alleles), or 1 if more than two alleles<br>• "multialSNP.rec": indicates if the multi-allelic SNP has been recoded b recodeVCF (such that baseline allele = major allele)<br>• "freq.alt": frequency of the alternate allele (minor allele)<br>This input can be generated using the auxiliary function *recodeVCF.*	Required if *vcfname* is left empty
Used only by the auxiliary function recodeVCF ***if data and SNPinfo are not specified***		

vcfname	name of the VCF file (if required)	NULL
qcmachr2	threshold used to filter out SNPs with low Mach R2 imputation quality score	NULL
qcinput	dataframe that includes at least 2 columns: variant & info_score (for filtering based on imputation quality score)	NULL
info_score	threshold used to filter out SNPs with low Info imputation quality score – If this option is specified, "qcinput" must be specified, and "info_score" must be a column in "qcinput"	NULL
Options to include multiallelic SNPs		
multiallelic	If FALSE: extract & process only the biallelic SNPs,<br>If TRUE: include multiallelic SNPs in addition to the biallelic SNPs.	FALSE
multial_nmaxalleles	For extraction of SNPs with fewer than *multial_nmaxalleles* alleles (e.g. 2 to extract bi-allelic SNPs (default),<br>3 to extract bi-allelic and tri-allelic SNPs, etc).	2
Options for pruning/filtering of the SNPs in each region (see details in Supplementary Information 2, section 2.3)		
mafcut	threshold to filter out the SNPs with MAF ≤ *mafcut*	0.05
LDpruning	option to prune out the SNPs within each region based on the absolute value of the region-level genotypes correlation matrix; recommended to reduce the multi-collinearity issues in the multi-SNP region-level regression models (see **section 2.1.2**).	TRUE
rcut	threshold to prune out the correlated SNPs (works only if LDpruning=TRUE)	0.99
Other options		
singleSNPall	Produces an additional output including the single-SNP results (and LD-bin level information) for all the SNPs before LD pruning/alias identification.<br>– can increase computational time.	FALSE
firthreg	If firthreg ="TRUE", use a Jeffreys-prior penalized-likelihood regression implemented in the R package **brlmgm2**(Kosmidis *et al.*, 2020; Kosmidis and Firth, 2021) instead of the default maximum-likelihood logistic regression. This option is recommended for unbalanced case-control data / small minor allele count.	FALSE
parallel	By default, process & run region-level analysis sequentially; if parallel ="TRUE" proceed in parallel.	FALSE
regionlist	list of the region names from REGIONinfo input to be analyzed for the analysis of a subset of regions.	NULL
alltests	By default, output region-level tests: Wald, MLCB, PC80, SKAT, SKATO, LCB, GATES, SimpleM; If alltests="TRUE" reports additional region-level tests: MLCZ, LCZ.	FALSE
edgecut	parameter for clustering of the SNPs in LD bins (based on SNP correlation) using the CLQ algorithm applied before MLC test.	0.5[a]
tol	tolerance parameter to deal with convergence issues in regression models.	1e-16

MLCheatmap	For each region, produces four heatmap plots of the region-level SNP correlation matrix, with SNPs ordered by positions & by LD bin, before and after LD pruning (if applicable) as illustrated in **Fig S2.** We recommend using this option for investigation of a subset of regions of interest specified with the option regionlist.	FALSE
SKAT_kernel	type of kernel used for SKAT and SKATO tests. There are 6 types of pre-specified kernels: "linear", "linear.weighted", "IBS", "IBS.weighted", "quadratic" and "2wayIX". See "kernel" argument in SKAT function in https://cran.r-project.org/web/packages/SKAT/SKAT.pdf for details.	linear.kernel
SKAT_weights	a numeric vector of weights for the weighted kernels. When it is NULL, the beta weight with the "SKAT_weights_beta" parameter is used.	NULL
SKAT_weights_beta	a numeric vector of parameters for the beta weights for the weighted kernels. If you want to use your own weights, please use the "SKAT_weights" parameter. It will be ignored if "weights" parameter is not null.	c(1,25)

[a]By default, edgecut=0.50 (which corresponds to r2=0.25), showed to be the optimal clustering parameter value in previous simulation studies (Yoo *et al.*, 2017).

2.1.3 Description of the main outputs of *regscan* function

Table S4. Region-level output includes region positions, and region-level test results for all regions analyzed.

Column name	Description
chr	chromosome # (as provided in *REGIONinfo* input)
region	region # or name (as provided in *REGIONinfo* input)
start.bp	start region position in bp (as provided in *REGIONinfo* input)
end.bp	end region position in bp (as provided in *REGIONinfo* input)
NSNPs	NSNPs in region (before pruning on LD/perfect linear dependency)
NSNPs.kept	NSNPs analyzed in region (after pruning on LD/perfect linear dependency)
max.VIF	Maximum VIF value among the NSNPs.kept
Wald	Generalized Wald statistic
Wald.df	degree of freedom of the Generalized Wald statistic (= NSNPs.kept)
MLCB	MLCB test statistic
MLCB.df	degree of freedom of the MLCB test (= # of LD bins in the region)
MLCB.p	*P*-value for the MLCB test
LCB	LCB test statistic
LCB.df	degree of freedom of the LCB test (=1 for all the regions)
LCB.p	*P*-value for the LCB test
PC80	PC80 test statistic
PC80.df	degree of freedom of the PC80 test
PC80.p	*P*-value for the PC80 test
SKAT	SKAT statistic
SKAT.pDavies	*P*-values from SKAT computed using Davies's method
SKAT.pLiu	*P*-values from SKAT computed using Liu's method
SKATO.p	SKATO test statistic

GATES.df	degree of freedom for the GATES test
GATES.p	*P*-value for the GATES test
SimpleM.p	*P*-value for the simpleM test
single_Wald.p	Minimum *P*-value from single-SNP analysis among the NSNPs.kept
**If alltests=TRUE**	
MLCZ	MLC statistic based on Z-scores from multi-SNP region-level regression model<br>(rather than on SNP effects as used for MLCB)
MLCZ.p	*P*-value for MLCZ test statistic from multi-SNP region-level regression model<br>(rather than on SNP effects as used for MLCB)
LCZ	LC statistic based on Z-scores (rather than SNP effects) from multi-SNP region-level regression model<br>(rather than on SNP effects as used for MLCB)
LCZ.p	*P*-value for LCZ test statistic

Table S5. Bin-level output (specific to the MLC test), includes the LDbin specific results within regions analyzed

Column name	Description
chr	chromosome # (as provided in input *REGIONinfo)*
region	region # or name (as provided in input *REGIONinfo)*
start.bp	start region position in bp (as provided in input *REGIONinfo)*
end.bp	end region position in bp (as provided in input *REGIONinfo)*
bin	LDbin # assigned within each region (bin #1 corresponds to the bin with largest bin.size)
bin.size	# of SNPs in LD bin
bin.size.keptSNPs	# of SNPs in LD bin (after pruning on MAF & LD (if applicable),<br>and complete linear dependency)
deltabinB	Bin-level test statistic
deltabinB.p	*P*-value for 1 df Wald test of deltabinB
**If alltests=TRUE**	
deltabinZ	Same as deltabinB but using Z statistics instead of Betas
deltabinZ.p	Same as deltabinB but using Z statistics instead of Betas

Table S6. Variant-level output including variant information/positions as well as LD bin assignation and estimates from single-SNP and multiple-SNP regression models applied within each region

Column name	Description
chr	chromosome # (as provided in *REGIONinfo* input)
region	region # or name (as provided in *REGIONinfo* input)
start.bp	start region position in bp (as provided in *REGIONinfo* input)
end.bp	end region position in bp (as provided in *REGIONinfo* input)
variant	variant name (as provided in *SNPinfo* input)
pos	SNP position (as provided in *SNPinfo* input)

multiallelicSNP	indicator variable to flag multiallelic SNPs (as provided in *SNPinfo* input)
ref	Reference allele (as specified in *SNPinfo* input)
alt	Alternate allele (as specified in *SNPinfo* input)
maf	Minor allele frequency
LDbin	LDbin # assigned within each region (numbered by decreasing # of SNPs)
LDbin.size	# of SNPs analyzed in each LDbin (kept after pruning on MAF & LD)
MLC.flip	Flag the SNPs recoded for MLC/LCbin tests
LC.flip	Flag the SNPs recoded for the LCbin tests
sg.beta	SNP effect estimate from single-SNP regression models
sg.pval	*P*-value for 1 *df* Wald test of SNP effect from single-SNP regression models
VIF	Variance inflation factor (VIF) values based on *all SNPs* analyzed in each region
glm.beta	SNP effect estimate from the region-level multi-SNP regression model
glm.pval	*P*-value for 1 *df* Wald test of SNP effect the region-level multi-SNP regression model

Table S7. List of SNPs excluded from the region-level tests and reasons for exclusion

**Column name**	**Description**
chr	Chromosome # (as provided in input *REGIONinfo)*
region	region # (as provided in input *REGIONinfo)*
start.bp	end position in bp (as provided in input *REGIONinfo)*
end.bp	start position in bp (as provided in input *REGIONinfo)*
variant	variant name (as provided in input *REGIONinfo)*
multiallelicSNP	indicator variable to flag multiallelic SNPs (as provided in input *REGIONinfo)*
pos	SNP position (as provided in input *SNPInfo)*
MAF	Minor allele frequency
reason	Reason of exclusion:<br>"mafcut" – MAF < *mafcut* (if applicable)<br>"rcut" – high correlation (if applicable)<br>"alias" – complete linear dependency<br>"multial" – multiallelic SNP (if applicable)

Table S8. Optional output produced if singleSNPall=TRUE. This output includes single-SNP results and bin-level information for all SNPs in regions before LD pruning (kept at ***Step3***)

**Column name**	**Description**
chr	Chromosome # (as provided in input *REGIONinfo)*
region	region # (as provided in input *REGIONinfo)*
start.bp	end position in bp (as provided in input *REGIONinfo)*
end.bp	start position in bp (as provided in input *REGIONinfo)*
variant	variant name (as provided in input *SNPinfo*)
pos	SNP position (as provided in input *SNPinfo*)
multiallelicSNP	indicator variable to flag multiallelic SNPs (as provided in input *SNPinfo*)

major.allele	Major allele (as provided in input *SNPinfo*)
minor.allele	Minor allele (as provided in input *SNPinfo*)
maf	Minor allele frequency
LDbin	Bin #; within each region (numbered by decreasing # of SNPs)
sg.beta	SNP effect estimate from single-SNP regression models
sg.pval	*P*-value for 1 *df* Wald test of SNP effect from single-SNP regression models
rmcorr	Indicator variable to flag SNPs excluded because of LD pruning

2.2 *recodeVCF*: auxiliary function to extract and process SNPs in VCF files

In this section, we describe the algorithm implemented in *recodeVCF* to extract (or calculate) the allele dosage and recode the bi-allelic SNPs (and multi-allelic SNPs, if *multiallelic*=TRUE) from an **unfiltered** VCF type 4 file format that includes all the imputation probabilities for all the SNPs to produce the inputs (*data* and *SNPinfo*) for *regscan*.

2.2.1 Processing of the bi-allelic SNPs (multiallelic = FALSE)

By default, the options in *regscan/recodeVCF* are set to extract the allele dosage of the bi-allelic SNPs from the VCF file and to recode the allele dosage (or genotypes) of each variant such as the baseline allele corresponds to the major allele and then keep the variants with a minor allele frequency > *mafcut*.

2.2.2 Processing of multi-allelic SNPs (multiallelic = TRUE)

If one of the alternate alleles is the major allele, then updating the allele dosage such that the baseline allele matches with the other allele is required to account for all the imputation probabilities at all the biallelic SNP variables for that multi-allelic SNP. Below, we describe the pseudo-algorithm implemented in *recodeVCF* for the multi-allelic SNPs.

We assume that each multi-allelic SNP is coded in the VCF file as a set of bi-allelic SNP variables, with the same genomic position and reference allele, which is typically provided from imputations in recent reference panels, such as 1000 Genomes (used in our illustration presented in **Supplementary Information 3**).

For each individual, the imputation programs typically return two imputation probabilities for each of the biallelic SNP variables that comprise the multi-allelic SNP. The two imputation probabilities correspond, respectively, to the:

- probability that individual *i* has one copy of the alternate allele,
- probability that individual *i* has two copies of the alternate allele (see examples in **Tables S9** and **S10**).

However, the reference allele does not always match the major allele, depending on the reference panel used for imputation, and rationalization is needed as described in what follows.

Table S9. Illustration of a fictitious tri-allelic SNP (M=3 alleles) coded as two bi-allelic SNP variables with the same position and the same baseline allele A_1, but different alternate alleles (A_2 and A_3). The allele frequencies of all 3 alleles are assumed to sum to 1 (ie $f(A_1) + f(A_2) + f(A_3) = 1$)

Tri-allelic SNP	Chr:position	Alleles Alternate/reference	Frequency of alternate allele
snp1_A2	19:500003	A_2/A_1	$f(A_2)$
snp1_A3	19:500003	A_3/A_1	$f(A_3)$

Table S10. Example of calculation of the allele dosage for each alternate allele of the tri-allelic SNP from **Table S9** for an individual *i.*

SNP	Imputation probabilities	probability that individual i has	Dosage of alternate allele
snp1_A2	$p_i^{A_2 A_m}, m \epsilon \{A_1, A_3\}$	***exactly one*** copy of A_2 (*ie.* either A_2A_1 or A_2A_3)	$d_i(\boldsymbol{A_2}) = p_i^{A_2A_m} + 2p_i^{A_2A_2}$
	$p_i^{A_2A_2}$	***two copies*** of A_2 (*ie.* A_2A_2)	
snp1_A3	$p_i^{A_3 A_m}, m \epsilon \{A_1, A_2\}$	***exactly one*** copy of A_3 (*ie.* either A_3A_1 or A_3A_2)	$d_i(\boldsymbol{A_3}) = p_i^{A_3A_m} + 2p_i^{A_3A_3}$
	$p_i^{A_3A_3}$	***two copies*** of A_3 (*ie.* A_3A_3)	

If one of the alternate alleles is the major allele, then updating the allele dosage such that the baseline allele matches with the other allele is required to account for all the imputation probabilities at all the biallelic SNP variables for that multi-allelic SNP. Below, we describe the pseudo-algorithm implemented for multi-allelic SNPs to check if the major allele is the baseline allele and calculate the allele dosage for the new alternate allele if the baseline does not match.

Pseudo-algorithm used in *recodeVCF* to recode multi-allelic SNPs

Input files: unfiltered VCF file (type 4.0) including all the imputation probabilities for all SNPs in the region tested (or chromosome)

For each multi-allelic SNP with M alleles, including the baseline allele (can be for example the major allele or reference allele from the reference sequence genome) and M-1 alternate alleles. The illustrative example from **Table S9**, has M=3 alleles.

For *each* multi-allelic SNP,

1) Compute the allele frequency of the **baseline allele**, noted A_1, with:

$$\mathrm{f}(A_1) = 1 - \sum_{m=2}^{M} f(A_m)$$

2) Rank the allele by frequencies:

$$\boldsymbol{f(A_1^*)} >\ldots> f(A_m^*) > \cdots > f(A_M^*)$$

Where A_m^* with $1 \le m \le M$ denote the alleles ranked by frequency

(ie. A_1^* corresponds to the *most* frequent allele, A_M^* to the *least* frequent)

3) **If** { $A_1^* = A_1$},

then { no update of the allele dosage ; **go** to the next multiallelic SNP }

else {

New BASELINE = $\boldsymbol{A_1^*}$

New ALT = A_1 & calculate the dosage of "New ALT" for each individual *i:*

$$d_i\,(A_1) = 2\left(1 - \sum_{m=2}^{M} p_i^{A_m A_m}\right) - \sum_{\substack{m=2 \\ m' \neq m}}^{M} p_i^{A_m A_{m'}}$$

Where:

- $p_i^{A_m A_m}$ are the imputation probabilities for individual *i*, for all the homozygous genotypes $A_m A_m$ at the multi-allelic SNP considered
- $p_i^{A_m A_{m'}}$ are the imputation probabilities for individual *i*, for heterozygous genotypes for each allele A_m at the multi-allelic SNP considered

go to the next multi-allelic SNP

}

For the multi-allelic SNPs where the allele frequency and dosage values have been updated (and all other SNPs), we also report the machr2 imputation quality score calculated as the ratio of the empirically observed variance of the allele dosage to the expected binomial variance at Hardy-Weinberg equilibrium(Marchini and Howie, 2010), for each SNP (with MAF, noted *p*) as:

$$Mach\ r^2 = \begin{cases} \dfrac{\frac{\sum_{i=1}^{N} {d_i}^2}{N} - \left(\frac{\sum_{i=1}^{N} d_i}{N}\right)^2}{2p\,(1-p)} \text{ when } p \in\,]0,1[\\ 1 \qquad\qquad \text{when } p = 0 \text{ or } p = 1 \end{cases}$$

This quality score can be used to filter the multiallelic SNPs that have been recoded, as well as all other biallelic SNPs.

In the example above, from **Table S9** and **Table S10**, if $A_1^*= A_2$, then:

New BASELINE = A_2

New ALT = A_1 then: $d_i\ (A_1) = 2(1 - p_i^{A_2A_2} - p_i^{A_3A_3}) - (p_i^{\boldsymbol{A_2}A_m} + p_i^{\boldsymbol{A_3}A_m})$

2.2.3 Description of outputs

- *data* output, with dosage for bi-allelic & multi-allelic SNPs in region VCF input file, where the multi-allelic SNPs are recoded such that their baseline allele is the major allele

- *SNPinfo* output includes the columns "chr", "pos", "variant","ref", "alt", "region", "start.bp", "end.bp", "multialSNP" which equals to 0 if the SNP is bi-allelic (two alleles), or 1 otherwise (if the SNP has more than two alleles), "multialSNP.rec" indicates if the multi-allelic SNP has been recoded (such that baseline allele = major allele), "freq.alt" corresponds to the frequency of the alternate allele (minor allele), "machr_r2" is the mach imputation quality score calculated for all the SNPs (can be used to filter multiallelic SNPs and all other SNPs that have been recoded).

2.3 Vignette and illustration of the main functions in a small example

https://github.com/brossardMyriam/RegionScan/blob/main/vignettes/RegionScan.pdf (see Annex)

Supplementary Information 3. Application in the DCCT/EDIC genetic study

3.1 DCCT/EDIC analysis methods

To demonstrate RegionScan capabilities (and practical application), we conducted a genome-wide region-level association analysis in *N*=1340 individuals of European-ancestry from the DCCT/EDIC Genetics study (The DCCT Research Group, 1986; Shamoon et al., 1999; Paterson et al., 2010) genotyped with the HumanCoreExome Bead Array with ungenotyped autosomal SNPs imputed using 1000 Genomes (The 1000 Genomes Project Consortium, 2015) phase 3 (v5) and minimac3 (Das *et al.*, 2016) version v.1.0.13, as previously described (Roshandel *et al.*, 2018). For this usage report, we selected the quantitative lipid trait, LDL-C (measured at baseline, untransformed) which is known to have genome-wide polygenic architecture. A large-scale meta-analysis (201 studies of n= 1.65 million individuals) of the Global Lipids Genotyping Consortium(Graham *et al.*, 2021); reports 1,765 distinct index variants that reached genome-wide significance.

To define the regions for comprehensive analysis of the autosomal genome (including intergenic regions), we applied BigLD/gpart(Kim *et al.*, 2018, 2019) with default parameter values separately to each autosome. In total, BigLD partitioned 5,687,207 bi-allelic autosomal SNPs (MAF > 0.05 and Mach imputation quality score $R^2 > 0.5$) into 89,003 quasi-independent non-overlapping autosomal regions of varying sizes adaptive to the LD structure (**Table S11**). We applied RegionScan in genome-wide analysis of the 89,003 regions using the default region-level tests implemented in *regscan*, including sex, age, and sex by age interaction as covariates. In accordance with previous GWAS conducted in DCCT/EDIC individuals of European ancestry, we did not adjust for any ancestry PCs. In **Table S11**, we report on the number of regions and SNPs considered for chromosomes 1-22.

Altogether, the 89,001 regions analyzed include a total of 5,682,968 biallelic SNP variables (ie 5,673,694 biallelic SNPs plus 9,274 biallelic SNP variables corresponding to 4,637 triallelic SNPs processed with *recodeVCF*); while two regions (in chromosomes 6 and 16) were not reported due to fitting issues related to matrix inversion in multi-SNP regional regression models due to complex LD structures. Region sizes (in terms of number of SNPs analyzed) were very variable, with a large majority of the regions including fewer than 100 SNPs (**Fig S3**).

Our genome-wide significance criteria for region-level testing in the DCCT analysis is *P*-value < 5.62E-7 (ie, Bonferroni-correction for 89,001 regions tested), and we consider *P*-value < 1E-5 to be suggestive association. Bonferroni adjustment will be appropriate when genome-wide regions are quasi-independent, which we examined using Q-Q plots of region-level test statistics (**Fig S5**). To further validate genome-wide T1E control for a Bonferroni-corrected significance threshold for number of regions tested, we computed empirical permutation significance thresholds in a large independent sample of 17,000 European-ancestry individuals (CLSA study, (Raina *et al.*, 2019)). We applied the same BigLD partitioning method as in DCCT analysis, obtaining 92,300 regions, and the same region-level analysis pipeline. Results of 1000 lipid trait permutations used to calculate family-wise error rate (FWER) at the level of 0.05 for each of Wald, MLC, PC80, and SKATO region-level tests suggest that the Bonferroni correction for the number of BigLD-defined regions is a valid/appropriate approach for FWER control in a European-ancestry population.

<u>Table S11.</u> Number of regions identified by BigLD in DCCT/EDIC, analyzed with *regscan* and number of variants in regions out of the 5,682,968 SNP variables

chr	# regions	Region size in bp Mean [Min ; Max]	#SNPs within regions[1]	#SNPs analyzed[1,2]
1	7,085	28,910 [2 ; 1,159,157]	434,442	130,554
2	7,075	31,330 [2 ; 808,451]	477,995	152,044
3	5,899	31,172 [2 ;1314,045]	408,142	126,468
4	5,768	30,639 [2 ; 703,033]	420,633	118,029
5	5,300	31,523 [2 ; 1,022,494]	364,563	110,959
6	5,220	30,320 [2 ; 711,485]	387,425	111,695
7	4,996	28,743 [2 ; 1,309,785]	336,169	105,481
8	4,653	28,676 [2 ; 1,522,918]	318,786	102,738
9	4,074	24,949 [2 ; 631,503]	244,427	87,388
10	4,465	27,079 [2 ; 902,714]	294,838	92,182
11	4,236	29,133 [2 ; 1,2345,539]	289,324	87,981
12	4,252	28,834 [2 ; 1,013,631]	276,420	86,484
13	3,232	27,981 [2 ; 689,896]	212,951	64,863
14	2,898	27,896 [2 ; 596,532]	183,245	58,673
15	2,886	24,630 [2 ; 761,812]	158,021	55,364
16	3,332	20,187 [2 ; 498,792]	170,822	66,849
17	2,919	23,684 [2 ; 777,204]	146,123	49,213
18	2,830	24,518 [2 ; 542,101]	160,707	52,487
19	2,548	19,289 [2 ; 587,416]	122,236	41,450
20	2,474	22,075 [2 ; 362,193]	124,006	43,439
21	1,330	22,844 [2 ; 362,193]	79,143	25,832
22	1,531	19,173 [2 ; 509,158]	72,550	26,412
**Total**	**89,003**		**5,682,968**	**1,796,585**

[1]Based on 89,001 regions analyzed with *regscan* (analysis of two regions failed because of convergence issues on chromosomes 6 and 16).

[2]after default pruning on MAF>0.05 and LD (SNP correlation > 0.99) within regions by *regscan*.

Fig S3. Distribution of region sizes (# of SNPs analyzed per region) among the 89,001 regions analyzed genome-wide.

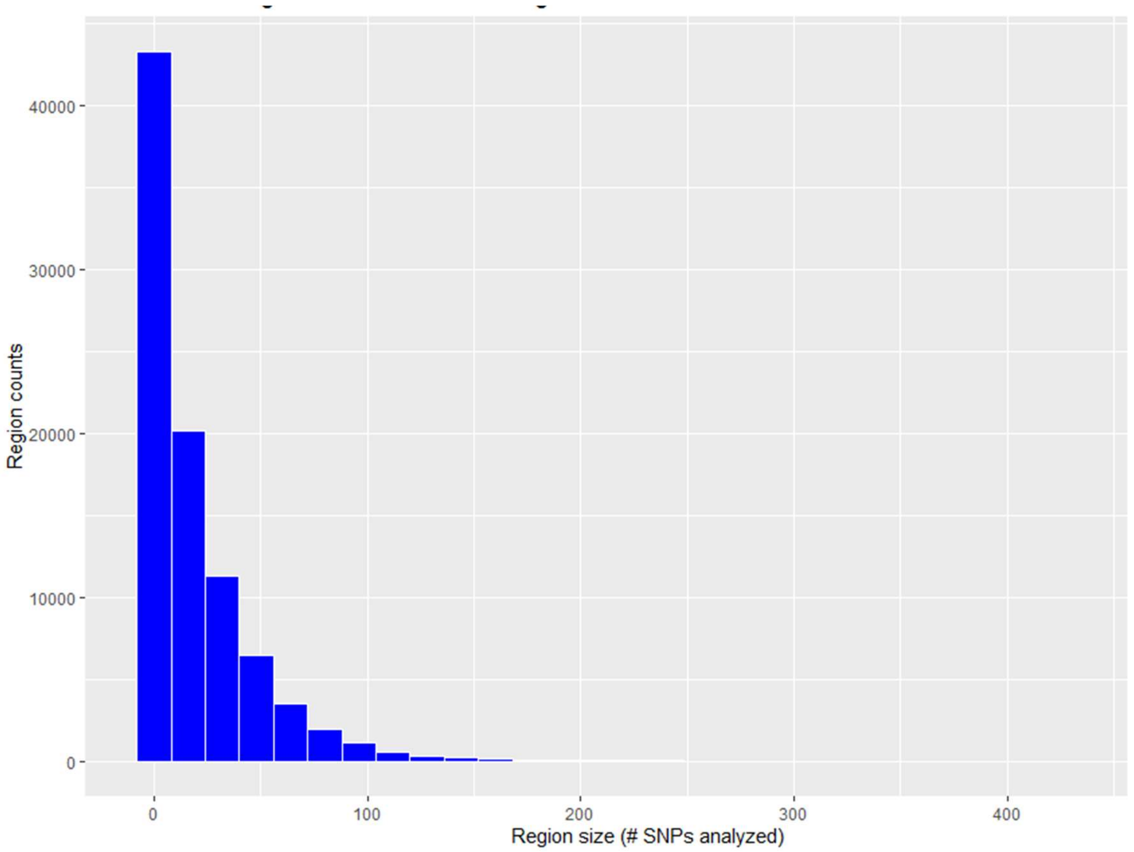

3.2 Results

In **Fig S4**, we report DCCT/EDIC results produced by *regscan* for the MLC region-level test (upper panel), in comparison to the single-SNP results (bottom panel). Out of the 89,001 regions analyzed, we detected five regions around the *APOE* gene that meet the genome-wide region level significance threshold of 5.62E-7 (and 17 single-SNP tests that reach the threshold of 5E-8), and two regions around the *LDLR* gene that reach the suggestive significance threshold of 1E-5 (and 42 single-SNP tests with *P*≤1E-5, none of these SNPs reached the genome-wide significance level). These two loci are located on chromosome 19 and were reported in the large meta-analysis of the Global Lipids Genotyping Consortium(Graham *et al.*, 2021). **Sections 3.3.1 and 3.3.2**, subsequently illustrate the results for extended loci around the detected regions in *APOE* (chr19: 45257201- 45436657) and *LDLR* (chr19: 10962974- 1280183) loci.

<u>Fig S4</u>. Example of "Miami plot" produced by the utility function *MiamiPlot* based on the region-level MLC test P-values (top panel) and variant-level (bottom panel) P-values (-log 10). On the top panel, we show the region-level results for 89,001 regions analyzed, and 1,796,585 SNPs analyzed (after pruning) in these regions. The signals highlighted in orange meet the genome-wide significance criteria: of 5.62E-07 for the region-level tests (dashed line on the top plot), and 5E-8 for single-SNP tests (dashed line on the bottom plot).

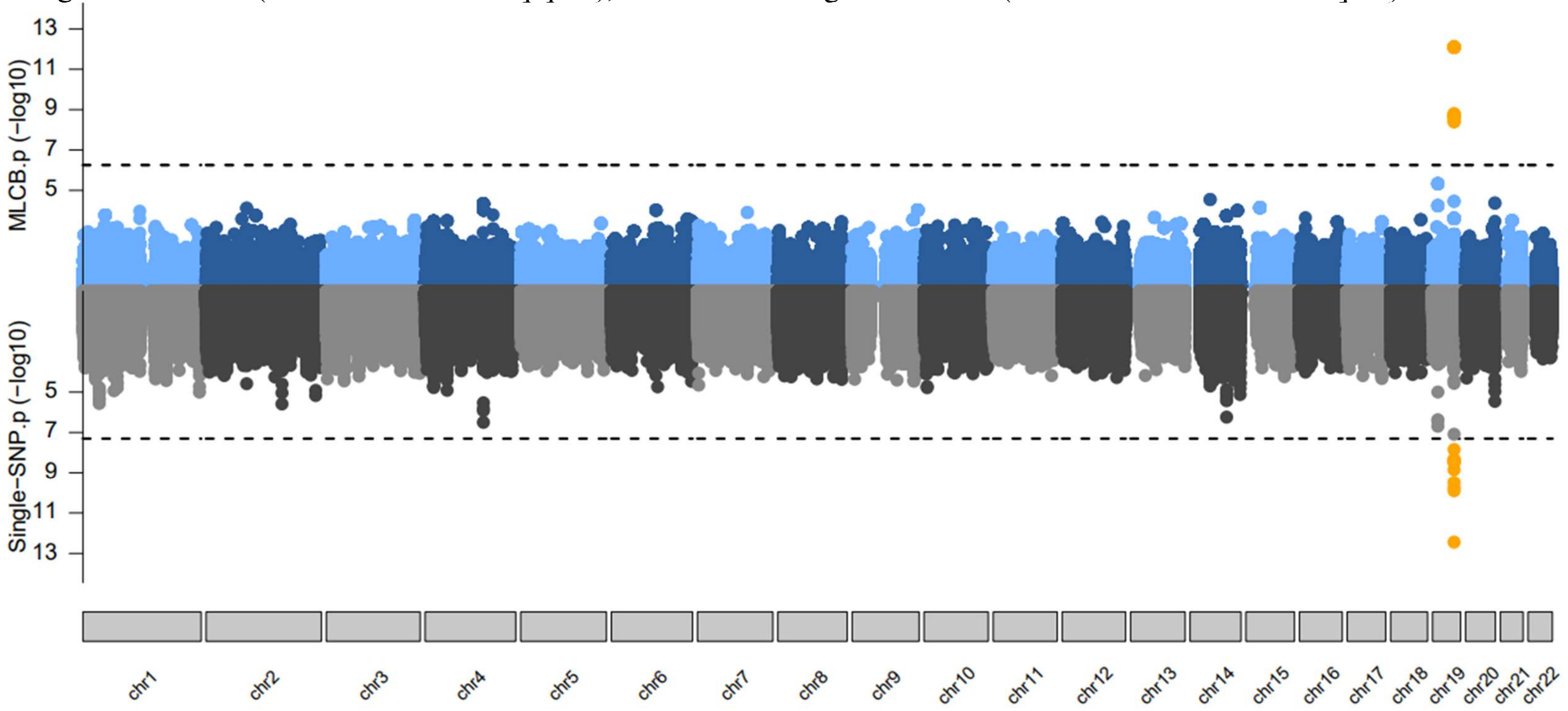

<u>Fig S5</u>. QQ-plots for all the 89,001 regions analyzed with LDL-C in DCCT/EDIC for four region-level tests. These plots were obtained using the *qqregscanPlot* function.

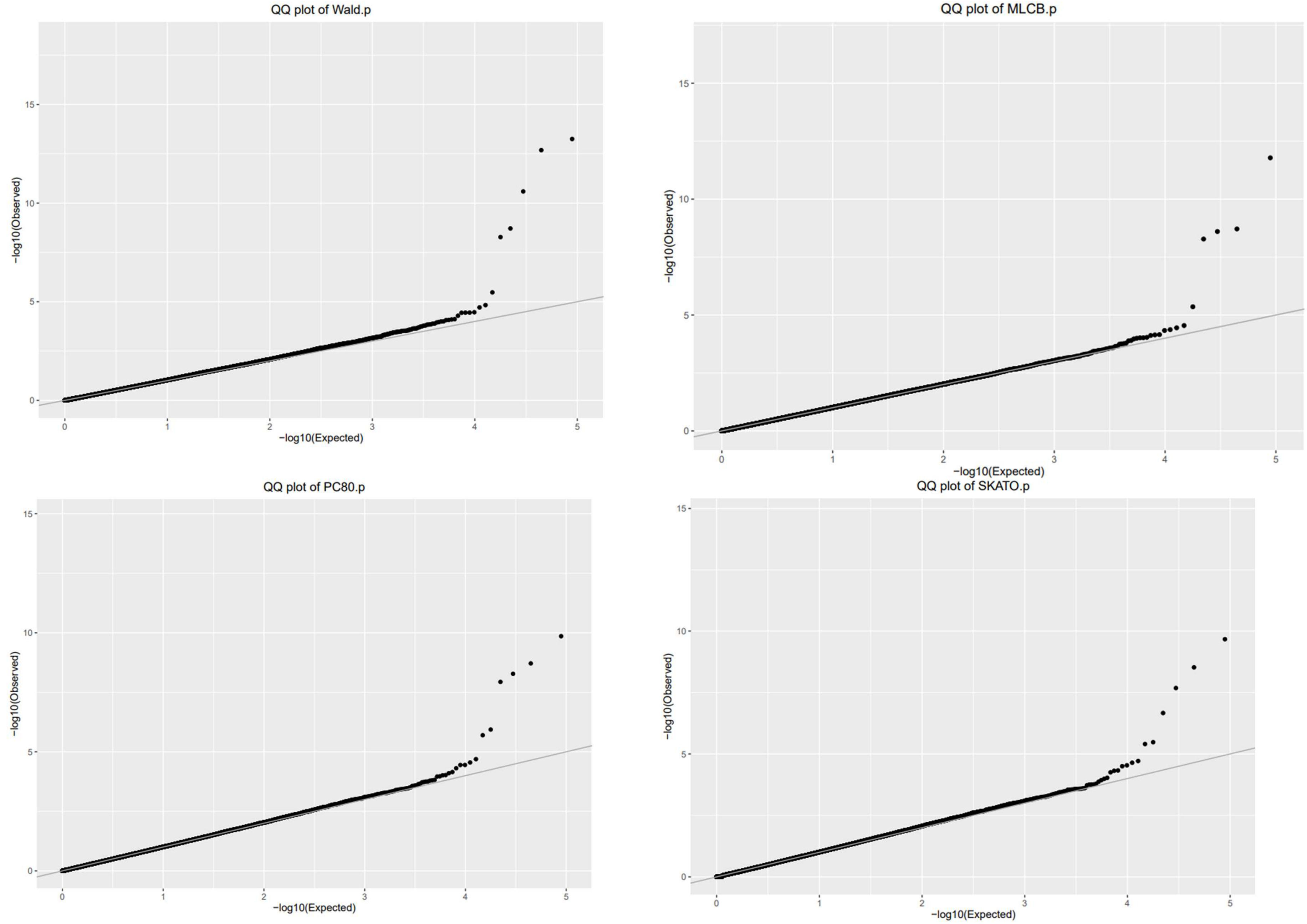

<u>Table S12</u>. Genomic control values for all 89,001 regions analyzed with LDL-C in DCCT/EDIC for the four region-level tests shown in **Fig S5**. These values were obtained using the *qqregscanPlot* function.

**Region-level tests**	**Genomic control**
Wald	1.026
MLC	1.002
PC80	1.00
SKATO	0.955

Table S13. Regions exhibiting association signals on chr19 (+ neighboring regions) extracted from the region-level output of *regscan*. Genome-wide significance criteria for region discovery is test p-value < 5.62E-07 (Bonferroni-correction for 89,001 regions tested), and we consider p < 1E-05 to be suggestive association. The threshold for the conventional single-SNP test is p-value < 5E-08.

chr	region	start.bp	end.bp	nSNPs	nSNPs.kept	maxVIF	Region-level tests							Smallest Single-SNP tests (in the region)
							Wald		MLC		PC80		SKATO.p	
							df	P	df	P	df	P		
19	620	10962974	11069264	120	40	1480.5	40	9.8E-02	6	9.2E-02	3	2.4E-01	2.8E-01	1.2E-02
19	621	11069908	11071395	6	2	1.2	2	2.4E-01	2	2.4E-01	2	2.4E-01	1.8E-01	9.2E-02
19	622	11071560	11157423	106	30	16605.8	30	8.9E-02	3	1.5E-01	2	1.7E-01	1.2E-01	2.3E-03
19	623	11159076	11185014	67	23	772.9	23	6.2E-03	7	4.1E-03	4	1.1E-01	7.6E-02	1.2E-02
19	624	11185919	11202306	51	8	86.8	8	1.3E-04	3	**4.4E-06**	2	**1.2E-06**	**3.3E-06**	1.9E-07
19	625	11205975	11214533	20	7	105.1	7	1.3E-03	4	1.3E-04	2	2.0E-05	2.8E-04	1.1E-05
19	626	11216561	11228745	26	14	755.1	14	2.8E-01	4	8.9E-02	2	1.7E-02	1.1E-02	2.1E-03
19	627	11228783	11229577	5	2	1.2	2	6.9E-03	2	6.9E-03	2	6.9E-03	9.5E-03	3.5E-03
19	628	11229765	11250396	105	35	20886.1	35	1.2E-02	8	1.5E-01	3	1.1E-02	4.2E-03	9.7E-04
19	629	11253310	11262319	25	21	176.5	21	8.7E-02	9	6.7E-02	4	1.2E-02	1.4E-01	2.1E-03
19	630	11262477	11280183	72	26	1681.6	26	3.4E-01	6	1.5E-01	3	4.9E-02	4.3E-02	4.2E-03
19	1683	45257201	45320386	60	31	347.27	31	1.6E-01	7	2.2E-01	4	1.1E-01	5.2E-01	1.1E-01
19	1684	45322744	45353261	93	49	4365.55	49	1.5E-02	9	3.7E-03	4	6.7E-03	1.3E-02	4.1E-05
19	1685	45354044	45354296	3	2	1.28	2	3.6E-05	2	3.6E-05	2	3.6E-05	2.9E-04	2.9E-05
19	1686	45355595	45359706	19	9	528.08	9	7.6E-03	4	5.8E-03	3	3.5E-02	1.0E-02	8.0E-04
19	1687	45360573	45360762	3	3	5.51	3	1.8E-01	2	3.1E-01	2	3.2E-01	5.2E-01	1.3E-01
19	1688	45360967	45360968	2	1	1.00	1	6.3E-01	1	6.3E-01	1	6.3E-01	6.3E-01	6.3E-01
19	1689	45361224	45384116	98	40	699.20	40	2.2E-04	9	7.0E-05	4	8.9E-01	3.5E-01	6.8E-03
19	1690	45385759	45415935	61	38	356.11	38	**5.6E-14**	11	**1.7E-12**	5	**1.4E-10**	**4.0E-06**	**6.1E-13**
19	1691	45416178	45418790	6	4	58.30	4	**2.1E-13**	2	5.1E-02	2	1.0E-01	**2.2E-07**	**1.8E-08**
19	1692	45421254	45424514	9	6	47.82	6	**2.6E-11**	2	**2.5E-09**	2	**1.2E-08**	**2.2E-10**	**1.7E-10**
19	1693	45425178	45426792	3	2	1.04	2	**1.9E-09**	2	**1.9E-09**	2	**1.9E-09**	**3.0E-09**	**9.6E-08**
19	1694	45427125	45428234	3	2	1.12	2	**5.3E-09**	2	**5.3E-09**	2	**5.3E-09**	**2.1E-08**	**4.6E-09**
19	1695	45428459	45430280	3	3	4.68	3	4.0E-01	1	9.6E-01	1	9.8E-01	6.3E-01	5.1E-01
19	1696	45431453	45436657	11	4	53.66	4	1.1E-02	2	1.0E-02	2	8.9E-03	3.8E-03	6.4E-04

3.3 Two regions exhibiting association signals

3.3.1 "APOE locus" (chr19: 45257201- 45436657)

<u>Fig S6.</u> LD (r2) heatmap of the 14 consecutive BigLD regions in chr19: 45257201- 45436657 (regions #1683 to #1696) produced using the *LDblockHeatmap* function from the *gpart* package (for all biallelic SNPs satisfying QC and analyzed in BigLD). Protein coding gene positions are shown in Genome Build 37.

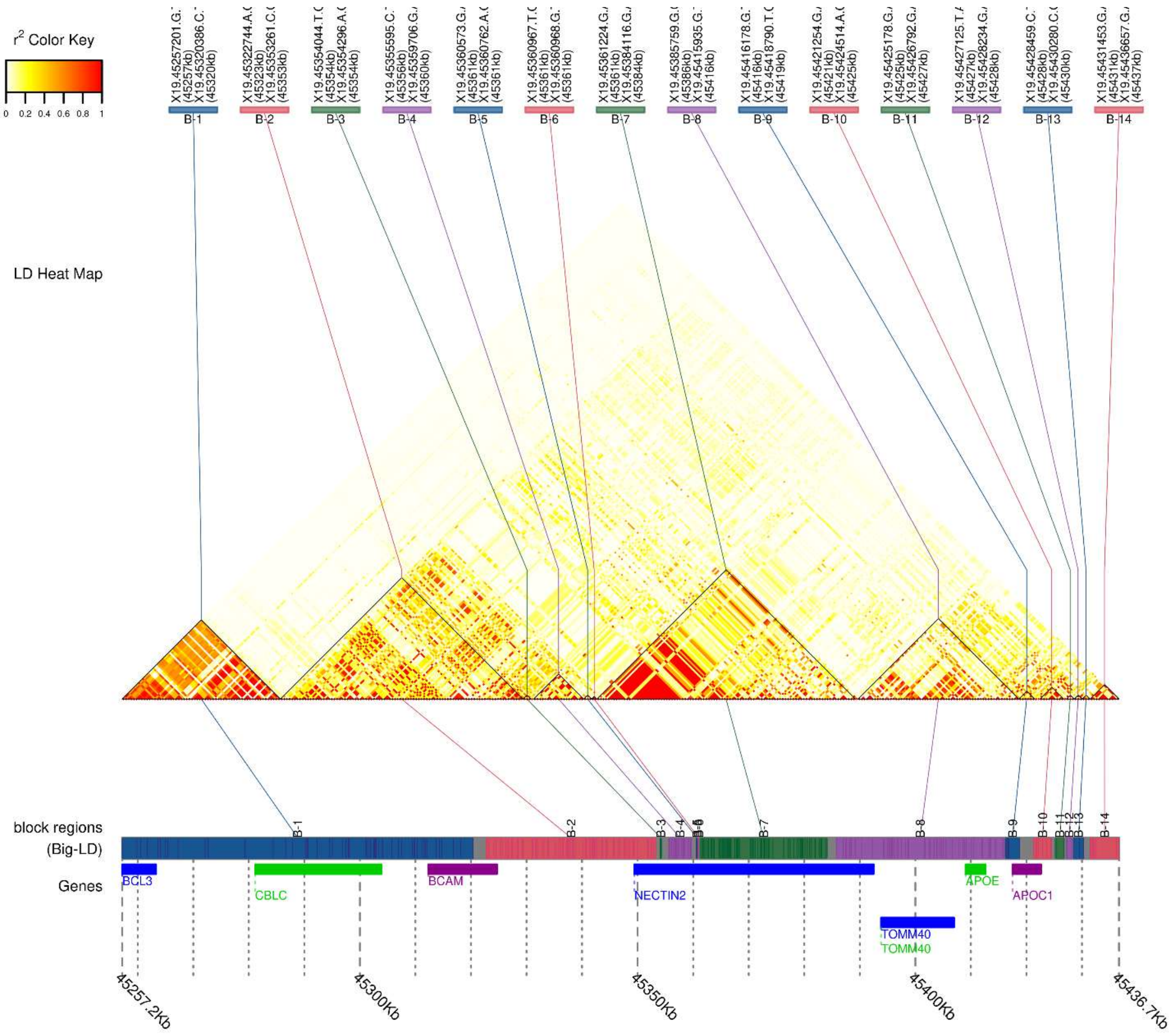

<u>Fig S7.</u> Multi-region association plot for regions #1683 to #1696 in chr19. The *Y* axis shows the P-values (-log10) for four region-level tests and all single-SNP tests along the base pair positions on chr19 (*X* axis). Dashed horizontal lines indicate GW significance thresholds: 5E-8 for single-SNP analysis (blue dotted line) and the genome-wide region-level significance threshold of 5.62E-7 (red dotted line). Region numbers appear at the bottom of the plots.

In Panel (A), the physical positions of the regions (and SNPs) are displayed on the X axis, with regions shaded.

In Panel (B), the regions are shown at a fixed, arbitrary size for all regions to facilitate comparisons of the results across them. The boundaries of the regions are indicated by alternating shading. The labels for each region test show the number of SNPs analyzed within each region. To help visualize the relative differences in association signals across tests, lines connect consecutive regions for each region-level test. The number of SNPs analyzed in each region is reported on the corresponding region bands.

(A).

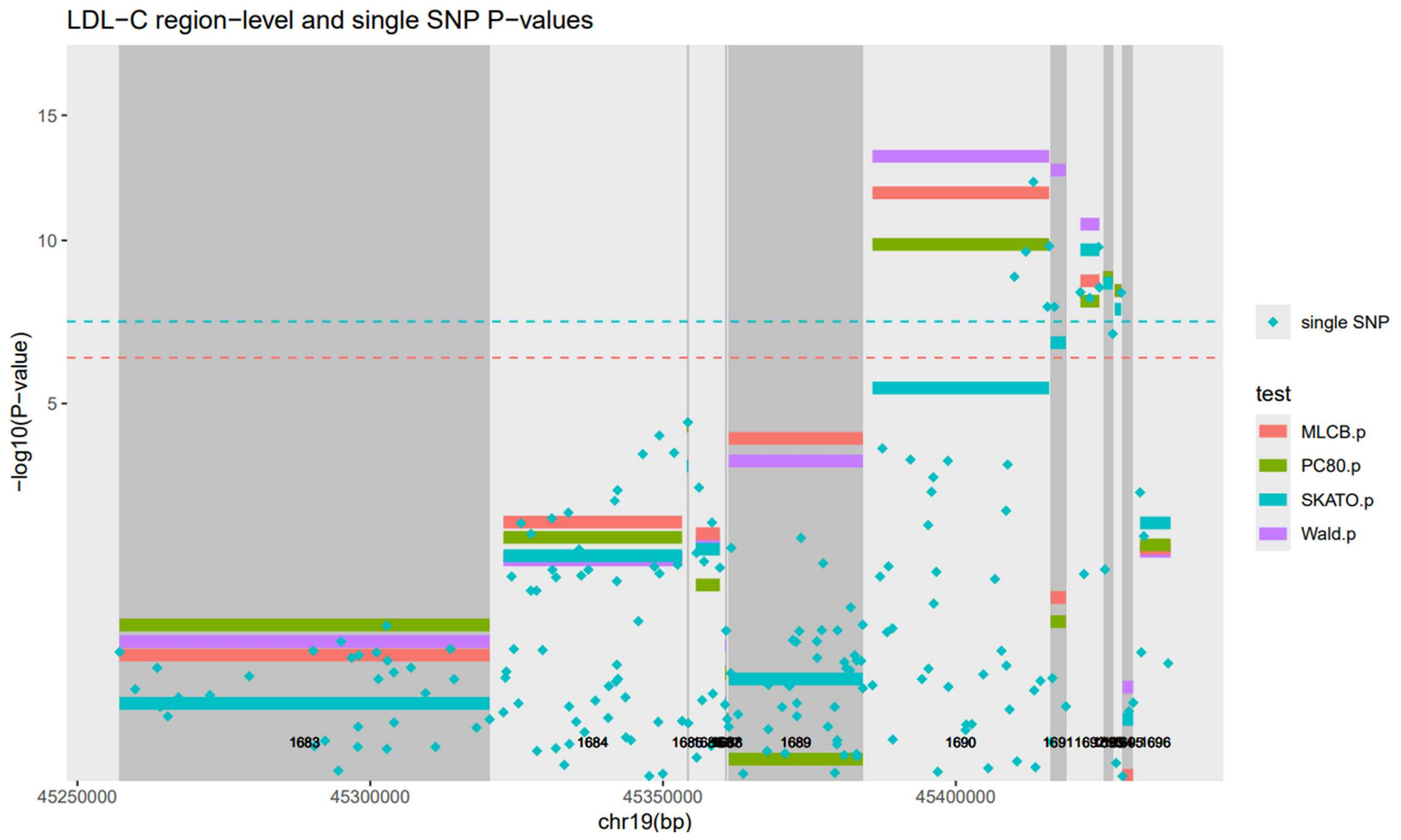
LDL−C region−level and single SNP P−values
-log10(P-value)
15
10
5
1683
1684
1689
1690
1696
45250000
45300000
45350000
45400000
chr19(bp)
single SNP
test
MLCB.p
PC80.p
SKATO.p
Wald.p

(B).

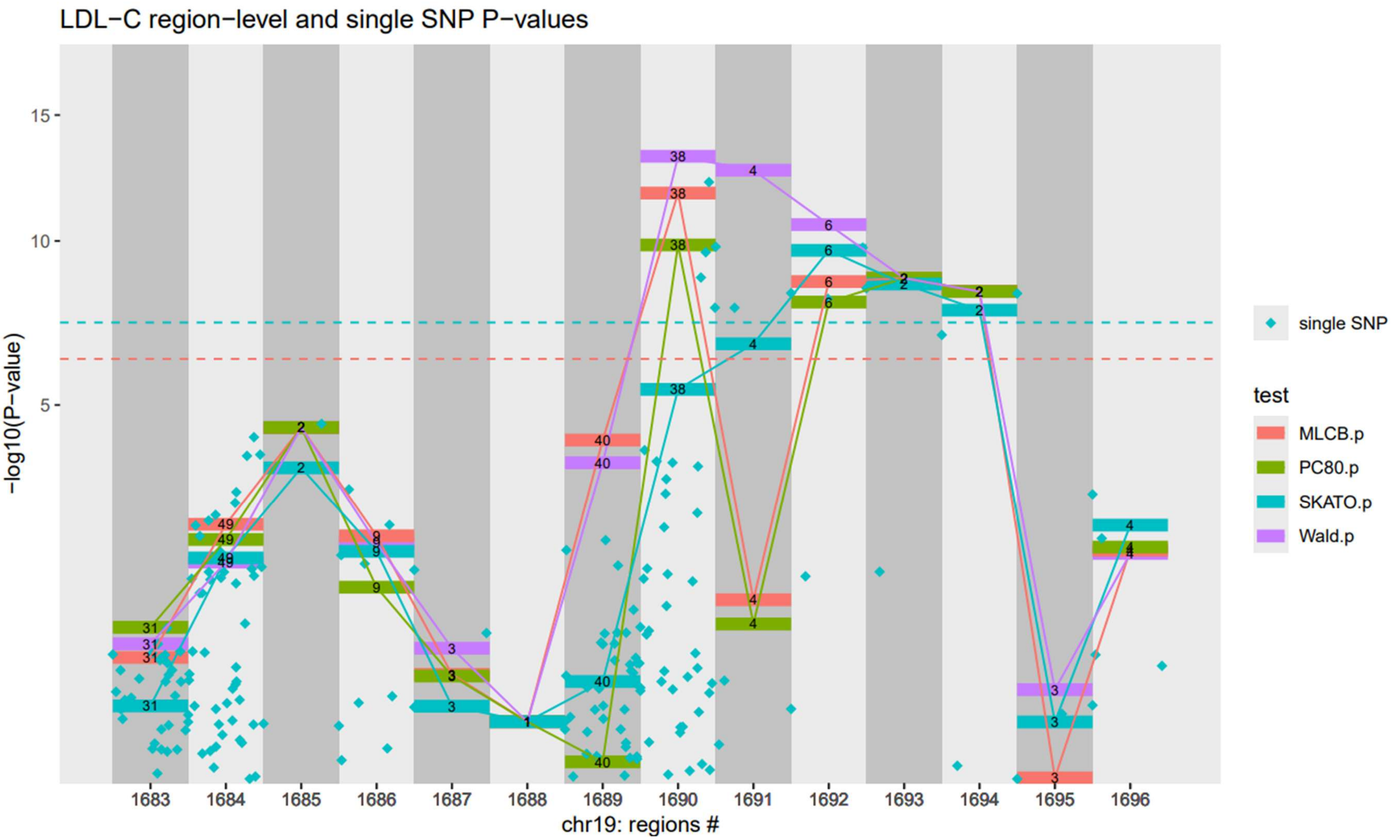

LDL−C region−level and single SNP P−values
-log10(P-value)
15
10
5
chr19: regions #
1683
1684
1685
1686
1687
1688
1689
1690
1691
1692
1693
1694
1695
1696
single SNP
test
MLCB.p
PC80.p
SKATO.p
Wald.p

<u>Fig S8.</u> Visualization of the correlation structure **within region #1690** for the SNPs analyzed (after pruning & recoding), with SNPs ordered by physical position (A), and ordered by LD bins (B); SNPs part of the same LD bin are indicated by the same color bands. Panel (C) shows the physical position of the SNPs in region #1690 by LD bins (*Y* axis); the colors of the LD bins match those shown in Panel (B), SNPs pruned out are shown in grey (with counts before/after pruning).

(A).

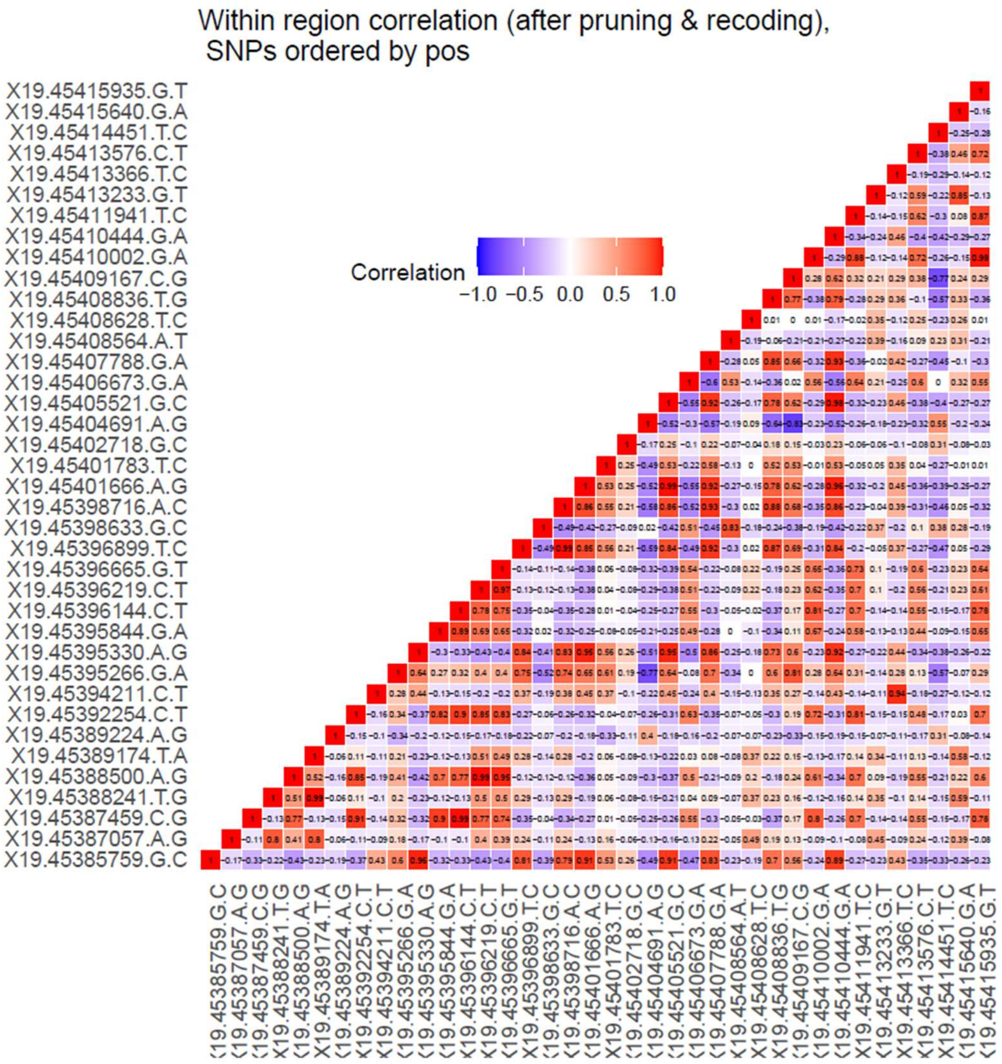

(B).

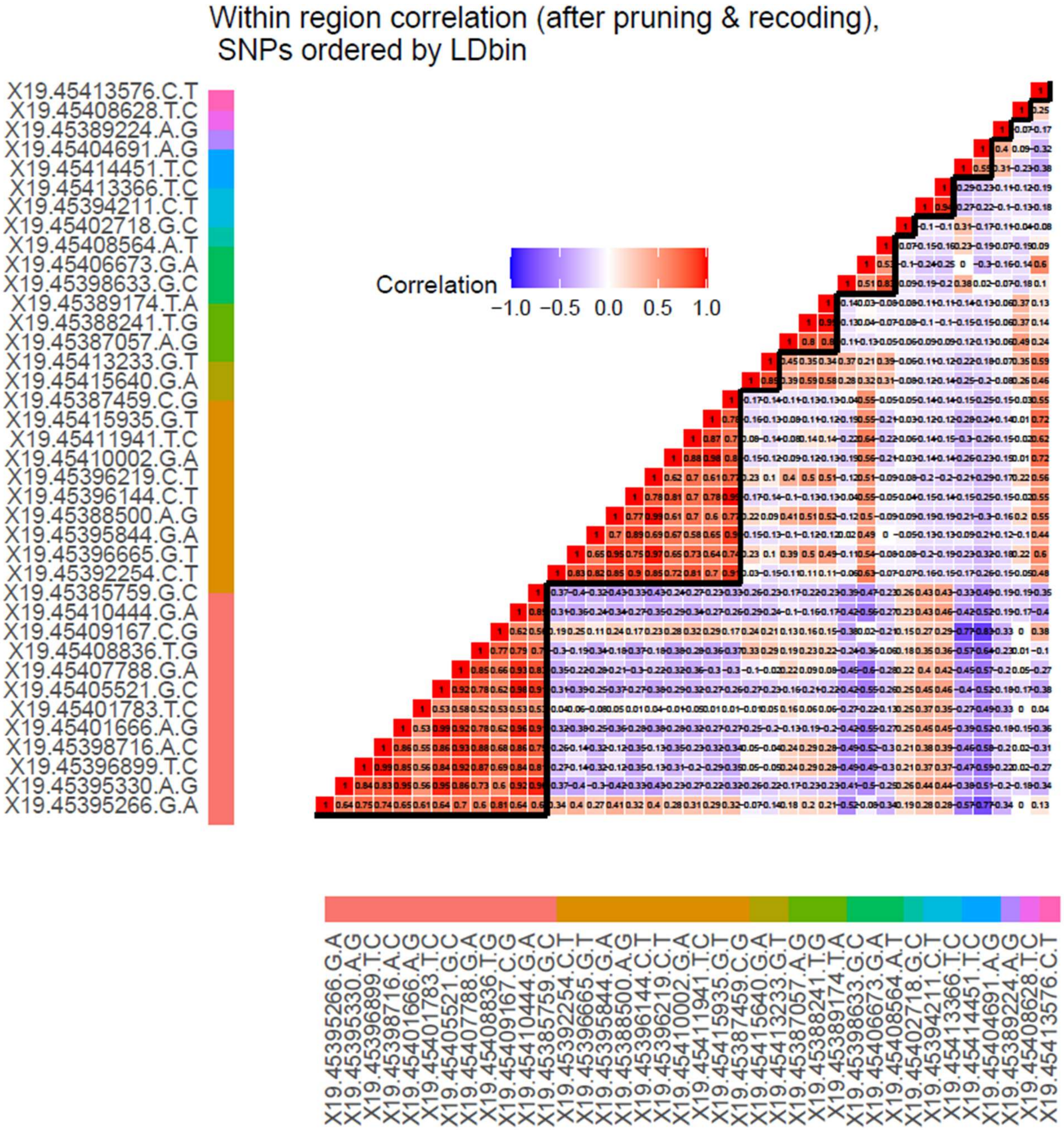
Within region correlation (after pruning & recoding),
SNPs ordered by LDbin
Correlation
−1.0 −0.5 0.0 0.5 1.0
X19.45413576.C.T
X19.45408628.T.C
X19.45389224.A.G
X19.45404691.A.G
X19.45414451.T.C
X19.45413366.T.C
X19.45394211.C.T
X19.45402718.G.C
X19.45408564.A.T
X19.45406673.G.A
X19.45398633.G.C
X19.45389174.T.A
X19.45388241.T.G
X19.45387057.A.G
X19.45413233.G.T
X19.45415640.G.A
X19.45387459.C.G
X19.45415935.G.T
X19.45411941.T.C
X19.45410002.G.A
X19.45396219.C.T
X19.45396144.C.T
X19.45388500.A.G
X19.45395844.G.A
X19.45396665.G.T
X19.45392254.C.T
X19.45385759.G.C
X19.45410444.G.A
X19.45409167.C.G
X19.45408836.T.G
X19.45407788.G.A
X19.45405521.G.C
X19.45401783.T.C
X19.45401666.A.G
X19.45398716.A.C
X19.45396899.T.C
X19.45395330.A.G
X19.45395266.G.A

(C).

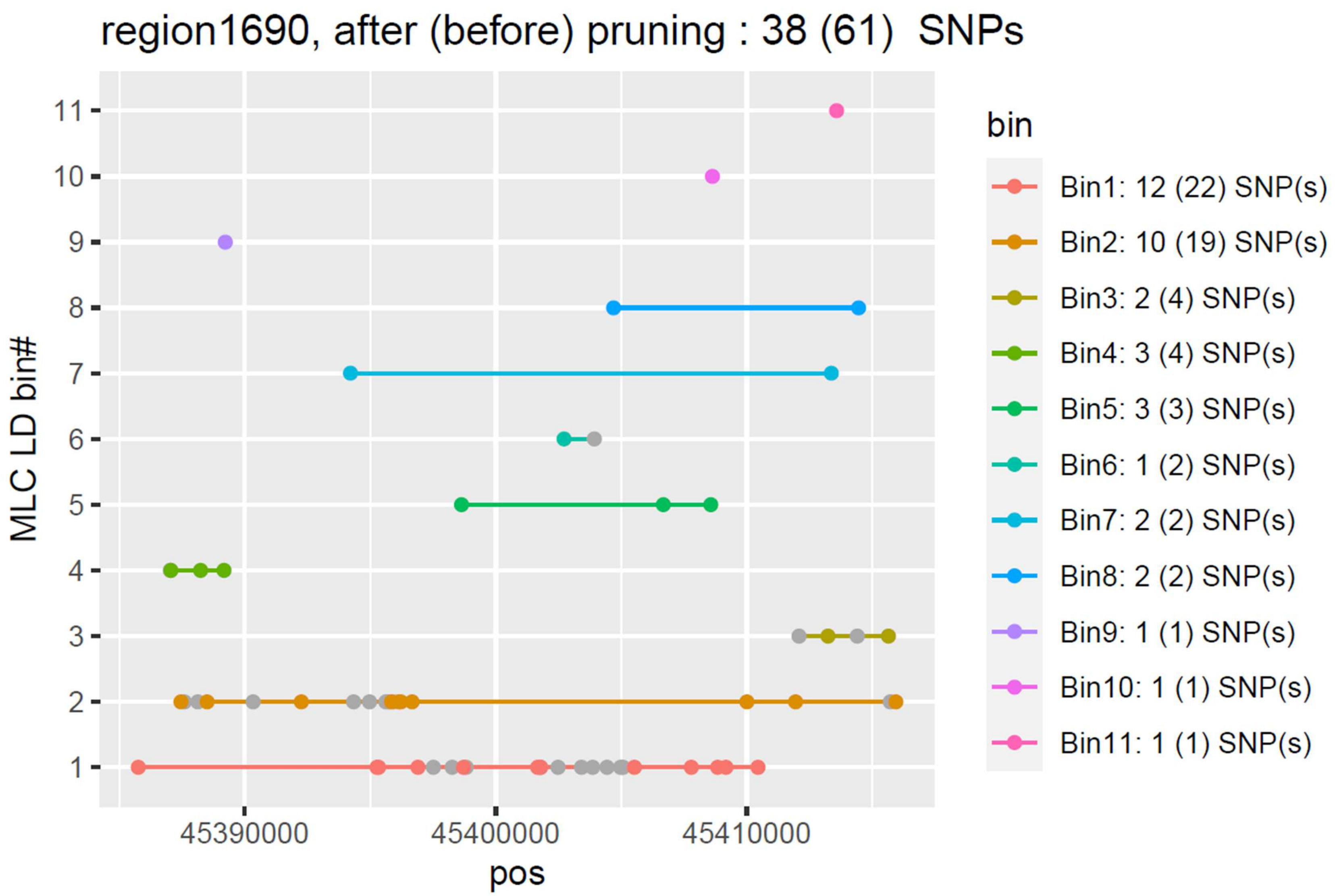
region1690, after (before) pruning : 38 (61) SNPs
MLC LD bin#
11
10
9
8
7
6
5
4
3
2
1
45390000
45400000
45410000
pos
bin
Bin1: 12 (22) SNP(s)
Bin2: 10 (19) SNP(s)
Bin3: 2 (4) SNP(s)
Bin4: 3 (4) SNP(s)
Bin5: 3 (3) SNP(s)
Bin6: 1 (2) SNP(s)
Bin7: 2 (2) SNP(s)
Bin8: 2 (2) SNP(s)
Bin9: 1 (1) SNP(s)
Bin10: 1 (1) SNP(s)
Bin11: 1 (1) SNP(s)

Example: Detailed results for region #1690

Region #1690 is identified as the top region by at least three of the region-level tests (*P*<5.62E-7) and by single-SNP analysis (*P*<5E-8). This region overlaps *APOE* and *TOMM40*, and includes the two well-known *APOE* SNPs, rs429358 and rs7412, reported associated with LDL-C (Rasmussen-Torvik *et al.*, 2012; Sanna *et al.*, 2011). The Wald region-level test has a smaller *P*-value than MLC, PC80 and SKATO region-level tests (**Table S14**). Subsequent investigations of the MLC-based bin-level test results in region #1690 show that rs429358 and rs7412 are assigned to two different LD bins (bin #2 and #3, **Tables S15** and **S16**). Both SNPs have single-SNP *P*-values < 5E-8 (**Table S16**) in addition to their proxies; however, MLC-bin-level results show association for LD bin #3 (**Table S15**) but not for LD bin #2 likely related to the complex *APOE* haplotype effects in this locus.

Table S14. Region-level test results in region #1690 (extract from the "region-level" output)

chr	region	start.bp	end.bp	nSNPs	nSNPs.kept	maxVIF	Wald		MLC		PC80		SKATO.p	ACAT.p[1]
							df	P	df	P	df	P		
19	1690	45385759	45415935	61	38	356.11	38	**5.6E-14**	11	**1.7E-12**	5	**1.4E-10**	**4.0E-06**	**2.2E-13**

[1]*P*-value based on the Aggregated Cauchy Association Test (ACAT,(Liu *et al.*, 2019)) applied to *P*-values from for Wald, MLC and PC80 region-level tests. ACAT.p is not reported by *regscan but* can be easily computed from the region-level output of *regscan* using the "ACAT" R package (https://github.com/yaowuliu/ACAT).

Table S15. MLC-based LD Bin-level test results in region #1690 (extract from the "bin-level" output)

chr	region	LD bin	binstart.bfP .(bp)	binend.bfP.(bp)	binstart.afP.(bp)	binend.afP.(bp)	binsize. bfP	binsize.afP	deltaB	deltaB.se	deltaB.p value
19	1690	1	45,385,759	45,410,444	45,385,759	45,410,444	22	12	-0.52	0.34	1.2E-01
19	1690	2*	45,387,459	45,415,935	45,387,459	45,415,935	19	10	-0.26	0.34	4.4E-01
**19**	**1690**	**3***	**45,412,079**	**45,415,640**	**45,413,233**	**45,415,640**	**4**	**2**	**-11.93**	**1.99**	**2.1E-09**
19	1690	4	45,387,034	45,389,174	45,387,057	45,389,174	4	3	2.69	1.18	2.2E-02
19	1690	5	45,398,633	45,408,564	45,398,633	45,408,564	3	3	0.41	0.94	6.6E-01
19	1690	6	45,402,718	45,403,924	45,402,718	45,402,718	2	1	2.87	3.33	3.9E-01
19	1690	7	45,394,211	45,413,366	45,394,211	45,413,366	2	2	0.95	1.29	4.6E-01
19	1690	8	45,404,691	45,414,451	45,404,691	45,414,451	2	2	-4.19	2.01	3.7E-02
19	1690	9	45,389,224	45,389,224	45,389,224	45,389,224	1	1	2.08	2.92	4.8E-01
19	1690	10	45,408,628	45,408,628	45,408,628	45,408,628	1	1	-0.07	2.78	9.8E-01
19	1690	11	45,413,576	45,413,576	45,413,576	45,413,576	1	1	5.33	3.10	8.5E-02

*bin2 includes rs429358 and bin3 includes rs7412; combination of alleles C at rs429358 and allele C at rs7412 is known as the APOE-e4 allele which has been reported associated with LDL-C and increased risk for heart disease.

Table S16. Single-SNP test results in region #1690 (extract from the "variant-level" output) for the 38 variants kept after LD pruning.

chr	region	LD bin	variant	rsID	multiallelic	ref	alt	maf	MLC. Codechange[1]	Single-SNP analysis sglm. Beta	sglm. Pvalue
19	1690	1	19.45385759.G.C	rs3745150	0	G	C	0.41	0	1.02	3.9E-01
19	1690	1	19.45395266.G.A	rs157580	0	G	A	0.38	1	-3.23	4.1E-03
19	1690	1	19.45395330.A.G	rs2075649	0	A	G	0.39	0	1.20	2.9E-01
19	1690	1	19.45396899.T.C	rs157584	0	T	C	0.49	0	0.07	9.5E-01
19	1690	1	19.45398716.A.C	rs157590	0	A	C	0.48	0	-0.94	4.0E-01
19	1690	1	19.45401666.A.G	rs8106922	0	A	G	0.41	0	-0.42	7.1E-01
19	1690	1	19.45401783.T.C	rs56290633	0	T	C	0.44	1	-0.56	6.7E-01
19	1690	1	19.45405521.G.C	rs1305062	0	G	C	0.40	0	-0.09	9.3E-01
19	1690	1	19.45407788.G.A	rs7259620	0	G	A	0.45	0	-1.44	2.0E-01
19	1690	1	19.45408836.T.G	rs405509	0	T	G	0.48	1	4.08	2.7E-04
19	1690	1	19.45409167.C.G	rs440446	0	C	G	0.35	1	-0.68	5.6E-01
19	1690	1	19.45410444.G.A	rs769450	0	G	A	0.40	0	-0.14	9.0E-01
19	1690	2	19.45387459.C.G	rs12972156	0	C	G	0.14	0	6.15	1.2E-04
19	1690	2	19.45388500.A.G	rs283811	0	A	G	0.22	0	3.23	1.9E-02
19	1690	2	19.45392254.C.T	rs6857	0	C	T	0.17	0	5.45	2.1E-04
19	1690	2	19.45395844.G.A	rs34095326	0	G	A	0.11	0	5.76	9.8E-04
19	1690	2	19.45396144.C.T	rs11556505	0	C	T	0.14	0	5.41	5.0E-04
19	1690	2	19.45396219.C.T	rs157582	0	C	T	0.22	0	2.50	6.2E-02
19	1690	2	19.45396665.G.T	rs17855927	0	G	T	0.21	0	3.13	2.3E-02
**19**	**1690**	**2**	**19.45410002.G.A**	**rs769449**	**0**	**G**	**A**	**0.12**	**0**	**10.03**	**1.8E-09**
**19**	**1690**	**2**	**19.45411941.T.C**	**rs429358**	**0**	**T**	**C**	**0.15**	**0**	**9.85**	**2.5E-10**
**19**	**1690**	**2**	**19.45415935.G.T**	**rs7256200**	**0**	**G**	**T**	**0.12**	**0**	**10.91**	**1.6E-10**
**19**	**1690**	**3**	**19.45413233.G.T**	**rs1065853***	**0**	**G**	**T**	**0.08**	**0**	**-14.38**	**6.2E-13**
**19**	**1690**	**3**	**19.45415640.G.A**	**rs445925**	**0**	**G**	**A**	**0.11**	**0**	**-9.84**	**1.7E-08**
19	1690	4	19.45387057.A.G	rs283809	0	A	G	0.05	0	-6.05	2.7E-02
19	1690	4	19.45388241.T.G	rs283810	0	T	G	0.08	0	-3.39	1.3E-01
19	1690	4	19.45389174.T.A	rs283813	0	T	A	0.08	0	-3.44	1.2E-01
19	1690	5	19.45398633.G.C	rs11668327	0	G	C	0.17	0	-5.89	2.2E-04
19	1690	5	19.45406673.G.A	rs10119	0	G	A	0.29	0	2.83	2.9E-02

19	1690	5	19.45408564.A.T	rs449647	0	A	T	0.18	0	-5.23	2.3E-03
19	1690	6	19.45402718.G.C	rs35568738	0	G	C	0.05	0	-1.09	6.7E-01
19	1690	7	19.45394211.C.T	rs76692773	0	C	T	0.09	0	2.02	3.5E-01
19	1690	7	19.45413366.T.C	rs1081106	0	T	C	0.09	0	1.86	4.2E-01
19	1690	8	19.45404691.A.G	rs405697	0	A	G	0.26	0	-1.31	3.2E-01
19	1690	8	19.45414451.T.C	rs439401	0	T	C	0.35	0	-1.05	3.6E-01
19	1690	9	19.45389224.A.G	rs283814	0	A	G	0.06	0	-0.76	7.8E-01
19	1690	10	19.45408628.T.C	rs769446	0	T	C	0.09	0	-2.45	2.7E-01
19	1690	11	19.45413576.C.T	rs75627662	0	C	T	0.20	0	0.12	9.3E-01

* rs1065853 is a proxy of rs7412 (r=0.99); combination of alleles at rs429358 (C or G) and rs7412 (C or G) is known as the APOE-e4 allele which has been associated with LDL-C and increased risk for heart disease.

[1]MLC.Codechange indicates if the variant has been recoded in the LD bin to maximize pairwise positive correlation in the LD bin.

3.3.2 “LDLR locus” (chr19: 10962974- 11280183)

Fig S9. Visualization of the LD blocks within **chr19**:**10962974-11280183 (regions #620-#630)**; produced using the LDblockHeatmap function from the gpart package (for all biallelic SNPs satisfying QC and analyzed in BigLD

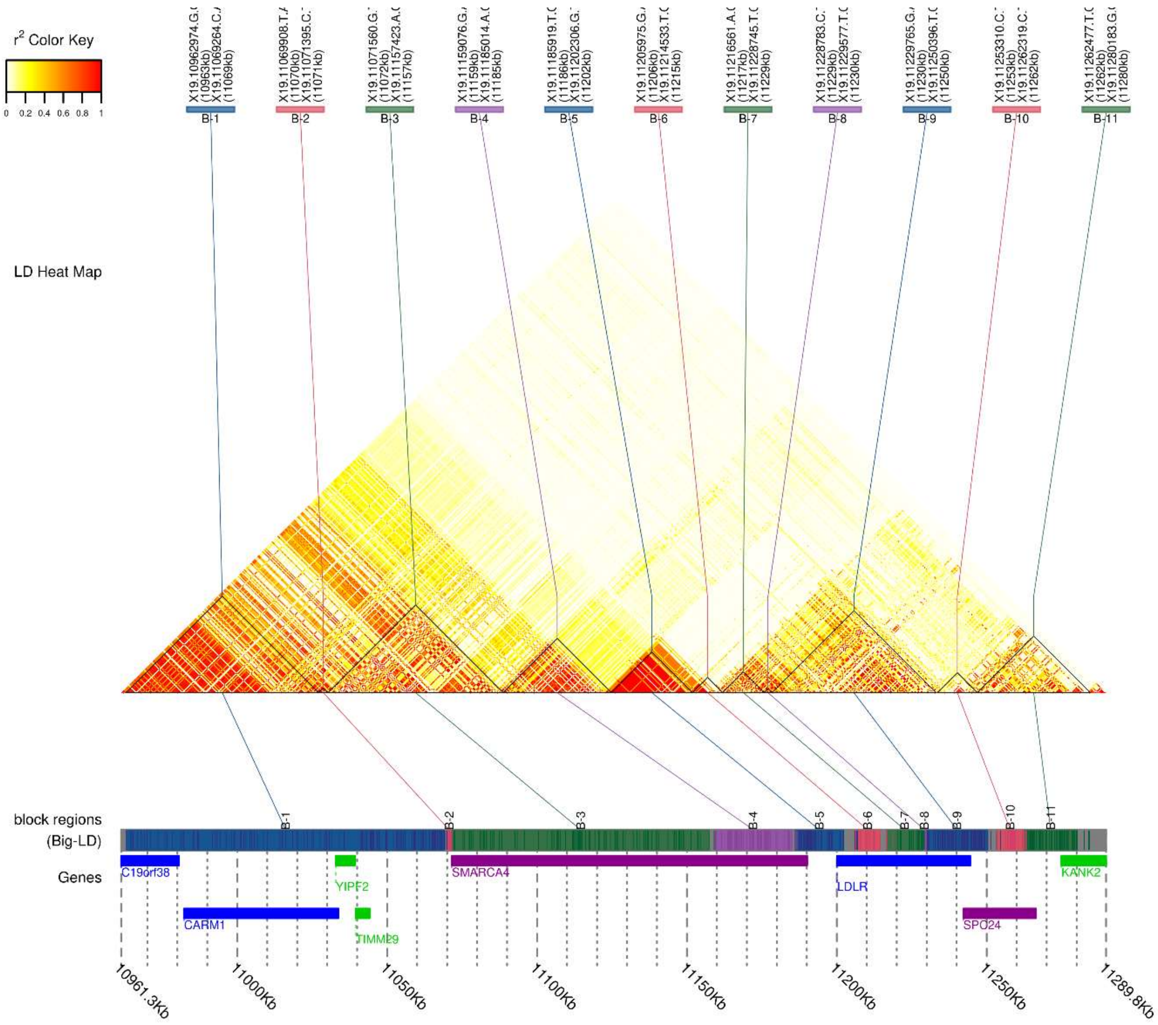

<u>Fig S10.</u> Multi-region association plot for regions #620 to #630 in chr19. The *Y* axis shows the *P*-values (-log10) for four region-level tests and all single-SNP tests. Dashed horizontal lines indicate GW significance thresholds: 5E-8 for single-SNP analysis (blue dotted line) and the genome-wide region-level significance threshold of 5.62E-7 (red dotted line) for region-level. Region numbers appear at the bottom of the plots.

In Panel (A), the physical positions of the regions (and SNPs) are displayed on the X axis, with regions shaded.

In Panel (B), the regions are shown at a fixed, arbitrary size for all regions to facilitate comparisons of the results across them. The boundaries of the regions are indicated by alternating shading. The labels for each region test show the number of SNPs analyzed within each region. To help visualize the relative differences in association signals across tests, lines connect consecutive regions for each region-level test. The number of SNPs analyzed in each region is reported on the corresponding region bands.

(A).

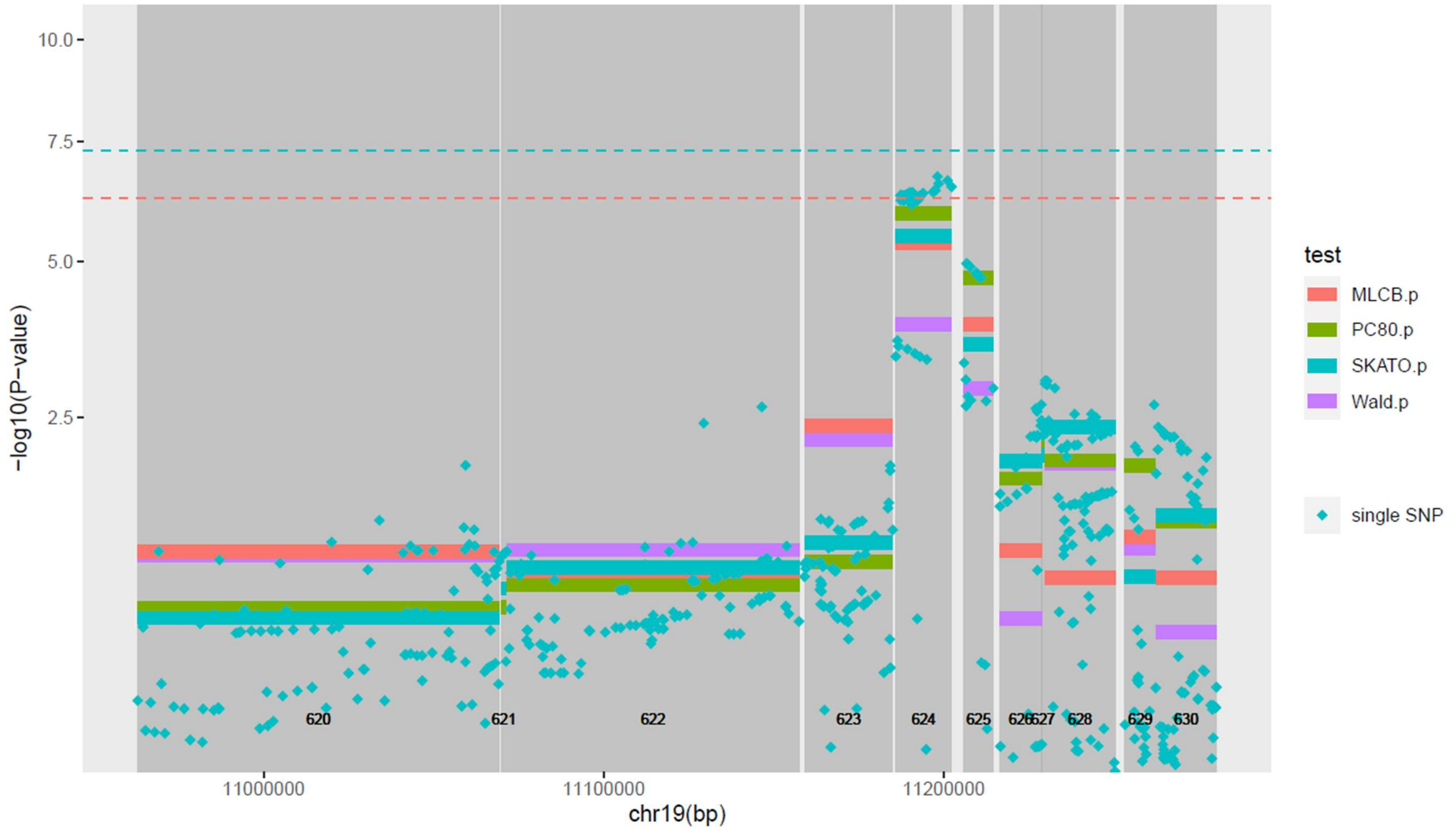

10.0
7.5
5.0
2.5
-log10(P-value)
620
621
622
623
624
625
626
627
628
629
630
11000000
11100000
11200000
chr19(bp)
test
MLCB.p
PC80.p
SKATO.p
Wald.p
single SNP

(B).

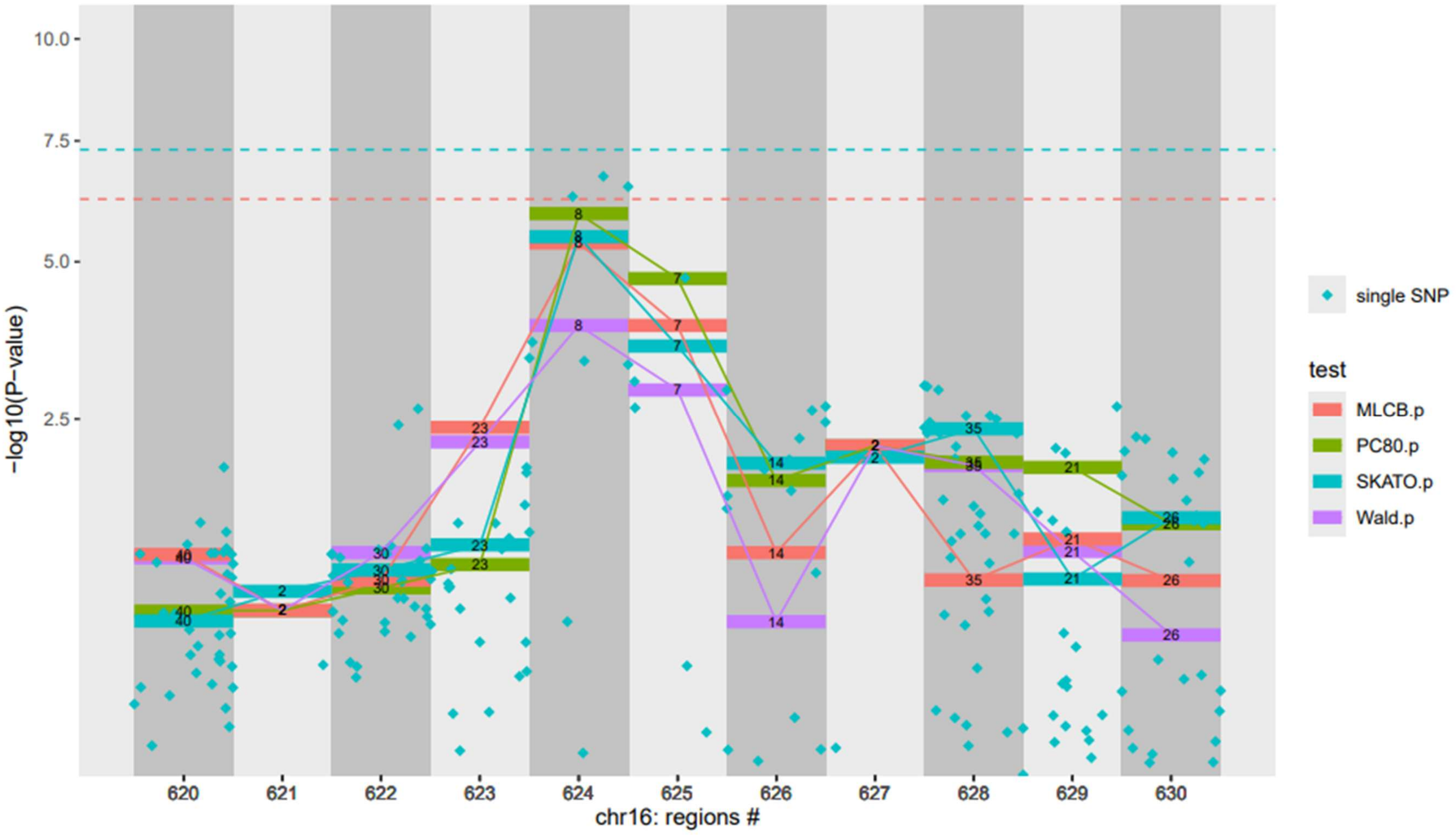

-log10(P-value)
10.0
7.5
5.0
2.5
620
621
622
623
624
625
626
627
628
629
630
chr16: regions #
single SNP
test
MLCB.p
PC80.p
SKATO.p
Wald.p

Fig S11. Visualization of the 3 LD bins in region 624

(A). Heatmap of the correlation matrix in region 624 for the 8 SNPs kept after pruning, with SNPs ordered by LD bin.

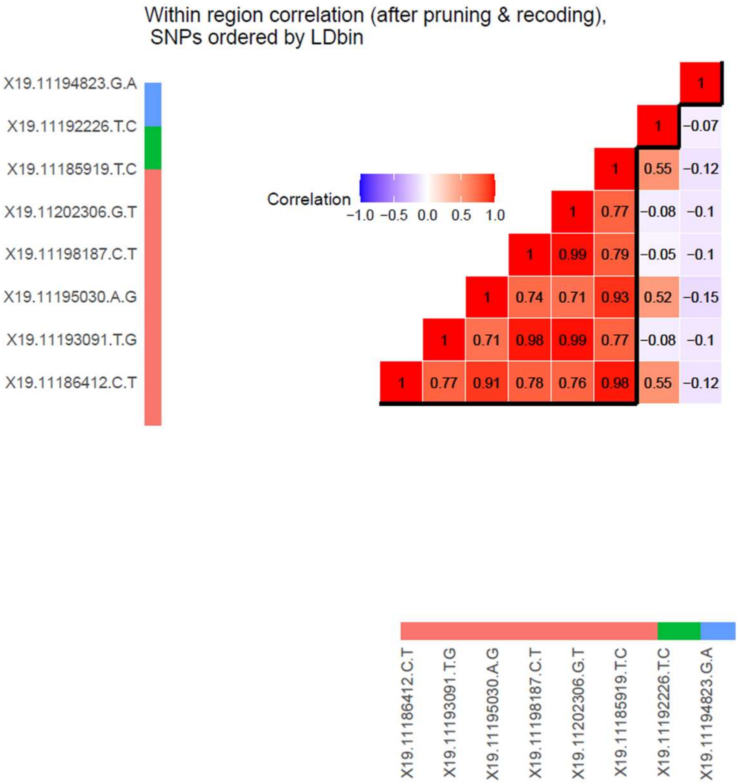

(B). Position of the 8 SNPs (X axis) according to LD bin assignment (Y axis); SNPs shown in grey are assigned to bins but pruned out because of high LD with the remainder kept for the analysis.

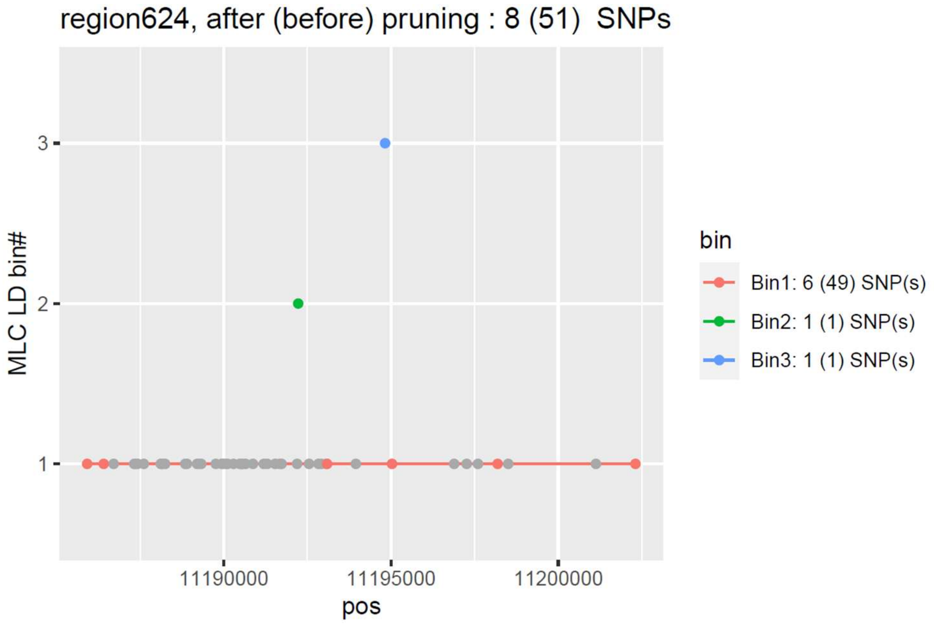

Example: Detailed results for region #624

Region #624 reached the suggestive level of *P*<1E-5 for reduced-*df* region-level tests (MLC, PC80), and variance-like component score test SKATO (**Table S17**). This region includes the SNP rs6511720 consistently reported associated with LDL-C at the genome-wide significance level by multiple previous GWAS (Willer *et al.*, 2008, 2013; Selvaraj *et al.*, 2022). The MLC-based LD Bin-level results show an association for the LD bin 1 (**Tables S18**) which includes 6 SNPs out of which 3 SNPs have single-SNP *P*<5E-7, including rs6511720 (**Fig S11**). Unlike region #1690, MLC, PC80 and SKATO exhibit smaller *P*-values than the Wald region-level test (**Table S17**).

Table S17. Region-level test results for region #624

chr	region	start.bp	end.bp	nSNPs	nSNPs. kept	maxVIF	Wald		MLC		PC80		SKATO.p	ACAT.p[1]
							df	P	df	P	df	P		
19	624	11185919	11202306	51	8	86.8	8	1.3E-04	3	4.4E-06	2	1.2E-06	3.3E-06	2.9E-06

[1]*P*-value based on the Aggregated Cauchy Association Test (ACAT,(Liu *et al.*, 2019)) applied to *P*-values from for Wald, MLC and PC80 region-level tests. ACAT.p is not reported by *regscan but* can be easily computed from the region-level output of *regscan* using the "ACAT" R package (https://github.com/yaowuliu/ACAT).

Table S18. MLC Bin-level results in region #624

chr	region	LD bin	binstart. bfP.bp	binend. bfP.bp	binstart. afP.bp	binend. afP.bp	binsize. bfP	binsize. afP	NSNPs. kept	deltaB	deltaB.se	deltaB. pvalue
**19**	**624**	**1**	**11185919**	**11202306**	**11185919**	**11202306**	**49**	**6**	**6**	**-1.59**	**0.31**	**2.8E-07**
19	624	2	11192226	11192226	11192226	11192226	1	1	1	6.55	2.65	1.4E-02
19	624	3	11194823	11194823	11194823	11194823	1	1	1	-1.27	3.30	7.0E-01

Table S19. Single-SNP results for all 8 variants analyzed region #624 (kept after LD pruning)

chr	region	LD bin	variant	rsID	multiallelic	ref	alt	maf	MLC.codechange[1]	Single-SNP results	
										sglm. beta	sglm. pvalue
19	624	1	19.11185919.T.C	rs10423733	0	T	C	0.17	0	-5.5	4.3E-04
19	624	1	19.11186412.C.T	rs9305019	0	C	T	0.16	0	-5.8	2.4E-04
**19**	**624**	**1**	**19.11193091.T.G**	**rs112552009**	**0**	**T**	**G**	**0.10**	**0**	**-9.2**	**5.2E-07**
19	624	1	19.11195030.A.G	rs11668477	0	A	G	0.19	0	-5.0	4.7E-04
**19**	**624**	**1**	**19.11198187.C.T**	**rs17248720**	**0**	**C**	**T**	**0.11**	**0**	**-9.6**	**1.9E-07**
**19**	**624**	**1**	**19.11202306.G.T**	**rs6511720**	**0**	**G**	**T**	**0.10**	**0**	**-9.3**	**3.2E-07**
19	624	2	19.11192226.T.C	rs7249753	0	T	C	0.06	0	2.7	2.8E-01
19	624	3	19.11194823.G.A	rs11672123	0	G	A	0.05	0	0.3	9.2E-01

[1]MLC.Codechange indicates if the variant has been recoded in the LD bin to maximize pairwise positive correlation in the LD bin.

3.4 List of the participants of the DCCT/EDIC Research Group (as of January 1, 2023)

Study Chairpersons – D.M. Nathan (chair), R. Gubitosi-Klug (vice-chair); *Past*: O. Crofford, B. Zinman; *Deceased*: S. Genuth

Editor, EDIC Publications – D.M. Nathan

Clinical Centers

Case Western Reserve University – *Current*: R. Gubitosi-Klug, L. Mayer, J. Wood, G. Greanoff, D. Miller, M. Novak, S. Pendegast, S. Rath, L. Singerman, D. Weiss, H. Zegarra; *Past*: E. Brown, P. Crawford, M. Palmert, P. Pugsley, J. Quin, S. Smith-Brewer; *Deceased*: W. Dahms, S. Genuth, J. McConnell

Weill Cornell Medical College – *Current*: N.S. Gregory, R. Hanna, R. Chan, S. Kiss, A. Orlin, M. Rubin; *Past*: S. Barron, B. Bosco, D. Brillon, S. Chang, A. Dwoskin, M. Heinemann, L. Jovanovic, M.E. Lackaye, T. Lee, B. Levy, V. Reppucci, M. Richardson; *Deceased*: R. Campbell

Henry Ford Health System – *Current*: A. Bhan, J.K. Jones, D. Kruger, P.A. Edwards, S. Mukhashen; *Past*: E. Angus, A. Galprin, M. McLellan, H. Remtema, A. Thomas; *Deceased*: J.D. Carey, F. Whitehouse

International Diabetes Center – *Current*: R. Bergenstal, S. Dunnigan, M. Johnson, A. Carlson, L. Thomas; *Past*: R. Birk, P. Callahan, G. Castle, R. Cuddihy, M. Franz, D. Freking, L. Gill, J. Gott, K. Gunyou, P. Hollander, D. Kendall, J. Laechelt, S. List, G. Matfin, W. Mestrezat, J. Nelson, B. Olson, N. Rude, M. Spencer; *Deceased*: D. Etzwiler, K. Morgan

Joslin Diabetes Center – *Current*: L.P. Aiello, E. Golden, P. Arrigg, R. Beaser, J. Cavallerano, R. Cavicchi, O. Ganda, O. Hamdy, T. Murtha, D. Schlossman, S. Shah, G. Sharuk, P. Silva, P. Silver, M. Stockman, J. Sun, E. Weimann; *Past*: V. Asuquo, L. Bestourous, A. Jacobson, R. Kirby, L. Rand, J. Rosenzweig, H. Wolpert

Massachusetts General Hospital – *Current*: D.M. Nathan, M.E. Larkin, R. Azevedo, R. Bartholomew, K. Chu, J. Heier, A. Joseph, A. Leong, C. Shah, N. Thangthaeng; *Past*: E. Anderson, H. Bode, S. Brink, M. Cayford, M. Christofi, C. Cornish, D. Cros, S. Crowell, L. Delahanty, A. deManbey, K. Folino, S. Fritz, C. Gauthier-Kelly, J. Godine, L. Gurry, C. Haggan, K. Hansen, F. Leandre, P. Lou, J. Lynch, K. Martin, C. McKitrick, D. Moore, D. Norman, M. Ong, E. Ryan, C. Stevens, C. Taylor, D. Zimbler

Mayo Clinic – *Current*: A. Vella, A. Zipse, A. Barkmeier; *Past*: B. French, M. Haymond, J. Mortenson, J. Pach, R. Rizza, L. Schmidt, W.F. Schwenk, R. Woodwick, G. Ziegler; *Deceased*: R. Colligan, A. Lucas, F.J. Service, B. Zimmerman

Medical University of South Carolina – *Current*: H. Karanchi, L. Spillers, J. Fernandes, K. Hermayer, K. Lee, T. Lyons, M. Nutaitis; *Past*: A. Blevins, M. Bracey, S. Caulder, J. Colwell, S. Elsing, A. Farr, S. Kwon, D. Lee, P. Lindsey, M. Lopes-Virella, L. Luttrell, R. Mayfield, J. Parker, N. Patel, C. Pittman, J. Selby, J. Soule, M. Szpiech, T. Thompson, D. Wood, S. Yacoub-Wasef

Northwestern University – *Current*: A. Wallia, M. Hartmuller, M. El Muayed, M. Gill, A. Lyon, R. Mirza; *Past*: D. Adelman, S. Colson, M. Molitch, B. Schaefer

University of California, San Diego – *Current*: S. Mudaliar, G. Lorenzi, O. Kolterman, M. Goldbaum; *Past*: T. Clark, M. Giotta, I. Grant, K. Jones, R. Lyon, M. Prince, R. Reed, M. Swenson; *Deceased*: G. Friedenberg

University of Iowa – *Current*: W.I. Sivitz, B. Vittetoe; *Past*: M. Bayless, C. Fountain, R. Hoffman, J. Kramer, J. MacIndoe, N. Olson, H. Schrott, L. Snetselaar, T. Weingeist, R. Zeitler

University of Maryland – *Current*: R. Miller, S. Johnsonbaugh; *Past*: M. Carney, D. Counts, T. Donner, J. Gordon, M. Hebdon, R. Hemady, B. Jones, A. Kowarski, R. Liss, S. Mendley, D. Ostrowski, M. Patronas, P. Salemi, S. Steidl

University of Michigan – *Current*: W.H. Herman, R. Pop-Busui, C.L. Martin, P. Lee, J. W. Albers, E.L. Feldman; *Past*: N. Burkhart, D.A. Greene, T. Sandford, M.J. Stevens; *Deceased*: J. Floyd

University of Minnesota – *Current*: A. Bantle, J. Bantle, M. Rhodes, D. Koozekanani, S. Montezuma, J. Terry; *Past*: N. Flaherty, F. Goetz, C. Kwong, L. McKenzie, M. Mech, J. Olson, B. Rogness, T. Strand, J. Terry, R. Warhol, N. Wimmergren

University of Missouri – *Current*: D. Hainsworth, S. Hitt, A. Jarvis; *Past:* D. Goldstein; *Deceased*: J. Giangiacomo

University of New Mexico – *Current*: D.S. Schade, A. Bancroft, R.B. Avery, M.R. Burge, J.E. Chapin, A. Das, L.H. Ketai; *Past*: J.L. Canady, D. Hornbeck, C. Johannes, J. Rich, M.L Schluter

University of Pennsylvania – *Current*: M. Schutta, P.A. Bourne, A. Brucker; *Past*: S. Braunstein, B.J. Maschak-Carey, S. Schwartz; *Deceased*: L. Baker

University of Pittsburgh – *Current*: T. Costacou, F. Toledo, T. Orchard, B.A. Coonrod; *Past*: D. Becker, L. Cimino, B. Doft, D. Finegold, K. Kelly, L. Lobes, D. Rubinstein, N. Silvers, T. Songer, D. Steinberg, L. Steranchak, J.Wesche; *Deceased*: A. Drash

University of South Florida – *Current*: J.I. Malone, A. Morrison, H. Rodriguez, J. O'Brian, P.R. Pavan; *Past*: L. Babbione, M.L. Bernal,T.J. DeClue, N. Grove, D. McMillan, H. Solc, E.A. Tanaka, J. Vaccaro-Kish

University of Tennessee – *Current*: S. Dagogo-Jack, R. Wilson, S. Huddleston; *Past*: M. Bryer-Ash, E. Chaum, A. Iannacone, H. Lambeth, D. Meyer, S. Moser, M.B. Murphy, A. Patel, H. Ricks, S. Schussler, C. Wigley, S. Yoser; *Deceased*: A. Kitabchi

University of Texas Southwestern Medical Center – *Current*: P. Raskin, L. Jordan, B. Shao, YG. He, E. Mendelson, RL. Ufret-Vincenty; *Past*: M. Basco; *Deceased*: S. Cercone, S. Strowig

University of Toronto – *Current*: B.A. Perkins, A. Barnie, N. Bakshi, M. Brent, R. Devenyi, K. Koushan, M. Mandelcorn, D. Olegario, F. Perdikaris; *Past*: D. Daneman, R. Ehrlich, S. Ferguson, A. Gordon, L. Leiter, K. Perlman, S. Rogers, L. Tuason, B. Zinman

University of Washington – *Current*: I. Hirsch, X. Averkiou, I.H. de Boer, L. Olmos de Koo; *Past*: S. Catton, R. Fahlstrom J. Ginsberg, J. Kinyoun, J. Palmer, L. Van Ottingham

University of Western Ontario – *Current*: C. McDonald, M. Driscoll, J. Bylsma, T. Sheidow; *Past*: W. Brown, C. Canny, P. Colby, S. Debrabandere, J. Dupre, J. Harth, I. Hramiak, M. Jenner, J. Mahon, D. Nicolle, N.W. Rodger, T. Smith

Vanderbilt University – *Current*: M. May, T. Marksbury, T. Adkins, A. Agarwal, C. Lovell; *Past*: S. Feman, J. Lipps Hagan, R. Lorenz, R. Ramker; *Deceased*: L. Survant

Washington University, St. Louis – *Current*: A. Brown, N.H. White, E. Hoffman; *Past*: L. Levandoski; *Deceased*: I. Boniuk, J. Santiago

Yale University – *Current*: W. Tamborlane, J. Sherr, P. Gatcomb, K. Stoessel; *Past*: J. Ahern

Albert Einstein – *Past*: J. Brown-Friday, J. Crandall, H. Engel, S. Engel, H. Martinez, M. Phillips, M. Reid, H. Shamoon, J. Sheindlin

Clinical Coordinating Center

Case Western Reserve University – *Current*: R. Gubitosi-Klug, L. Mayer, K. Farrell; *Past*: C. Beck, P. Gaston, M. Palmert, J. Quin, R. Trail; *Deceased*: W. Dahms, S. Genuth

Data Coordinating Center

George Washington University, The Biostatistics Center – *Current*: J. Lachin, I. Bebu, B. Braffett, J. Backlund, M. Bott, L. Diminick, L. El ghormli, X. Gao, S. Ho, D. Kenny, K. Klumpp, M. Lin, V. Trapani; *Past*: K. Anderson, K. Chan, P. Cleary, A. Determan, L. Dews, W. Hsu, P. McGee, H. Pan, B. Petty, D. Rosenberg, B. Rutledge, W. Sun, S. Villavicencio, N. Younes; *Deceased*: C. Williams

National Institute of Diabetes and Digestive and Kidney Disease

National Institute of Diabetes and Digestive and Kidney Disease Program Office – *Current*: E. Leschek; *Past*: C. Cowie, C. Siebert

EDIC Core Central Units

Central Biochemistry Laboratory (University of Minnesota) – *Current*: M. Steffes, A. Karger, J. Seegmiller, V. Arends; *Past*: J. Bucksa, B. Chavers, A. Killeen, M. Nowicki, A. Saenger

Central ECG Reading Unit (Wake Forest School of Medicine) – *Current*: E.Z. Soliman, M. Barr, C. Campbell, S. Hensley, J. Hu, L. Keasler, Y. Li, T. Taylor, Z.M. Zhang; *Past*: Y. Pokharel, R. Prineas

Central Ophthalmologic Reading Unit (University of Wisconsin) – *Current*: B. Blodi, R. Danis, D. Lawrence, H. Wabers; *Past*: M. Burger, M. Davis, J. Dingledine, V. Gama, S. Gangaputra, L. Hubbard, S. Neill, R. Sussman

Central Neuropsychological Reading Unit (NYU Long Island School of Medicine, University of Pittsburgh) – *Current*: A. Jacobson, C. Ryan, D. Saporito; *Past*: B. Burzuk, E. Cupelli, M. Geckle, D. Sandstrom, F. Thoma, T. Williams, T. Woodfill

Supplementary Information 4. Computation time evaluations in an artificial dataset

In this section, we report estimates of the computational runtime (elapsed and CPU times) of *regscan* across a range of study sample sizes and region complexities (number of SNPs analyzed, LD).

4.1 Dataset generation

We simulated an artificial data with genotypes in 16q arm (GRCh38: 46280682p-end, 43Mb) from phased haplotypes for 156,673 biallelic SNPs (MAF>0.01) obtained from high-coverage whole genome sequencing (30x) of 503 unrelated individuals of European ancestry from 1000 Genomes (Byrska-Bishop *et al.*, 2022). We generated a balanced case-control study design of 20,000 cases and 20,000 controls using HAPGEN2(Su *et al.*, 2011) with an assumed disease-generating model based on a log-additive genetic model for the joint effects of five of the most frequent melanoma-associated missense variants in *MC1R* (**Table S20**).

Table S20. Specified odds ratios (ORs) for 5 *MC1R* causal variants

MC1R causal variants	Chr:pos (GRCh38)	MAF[1](%)	Minor/Major alleles	OR[2]
rs1805005	16:89919436	11.2	T/G	1.15
rs2228479	16:89919532	6.9	A/G	1.22
rs1805007	16:89919709	7.2	T/C	1.78
rs1805008	16:89919736	6.2	T/C	1.43
rs885479	16:89919746	7.0	A/G	1.42

[1]MAF in 503 individuals of European ancestry from 1000G.

[2]Specified ORs for each of the 5 causal *MC1R* variants with MAF>0.01 in 1000G/EUR WGS individuals, as reported for melanoma risk(Raimondi *et al.*, 2008), using the minor allele as risk allele.

4.2 Assessment of computational efficiency

To define regions for subsequent region-level analysis we applied BigLD/gpart[30,31] to the 107,292 SNPs of 16q (MAF≥ 0.05) in 20,000 controls. We identified 2,394 regions subsequently analyzed with *regscan* using the default region-level tests implemented and with the default LD pruning in regions and analysis of each region successively (not in parallel). We report a total elapsed time of 355.08 minutes (and CPU time of 7405.07 minutes). Most of these regions had fewer than 50 SNPs analyzed (**Fig S12**).

Fig S12. Distribution of region sizes (no. of SNPs analyzed) among the 2394 regions analyzed in the total sample size.

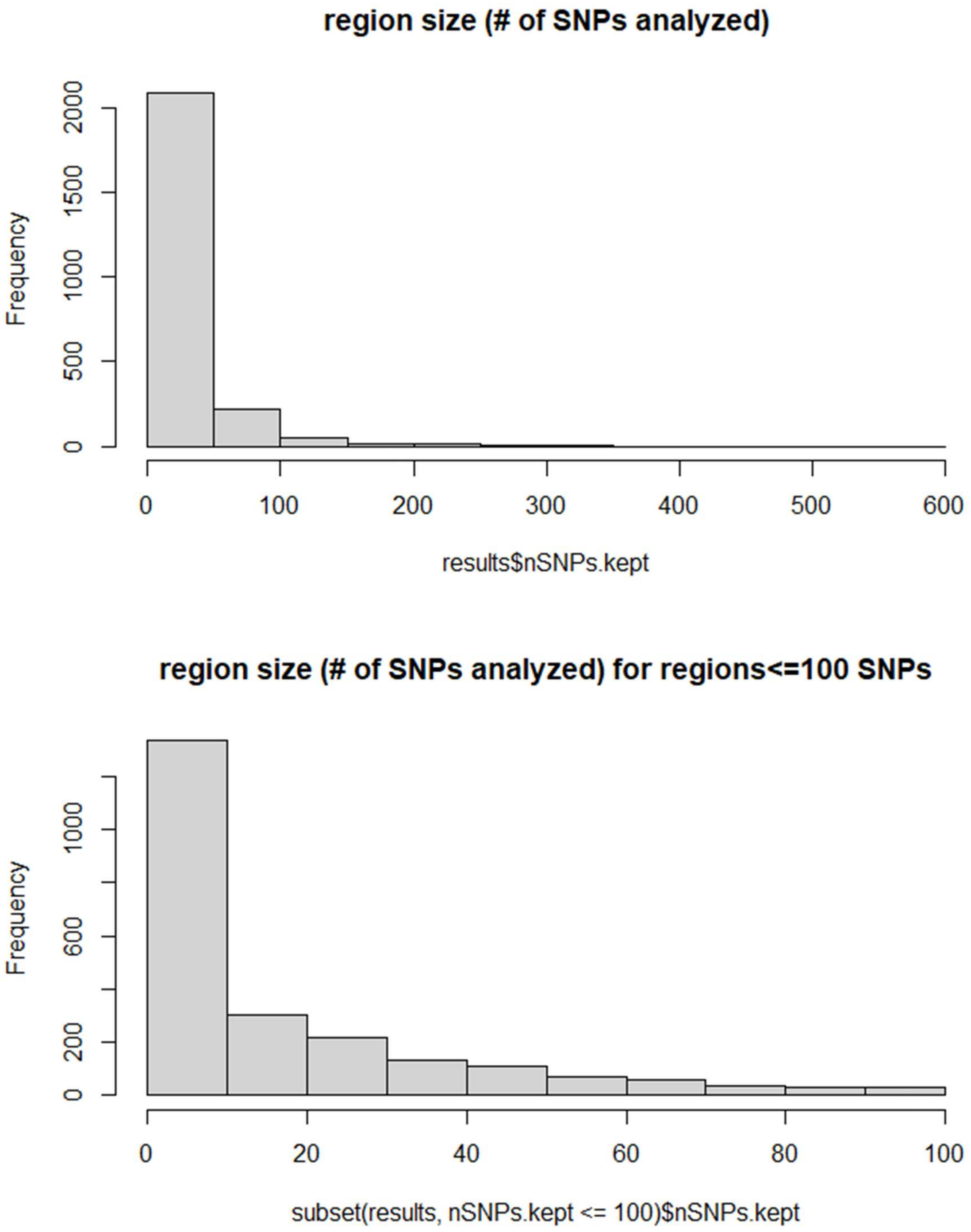

To evaluate the effect of region size and study sample size on the computational time of *regscan*, we selected five regions with varying numbers of SNPs ranging from ~50 to ~600 per region (**Table S21**). Region 5 included multiple causal variants; the other 4 regions did not include any causal variant, but in some cases, some SNPs were in long-range LD with the causal variants in Region 5. We ran *regscan* in the total sample size of 40,000 individuals and in subsamples of 20,000, 10,000, 5,000, and 2,500 individuals. Each sample had a 50/50 case-control ratio, and smaller subsample fully overlap with larger subsamples. For each region, we report the elapsed (**Fig S13**) and CPU (**Fig S14**) times (measured in minutes, transformed on the natural log-scale basis) of *regscan* in each sample size. These computational time assessments were performed on a high-performance computer platform (HPC) using a single node with 40 CPUs and 202 GB RAM.

We point out that regression analyses based on binary outcomes (as illustrated here) are notoriously more computationally intensives than those based on quantitative traits due to slower convergence of the fitting algorithms. For reference, previous evaluations of the computationally efficient software REGENIE for gene-level analysis reported CPU times for binary traits 14.3 times longer than for the same analysis with quantitative traits (see Tables 1 and 2 in (Mbatchou *et al.*, 2021)). A strategy to reduce computational efficiency could be to pre-screen chromosomes or regions based on suggestive signals from single-SNP tests or region-level tests at less stringent significance as obtained with efficient implementations SKAT/SKATO (such as using REGENIE (Mbatchou *et al.*, 2021) or SAIGE-GENE+(Zhou *et al.*, 2022)) and run *regscan* for more comprehensive region-level tests.

Table S21. Regions selected for the computational time assessment of *regscan* (ordered by number of SNPs kept after pruning on LD and removal of the aliases)

Region #	Chr:start-end	Number of SNPs after (before) pruning	Number of MLC LD bins
Region 1	16: 50397624 - 50430199	50 (124)	8
Region 2	16: 80263964 - 80302523	100 (146)	10
Region 3	16: 80924545 - 81096217	201 (632)	23
Region 4	16: 82714946 - 82805368	308 (489)	56
Region 5[1]	16: 89605747 - 89926798	594 (1121)	57

[1]Region5 includes all 5 causal variants specified under the melanoma-generating model.

<u>Fig S13.</u> Evaluation of *regscan* computational efficiency (*Y* axis: Elapsed time in minutes, transformed on the natural log-scale) by study sample size (*X* axis) and number of SNPs per region analyzed. As *regscan* includes steps for variant processing (filtering/clustering/recoding) in addition to the region- and SNP-level analyses, the computational time of *regscan* may also vary depending on different region complexities (e.g. LD structure). For reference on the *Y* axis, 1.5 → log(4.48 minutes); 1 → log(2.72 minutes), 0 → log (1 minute), -1 → log (0.37 minutes, ie 22.2 seconds), -2 → log(0.14 minutes, ie 8.4 seconds), and -3 → log (0.05 minutes, ie 3 seconds). Results for the CPU time are reported in **Fig S14**.

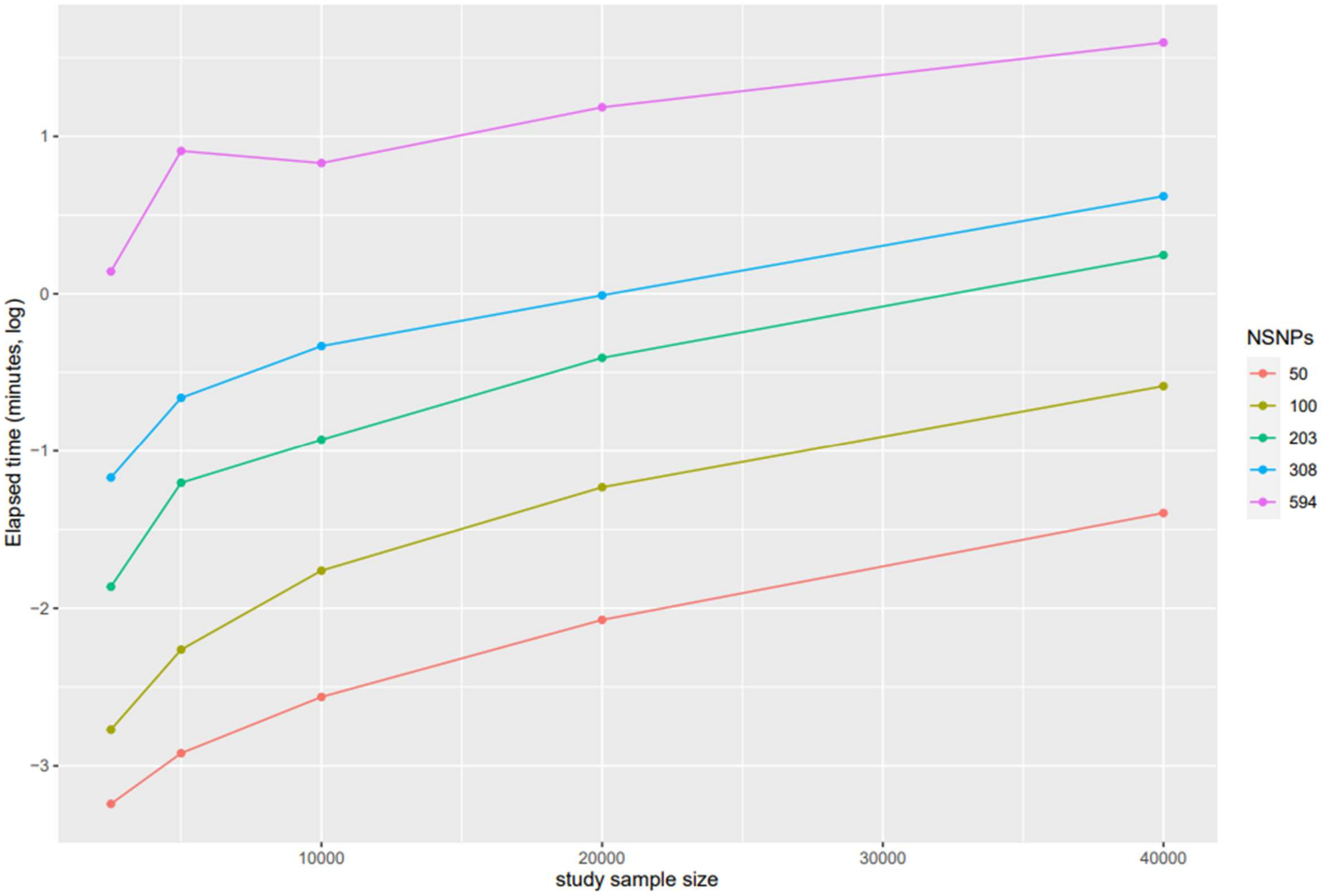

<u>Fig S14.</u> Evaluation of *regscan* computational efficiency (*Y* axis: CPU time in minutes, transformed on the natural log-scale) by study sample size (*X* axis) and number of SNPs per region analyzed. As *regscan* includes steps for variant processing (filtering/clustering/recoding) in addition to the region- and SNP-level analyses, the computational time of *regscan* may also vary depending on different region complexities (e.g. LD structure). For reference on the *Y* axis, 4.5 → log(90 minutes); 4 → log(55 minutes); 3→ log(20 minutes), 2 → log (7.4 minute), 1 → log (2.72 minutes), 0 → log (1 minute), -1 → log (0.37 minutes).

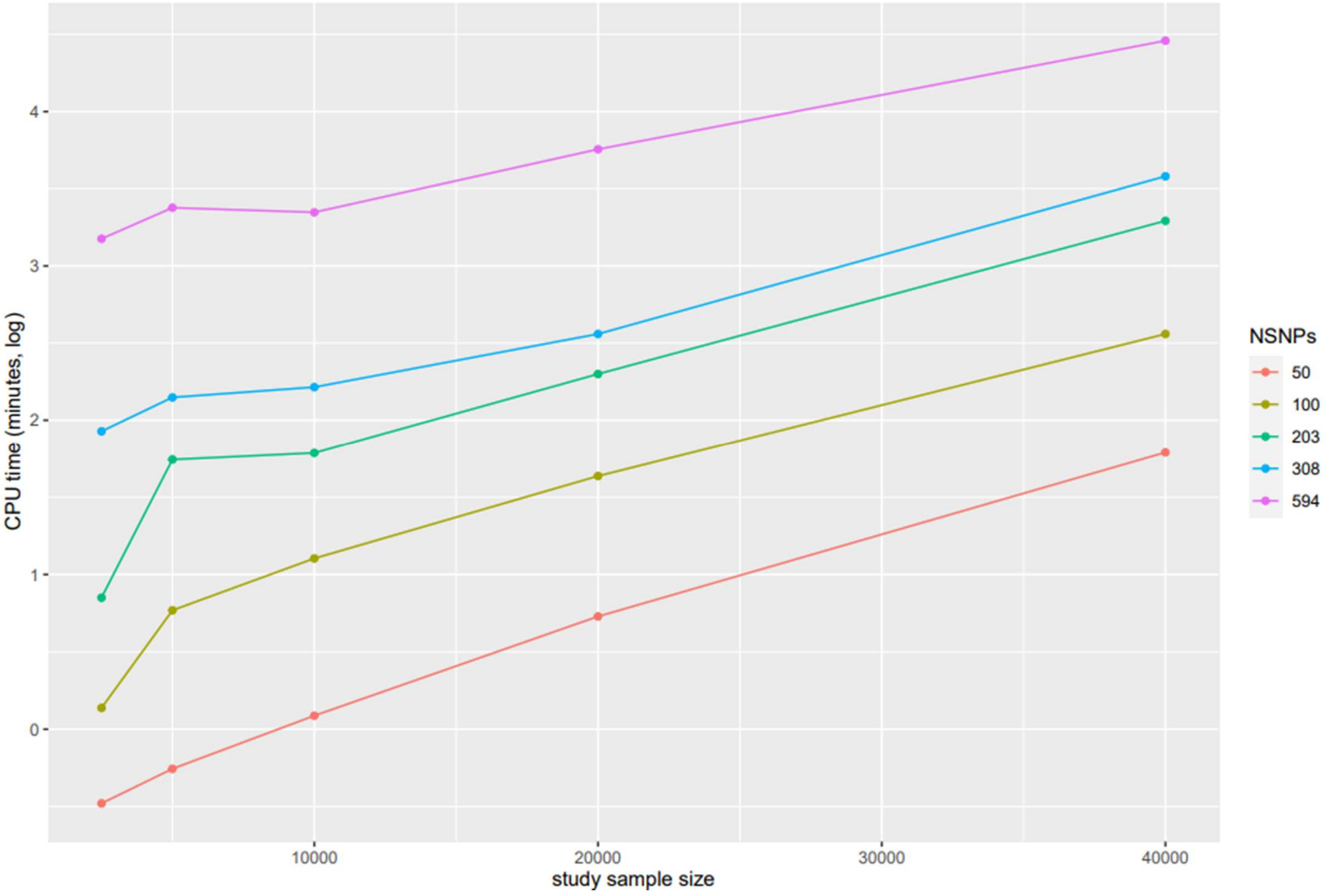

Supplementary Information 5. Comments on potential utility of RegionScan for other applications

5.1 Colocalization analysis for two traits

Although, colocalization analysis based on region-level tests would necessitate methodological developments to address the complexities associated with region-level tests and their dependencies, the *MiamiPlot* function can be used to visualize colocalization for two independent traits within a set of consecutive regions. Once a potential colocalization is indicated, colocalization analysis within a region (or consecutive regions) can be performed using existing colocalization methods such as SharePro (Shared sparse Projection for colocalization analysis, (Zhang *et al.*, 2024)) which groups highly correlated variants into effect groups and calculates colocalization probabilities at the group levels, employing an efficient variational inference algorithm. Summary statistics from single-SNP analysis - required by SharePro - can be extracted from the variant-level output of *regscan*, and the LD matrix between the variants can be computed separately using, for example, PLINK.

5.2 Combination of region-level tests obtained for common and rare variants

Although RegionScan is not limited to common variants (CVs) - the minimum allele frequency (MAF) threshold can be adjusted using the *MAFcut* option - we acknowledge that it is primarily designed for CVs – and therefore specific challenges arising from rare variant (RV) analysis needs to be addressed in practice (eg. computational efficiency, convergence issues in regression models). It may not be as scalable as software specifically tailored for RV and whole genome sequenced data, such as REGENIE (Mbatchou *et al.*, 2021) or SAIGE-GENE (Zhou *et al.*, 2020, 2022), which also include filtering options for RVs based on functional annotation categories in addition to efficient implementations of region-level tests designed for RVs (burden tests, SKAT/SKATO).

In addition, region definition for RV analysis presents unique challenges due to their distinct LD patterns compared to CVs. Conventional region-definitions for RVs often rely most commonly on coding regions and sometimes on arbitrary-sized fixed windows (for example, (Pathan *et al.*, 2024; Karczewski *et al.*, 2022)) which may pose challenges in the interpretation of the results. In our opinion, LD-partitioning based on D' available in the BigLD/gpart package is a more flexible and appropriate framework for defining regions for RVs. Indeed, BigLD includes options RVs can be assigned to LD-based regions constructed in CVs, using specific rules based on the RV positions relative to the blocks and LD with other variants within the blocks; this approach is also implemented in BigLD function (options: appendRare=FALSE, and LD= "Dprime") of the gpart R package. A potential strategy for analyzing CVs + RVs could involve BigLD-region definition based on CVs + RVs (as mentioned above), followed by: *(i)* region-level analysis for CVs using

RegionScan, *(ii)* region-level analysis for RVs using REGENIE or SAIGE-GENE, and *(iii)* integrating the results of both analyses using for example the Cauchy combination test (ACAT, (Liu *et al.*, 2019)).

References

Byrska-Bishop,M. *et al.* (2022) High-coverage whole-genome sequencing of the expanded 1000 Genomes Project cohort including 602 trios. *Cell*, **185**, 3426-3440.e19.

CRAN: Package brglm2.

Das,S. *et al.* (2016) Next-generation genotype imputation service and methods. *Nat Genet*, **48**, 1284–1287.

Gao,X. *et al.* (2008) A multiple testing correction method for genetic association studies using correlated single nucleotide polymorphisms. *Genet Epidemiol*, **32**, 361–369.

Gauderman,W.J. *et al.* (2007) Testing association between disease and multiple SNPs in a candidate gene. *Genet Epidemiol*, **31**, 383–395.

Graham,S.E. *et al.* (2021) The power of genetic diversity in genome-wide association studies of lipids. *Nature 2021 600:7890*, **600**, 675–679.

Karczewski,K.J. *et al.* (2022) Systematic single-variant and gene-based association testing of thousands of phenotypes in 394,841 UK Biobank exomes. *Cell genomics*, **2**.

Kim,S.A. *et al.* (2018) A new haplotype block detection method for dense genome sequencing data based on interval graph modeling of clusters of highly correlated SNPs. *Bioinformatics*, **34**, 388–397.

Kim,S.A. *et al.* (2019) gpart: human genome partitioning and visualization of high-density SNP data by identifying haplotype blocks. *Bioinformatics*, **35**, 4419–4421.

Kosmidis,I. *et al.* (2020) Mean and median bias reduction in generalized linear models. *Stat Comput*, **30**, 43–59.

Kosmidis,I. and Firth,D. (2021) Jeffreys-prior penalty, finiteness and shrinkage in binomial-response generalized linear models. *Biometrika*, **108**, 71–82.

Kwee,L.C. *et al.* (2008) A Powerful and Flexible Multilocus Association Test for Quantitative Traits. *The American Journal of Human Genetics*, **82**, 386–397.

Lee,S., Emond,Mary J., *et al.* (2012) Optimal Unified Approach for Rare-Variant Association Testing with Application to Small-Sample Case-Control Whole-Exome Sequencing Studies. *The American Journal of Human Genetics*, **91**, 224–237.

Lee,S., Emond,Mary J, *et al.* (2012) Optimal Unified Approach for Rare-Variant Association Testing with Application to Small-Sample Case-Control Whole-Exome Sequencing Studies. 224–237.

Li,M.X. *et al.* (2011) GATES: A rapid and powerful gene-based association test using extended Simes procedure. *Am J Hum Genet*, **88**, 283–293.

LI,Q.H. and LAGAKOS,S.W. (2006) On the Relationship between Directional and Omnibus Statistical Tests. *Scandinavian Journal of Statistics*, **33**, 239–246.

Liu,Y. *et al.* (2019) ACAT: A Fast and Powerful p Value Combination Method for Rare-Variant Analysis in Sequencing Studies. *Am J Hum Genet*, **104**, 410–421.

Marchini,J. and Howie,B. (2010) Genotype imputation for genome-wide association studies. *Nat Rev Genet*, **11**, 499–511.

Mbatchou,J. *et al.* (2021) Computationally efficient whole-genome regression for quantitative and binary traits. *Nat Genet*, **53**, 1097–1103.

Package 'SKAT' Title SNP-Set (Sequence) Kernel Association Test (2023).

Paterson,A.D. *et al.* (2010) A genome-wide association study identifies a novel major locus for glycemic control in type 1 diabetes, as measured by both A1C and glucose. *Diabetes*, **59**, 539–549.

Pathan,N. *et al.* (2024) A method to estimate the contribution of rare coding variants to complex trait heritability. *Nature Communications 2024 15:1*, **15**, 1–16.

Pocock,S.J. *et al.* (1987) The Analysis of Multiple Endpoints in Clinical Trials. *Biometrics*, **43**, 487.

Raimondi,S. *et al.* (2008) MC1R variants, melanoma and red hair color phenotype: A meta-analysis. *Int J Cancer*, **122**, 2753–2760.

Raina,P. *et al.* (2019) Cohort Profile: The Canadian Longitudinal Study on Aging (CLSA). *Int J Epidemiol*, **48**, 1752-1753J.

Rasmussen-Torvik,L.J. *et al.* (2012) High Density GWAS for LDL Cholesterol in African Americans Using Electronic Medical Records Reveals a Strong Protective Variant in *APOE*. *Clin Transl Sci*, **5**, 394–399.

Roshandel,D. *et al.* (2018) Meta-genome-wide association studies identify a locus on chromosome 1 and multiple variants in the MHC region for serum C-peptide in type 1 diabetes. *Diabetologia*, **61**, 1098–1111.

Sanna,S. *et al.* (2011) Fine Mapping of Five Loci Associated with Low-Density Lipoprotein Cholesterol Detects Variants That Double the Explained Heritability. *PLoS Genet*, **7**, e1002198.

Selvaraj,M.S. *et al.* (2022) Whole genome sequence analysis of blood lipid levels in >66,000 individuals. *Nat Commun*, **13**, 5995.

Shamoon,H. *et al.* (1999) Epidemiology of Diabetes Interventions and Complications (EDIC). Design, implementation, and preliminary results of a long-term follow-up of the Diabetes Control and Complications Trial cohort. *Diabetes Care*, **22**, 99–111.

SKAT Package.

Stram,D.O. *et al.* (1988) Analysis of repeated ordered categorical outcomes with possibly missing observations and time-dependent covariates. *J Am Stat Assoc*, **83**, 631–637.

Su,Z. *et al.* (2011) HAPGEN2: simulation of multiple disease SNPs. *Bioinformatics*, **27**, 2304–2305.

The 1000 Genomes Project Consortium *et al.* (2015) A global reference for human genetic variation. *Nature*, **526**, 68–74.

The Diabetes Control and Complications Trial (DCCT). Design and methodologic considerations for the feasibility phase. The DCCT Research Group. (1986) *Diabetes*, **35**, 530–45.

Willer,C.J. *et al.* (2013) Discovery and refinement of loci associated with lipid levels. *Nature Genetics 2013 45:11*, **45**, 1274–1283.

Willer,C.J. *et al.* (2008) Newly identified loci that influence lipid concentrations and risk of coronary artery disease. *Nat Genet*, **40**, 161–169.

Yoo,Y.J. *et al.* (2015) Clique-Based Clustering of Correlated SNPs in a Gene Can Improve Performance of Gene-Based Multi-Bin Linear Combination Test. *Biomed Res Int*, **2015**, 1–11.

Yoo,Y.J. *et al.* (2017) Multiple linear combination (MLC) regression tests for common variants adapted to linkage disequilibrium structure. *Genet Epidemiol*, **41**, 108–121.

Zhang,W. *et al.* (2024) SharePro: an accurate and efficient genetic colocalization method accounting for multiple causal signals. *Bioinformatics*, **40**.

Zhou,W. *et al.* (2022) SAIGE-GENE+ improves the efficiency and accuracy of set-based rare variant association tests. *Nature Genetics 2022 54:10*, **54**, 1466–1469.

Zhou,W. *et al.* (2020) Scalable generalized linear mixed model for region-based association tests in large biobanks and cohorts. *Nature Genetics 2020 52:6*, **52**, 634–639.

Annex. Vignette of RegionScan

(see next pages)

Get started with `RegionScan`

Myriam Brossard

December 1st, 2024

`RegionScan` is designed for scalable genome-wide association testing of both region-level multiple-variant and single-variant statistics, with visualization of the results. For detection of association under various regional architectures, it implements three classes of state-of-the-art region-level tests, including multiple-variant linear/logistic regression (with and without dimension reduction), variance-component score tests, and region-level minP tests. `RegionScan` also supports the analysis of multi-allelic variants and unbalanced binary phenotypes and is compatible with widely used variant call format (VCF) files for both genotyped and imputed variants. Association testing leverages linkage disequilibrium (LD) structure in pre-defined regions, for example, LD-adaptive regions obtained by genomic partitioning, and accommodates parallel processing to improve computational and memory efficiency. Detailed outputs (with allele frequencies, variant-LD bin assignment, single/joint variant effect estimates and region-level results) and utility functions are provided to assist comparison, visualization, and interpretation of results. Thus, `RegionScan` analysis offers valuable insights into region-level genetic architecture which supports a wide range of potential applications.

In this vignette, we illustrate basic usage of `RegionScan` functions applied to a reproducible example of dataset provided with the package. The list of main functions implemented in `RegionScan`, options as well as description of the outputs of main function regscan are in Annex of this vignette.

Installation

```
library(devtools)
install_github("brossardMyriam/RegionScan")
library(RegionScan)
```

Basic usage of `regscan`

Example dataset

This example dataset is based on 436 biallelic SNPs (MAF>0.05) genotyped in chr16:46382489-47684754 in 40,000 individuals simulated from high coverage whole genome sequenced 1000G European ancestry haplotypes using HAPGEN2 software. For this illustration, we used 20 consecutive regions identified by LD partitioning of chr16 using the BigLD algorithm (https://pubmed.ncbi.nlm.nih.gov/29028986/) implemented in R package *gpart* (https://academic.oup.com/bioinformatics/article/35/21/4419/5487391). However, *RegionScan* accepts any type of user-defined region boundaries (e.g. gene start/end positions etc). We simulated a quantitative trait (*sim_QT* in the `phenocov` input) and a binary trait (*sim_bin*) ; sim_QT was generated under a linear regression model, assuming joint effects of two causal SNPs in region 8 ("chr16.46880510.A.G", "chr16.46889594.G.A"); while *sim_bin* was generated by dichotomizing *sim_QT* (see data_simulation.R for details).

In this vignette, we illustrate how to run the main function *regscan* for region-level and single-SNP analysis and how to visualize the results.

Main inputs

Three main inputs required by regscan

Input 1: REGIONinfo This dataframe must include at least `chr`, `start.bp`, `end.bp`, `region`; regions start and end positions.

```
head(REGIONinfo)
#>   chr start.index end.index          start.rsID           end.rsID start.bp
#> 1  16           4        14 chr16.46382489.T.A chr16.46401970.C.T 46382489
#> 2  16          16       175 chr16.46402078.C.G chr16.46490675.G.A 46402078
#> 3  16         187       230 chr16.46505480.G.A chr16.46568409.T.A 46505480
#> 4  16         235       271 chr16.46568907.C.T chr16.46614446.G.A 46568907
#> 5  16         289       293 chr16.46633030.A.T chr16.46641133.G.A 46633030
#> 6  16         298       439 chr16.46651722.T.A chr16.46793612.G.A 46651722
#>     end.bp region
#> 1 46401970      1
#> 2 46490675      2
#> 3 46568409      3
#> 4 46614446      4
#> 5 46641133      5
#> 6 46793612      6
```

Input 2: geno This dataframe must include genotypes (columns) of the individuals (rows) ; as illustrated below for 4 genetic variants. The individuals must be in the same order as the individuals in input `phenocov`

```
head(geno[,1:4])
#>   chr16.46382489.T.A chr16.46401970.C.T chr16.46402078.C.G chr16.46402735.C.T
#> 1                  0                  0                  0                  0
#> 2                  0                  1                  0                  1
#> 3                  1                  1                  0                  1
#> 4                  0                  0                  1                  0
#> 5                  0                  0                  0                  0
#> 6                  0                  0                  1                  0
```

Input 3: SNPinfo This dataframe includes the variant positions and information for the variants in `geno` input ; all the following columns are required

```
head(SNPinfo)
#>    chr       bp            variant multiallelic ref alt      maf
#> 4   16 46382489 chr16.46382489.T.A            0   T   A 0.132125
#> 14  16 46401970 chr16.46401970.C.T            0   C   T 0.148238
#> 16  16 46402078 chr16.46402078.C.G            0   C   G 0.481212
#> 19  16 46402735 chr16.46402735.C.T            0   C   T 0.147688
#> 35  16 46404976 chr16.46404976.G.C            0   G   C 0.054150
#> 38  16 46405393 chr16.46405393.C.A            0   C   A 0.110875
```

Input 4: phenocov This dataframe must includes phenotypes and covariates for all the individuals of input `geno`. The individuals must be in the same order as in input 'geno.

```
head(phenocov)
#>   ID     sim_QT sim_bin
#> 1  1 -0.8418605       0
#> 2  2  2.5931863       1
#> 3  3  4.9720520       1
#> 4  4  3.4298926       1
#> 5  5  3.5351976       1
#> 6  6  3.3847384       1
```

Single & Region-level analysis

Example of run of `regscan` main function for region & single-SNP level analysis with the continuous outcome `sim_QT` .

```
results<-regscan(phenocov = phenocov, pheno="sim_QT",  REGIONinfo=REGIONinfo,
                 geno_type="D", pheno_type="C", data = geno,  SNPinfo = SNPinfo )
```

Main Outputs

The main output of `regscan` function is a list including the region-level, Bin-level, variant-level outputs and filtered variant lists; and optionally an additional output (sinleSNPall) with single-SNP results for all the variants analyzed (including filtered variants in region-level analysis).

Output 1: Region-level results This is the main output which includes results from all the region-level test implemented in RegionScan for all regions from REGIONinfo.

```
head(results$regionout)
#>   chr region start.bp   end.bp nSNPs nSNPs.kept           maxVIF
#> 1  16      1 46382489 46401970     2          2 7.63118409358028
#> 2  16      2 46402078 46490675    33         29 89.3068478742558
#> 3  16      3 46505480 46568409    16          9  36.158052040736
#> 4  16      4 46568907 46614446    11          7 38.4478358507763
#> 5  16      5 46633030 46641133     2          1             <NA>
#> 6  16      6 46651722 46793612    79         35 180.756972532698
#>               Wald Wald.df               Wald.p             PC80 PC80.df
#> 1 13.2183097619898       2  0.00134797086396498 11.4158641131233       1
#> 2 126.944786126531      29 3.21618445117108e-14 84.3542012194867       5
#> 3 58.5717069136177       9 2.52433174409188e-09 28.5345978528676       3
#> 4 102.804756484661       7 2.84007861980665e-19 95.8824234218916       3
#> 5 11.1273685632092       1 0.000850631832676347 11.1273685632029       1
#> 6 136.694141275958      35 5.95571393547455e-14 84.6911418092466       4
#>                   PC80.p             MLCB MLCB.df               MLCB.p
#> 1 0.000728196198525501 11.4552092034544       1 0.000712938624286533
#> 2 1.02813148101739e-16 87.1426177690514       9 6.08481865525033e-15
#> 3 2.80483261162339e-06 36.2666724304519       4 2.55025052900061e-07
#> 4 1.19307154961529e-20 97.8991119543123       4 2.75428945545249e-20
#> 5 0.000850631832679229 11.1273685632092       1 0.000850631832676347
#> 6 1.76399337813415e-17 99.3399418631948       8 5.82398514579283e-18
#>                LCB LCB.df                LCB.p               SKAT.p
#> 1 11.4552092034544      1 0.000712938624286533  0.00104947556871415
#> 2 34.9763378622398      1 3.33736410188684e-09 9.74634425131443e-15
#> 3 7.08311103881546      1  0.00778137995814597    0.215689691960729
#> 4 48.6354481085035      1  3.0824701317004e-12 3.03026009628285e-17
#> 5 11.1273685632092      1 0.000850631832676347  0.00085192453877572
#> 6 22.1885012754748      1 2.47149570476665e-06 8.42588081355409e-18
#>                  SKATO.p              simes.p simpleM.df            simpleM.p
#> 1 0.000895448518125508 0.000359829455696319          2 0.000719529434155541
#> 2   6.8224409759201e-14 1.19993233644567e-13        22 6.81454892514921e-13
#> 3    0.0534490049341949 0.000171294066412414         8 0.000318336642977646
#> 4 2.12118206739799e-16 6.22561765526543e-17          6                    0
#> 5  0.00085192453877572 0.000851405665034688          1 0.000851405665034688
#> 6 5.89811656948778e-17 1.08416039850315e-16        22 2.44249065417534e-15
#>                  GATES.p        single_Wald.p
#> 1 0.000444211392039609 0.000359829455696319
```

```
#> 2  4.5042899698509e-13 3.10037494646998e-14
#> 3 0.000249844769560631 3.97976234088524e-05
#> 4 2.80476976663764e-16 5.45667595824671e-17
#> 5 0.000851405665034688 0.000851405665034688
#> 6 1.81250162120005e-15 1.08416039850315e-16
```

Output 2: Bin-level results This output includes the bin-level association results (deltaB, deltaB.se, deltaB.pvalue) for each LD bin identified in the regions for the MLC test. It also includes the bin sizes (ie. number of SNPs in each LD bin before and after LD-based pruning. The bin-level results can help to investigate some MLC region-level test results.

```
head(results$binout)
#>   chr region start.bp   end.bp binstart.bfP.bp binend.bfP.bp binstart.afP.bp
#> 1  16      1 46382489 46401970        46382489      46401970        46382489
#> 2  16      2 46402078 46490675        46402735      46466492        46402735
#> 3  16      2 46402078 46490675        46402078      46490675        46402078
#> 4  16      2 46402078 46490675        46405393      46467231        46405393
#> 5  16      2 46402078 46490675        46411236      46461260        46411236
#> 6  16      2 46402078 46490675        46459836      46460534        46459836
#>   binend.afP.bp binsize.bfP binsize.afP bin NSNPs.kept               deltaB
#> 1      46401970           2           2   1          2  -0.0263044227412342
#> 2      46466492           9           6   1          6  -0.0024261415198229
#> 3      46490675           6           6   2          6  0.00857041275832189
#> 4      46467231           6           5   3          5 -0.00192016759715904
#> 5      46461260           6           6   4          6 -0.00109835921410416
#> 6      46460534           2           2   5          2   0.0642642715636263
#>              deltaB.se        deltaB.pvalue
#> 1 0.00777190105679396 0.000712938624286533
#> 2 0.00410934586819968    0.554925166125585
#> 3 0.00380060380613128   0.0241324836127966
#> 4 0.00612698369034169    0.753980391088406
#> 5 0.00435899531386413    0.801060144650654
#> 6  0.0116418431625167 3.38784749048239e-08
```

Output 3: variant-level results This output provides detailed results at the variant-level for all variants kept for the region-level analysis. The variant-specific results from multiple regression at the region level and single-SNP regression models are reported. Investigation of this output can help to investigate the region-level results (particularly for the MLC region-level test).

```
head(results$snpout)
#>   chr region start.bp   end.bp bin       bp multiallelic ref alt       maf
#> 1  16      1 46382489 46401970   1 46382489            0   T   A 0.1321250
#> 2  16      1 46382489 46401970   1 46401970            0   C   T 0.1482375
#> 3  16      2 46402078 46490675   1 46402735            0   C   T 0.1476875
#> 4  16      2 46402078 46490675   1 46407733            0   G   A 0.1472250
#> 5  16      2 46402078 46490675   1 46424244            0   T   C 0.1471250
#> 6  16      2 46402078 46490675   1 46452260            0   C   T 0.1516500
#>   MLC.codechange LC.codechange            variant           sglm.beta
#> 1              0             0 chr16.46382489.T.A -0.0480730798258817
#> 2              0             0 chr16.46401970.C.T -0.0532834375150429
#> 3              0             1 chr16.46402735.C.T -0.0524756816395952
#> 4              0             1 chr16.46407733.G.A -0.0570326612654028
#> 5              0             1 chr16.46424244.T.C  -0.051518678296245
#> 6              0             1 chr16.46452260.C.T -0.0531965054431854
```

```
#>               sglm.se          sglm.pvalue  mglm.vif   mglm.beta    mglm.se
#> 1  0.015640279358937  0.00211584432310794  7.631184  0.03012700 0.04320415
#> 2 0.0149327616684696 0.000359829455696319  7.631184 -0.08009788 0.04125142
#> 3 0.0149490636258694 0.000448095479251166 45.566160  0.07890890 0.10080117
#> 4 0.0149628641470136 0.000138269175752732 69.116155 -0.18959396 0.12426444
#> 5 0.0149762180348197 0.000582237338420528 41.839892  0.12426043 0.09676653
#> 6 0.0147921645682756 0.000323210773092559 89.306848 -0.28737353 0.13963935
#>   mglm.pvalue
#> 1  0.48560835
#> 2  0.05218043
#> 3  0.43373903
#> 4  0.12708509
#> 5  0.19910525
#> 6  0.03959952
```

Output 4: variants excluded This output lists all the variants excluded within each region and the reasons of exclusion (MAF, LD pruning etc); the LD bin information is also reported.

```
head(results$filterout)
#>   chr region    start      end       bp            variant bin reason
#> 1  16      2 46402078 46490675 46418341 chr16.46418341.G.A   1   rcut
#> 2  16      2 46402078 46490675 46435570 chr16.46435570.C.T   1   rcut
#> 3  16      2 46402078 46490675 46439309 chr16.46439309.G.A   1   rcut
#> 4  16      2 46402078 46490675 46444013 chr16.46444013.T.C   3   rcut
#> 5  16      3 46505480 46568409 46539340 chr16.46539340.T.C   1   rcut
#> 6  16      3 46505480 46568409 46539341 chr16.46539341.G.A   1   rcut
```

Visualisation of the results

Locus plot Illustration of the LocusPlot function which plots the region-level results in a set of consecutive regions. The plot is directly saved as a pdf in the local directory.

```
LocusPlot(chr=16,pheno="sim_QT",regscanout=results,regionlist=c(1:15),outname="LocusPlot_region1_15",
          region_tests=c("Wald.p","PC80.p","MLCB.p","SKATO.p"))
```

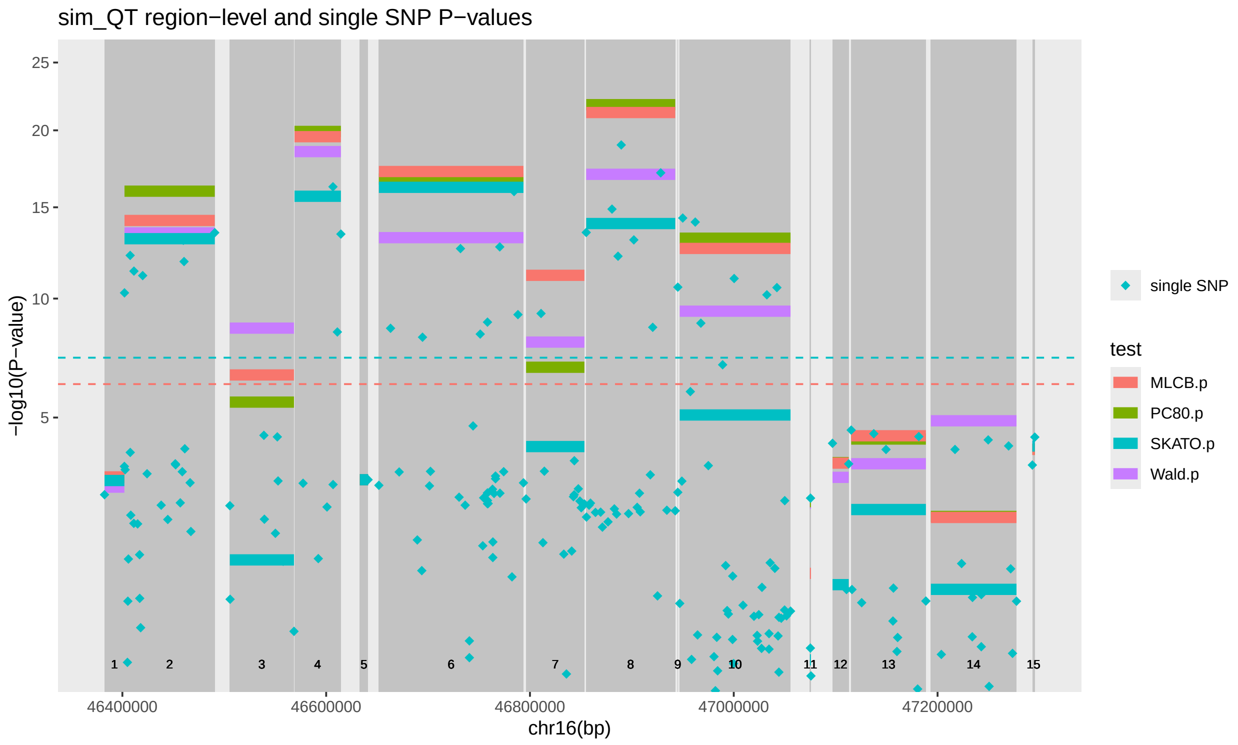

Figure 1: Locus plot of consecutive regions

LD heatmaps The following function produces LD heatmaps for a specified region (here region "8") to visualize the correlation structure within region, before and after pruning, as well as the dependencies between the LD bins. Plots are saved as pdf files in the local directory.

```
regscan(phenocov = phenocov, pheno="sim_QT",  REGIONinfo=REGIONinfo,
        geno_type="D", pheno_type="C", data = geno,  SNPinfo = SNPinfo,
        MLCheatmap = TRUE, regionlist ="8" )
```

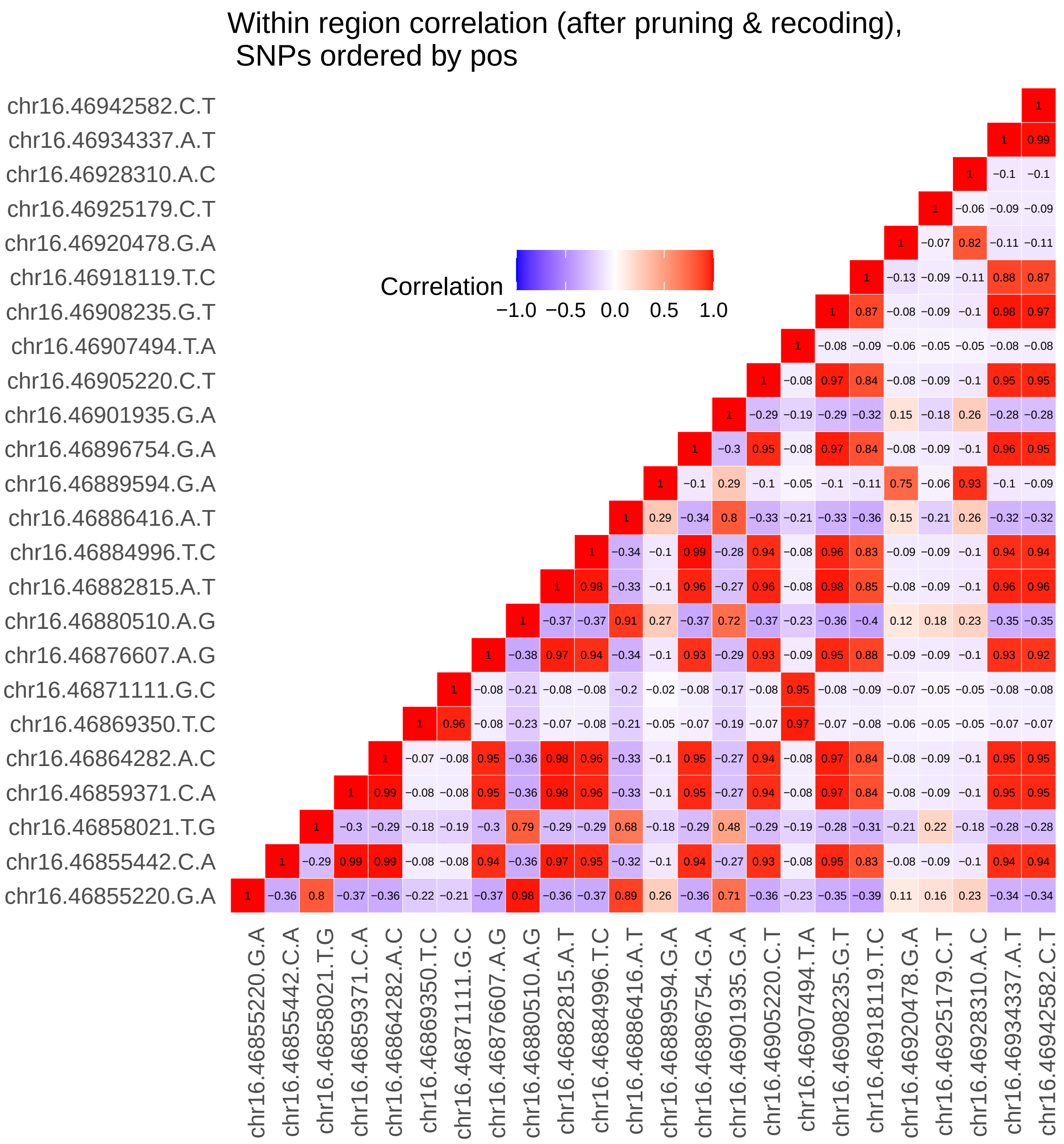

Figure 2: Example 1 of LD heatmap plot for region 8 (after LD pruning), ordered by variant positions

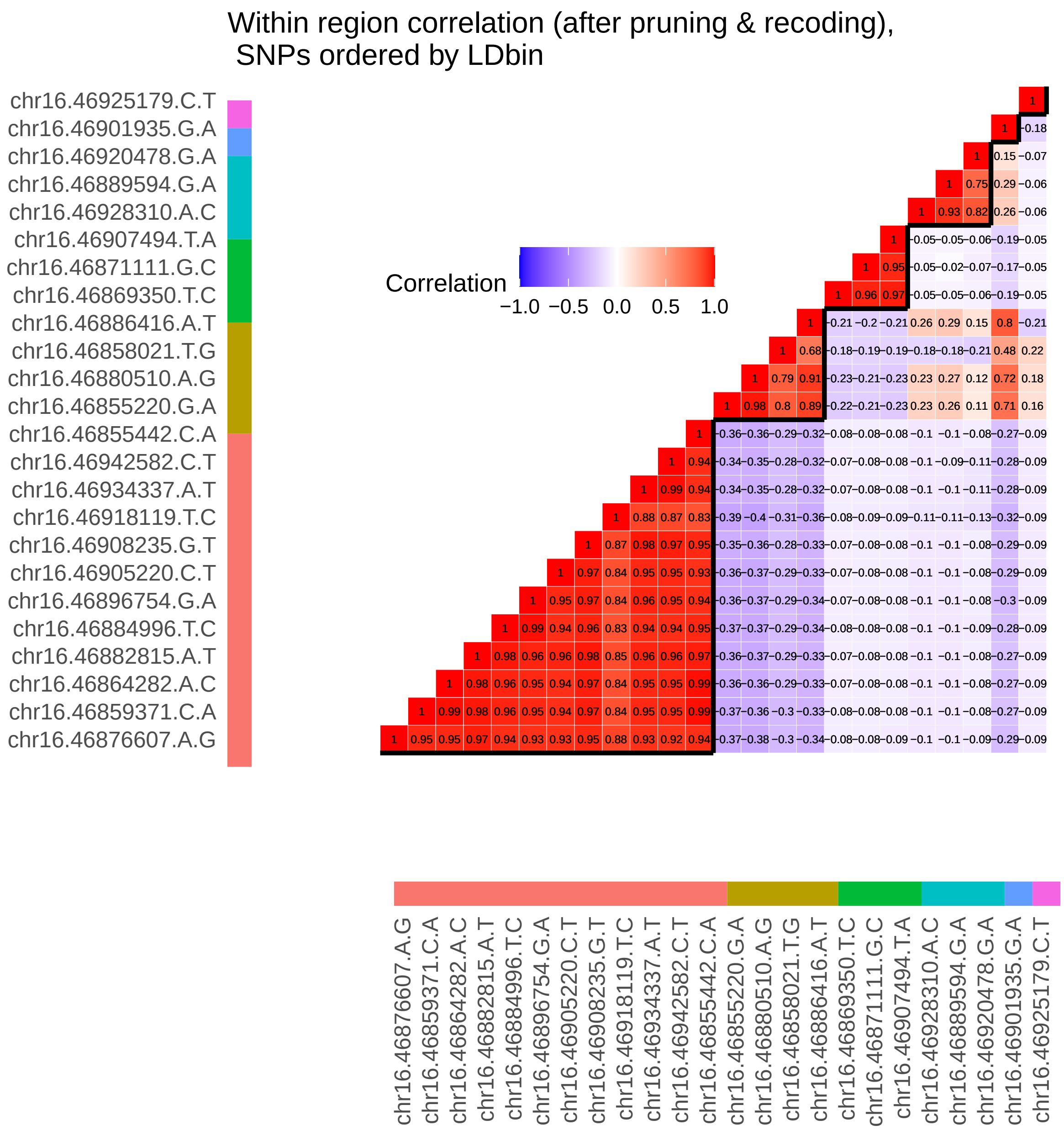

Figure 3: Example 2 of LD heatmap plot for region 8 (after LD pruning), ordered by LD bins

SNP LD bin positions Visualization of the variant positions (X axis) along the LD bins (Y axis) for the variants kept after LD pruning (and in grey, removed by LD pruning). Plots are saved as pdf files in the local directory.

```
MLCbinsnpPlot(rscanout = results, chr_=16, region_ =8 )
```

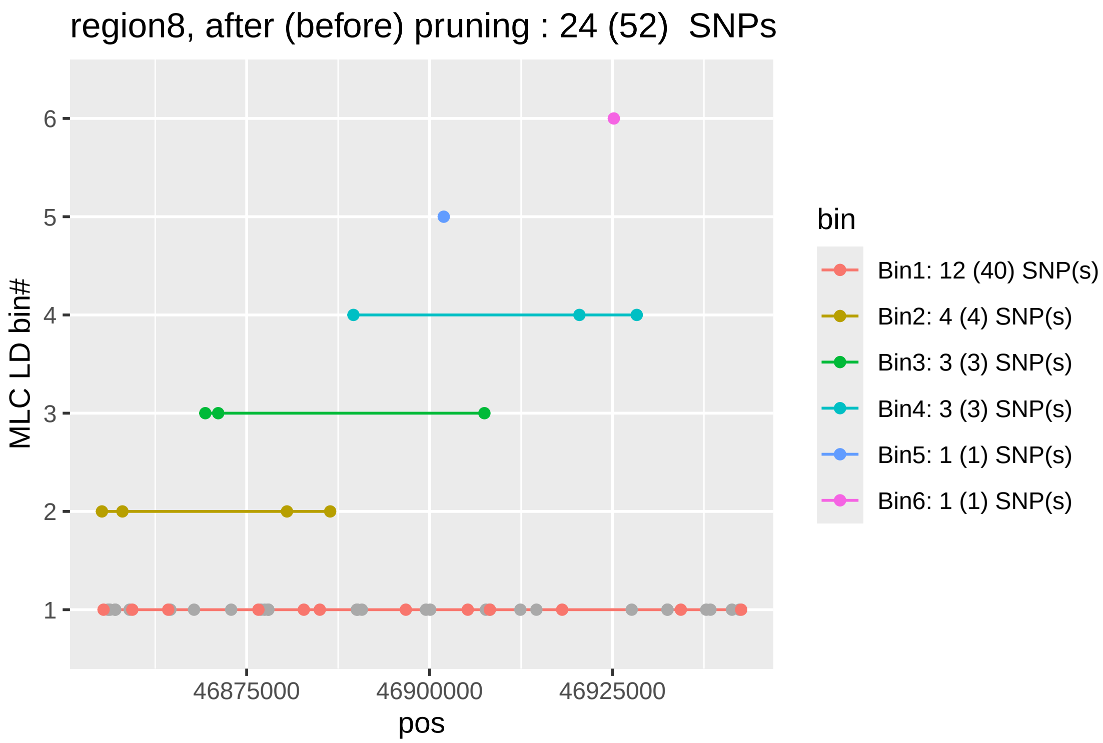

Figure 4: variant positions (X axis) along LD bins (Y axis) in region 8

Citation

RegionScan : A comprehensive R package for region-level genome-wide association testing with integration and visualization of multiple-variant and single-variant hypothesis testing. Brossard M, Roshandel D, Luo K, Yavartanoo F, Paterson AD, Yoo YJ, Bull SB. https://www.biorxiv.org/content/10.1101/2024.03.04.582374v1.

License

This package is released under the GNU General Public License (GPL) v3.0.

ANNEX

Table 1. List of *Main* and *Utility* Functions in RegionScan

Functions	Short description
regscan	Main function for region processing, single-SNP and region-level analysis.
recodeVCF	Extracts information from VCF file for each region, recode variants (including multiallelic variants) and returns genotype and info files used by *regscan.*
MiamiPlot	Produces a Miami plot genome-wide (or in a subset of regions) for any specified pair of tests; can also be used to visualize potential colocalization between region-level tests obtained for two independent studies.
qqregscanPlot	Creates a Quantile-Quantile plot for a specified region-level test and calculates genomic inflation factor.
LocusPlot	Plots region-level test results for a specified set of contiguous regions.
MLCbinsnpPlot	Visualization of genomic positions of the variants assigned to within-region LD bins.

Table 2. List of the main arguments of *regscan*

Arguments	Definition	Default value
REGIONinfo	dataframe including region positions. This dataframe must include the four following columns: "chr", "region", "start.bp", "end.bp". This file can include LD block regions positions as generated using for example, by the "BigLD" function from the *gpart* R package or any type of genes/regions positions.	Required
phenocov	dataframe including the covariates (if applicable) and the phenotype (s) in columns. Individuals (in rows) must be in the same order as in the *data* input.	
covlist	list of covariates to account for in single-SNP and multiple-SNP regression models, including for example, ancestry PCs and/or non-genetic covariates (must be included in *data* or *phenocov* if applicable)	NULL
covout	option to output the covariate coefficients, standard errors and their *P*-values from multi-SNP region-level regression models (used to construct MLC, LC and Generalized Wald tests)	FALSE
pheno	name of the phenotype column in *phenocov* to consider for the analysis	Required
pheno_type	pheno_type="C" if phenotype is **c**ontinuous,<br>or pheno_type="D" if phenotype is **d**ichotomous	Required
geno_type	Format of the genotypes: geno_type="D" if genotypes are in allele dosage format , or genotype format (geno_type="G")	Required

	– argument required for SKAT/SKATO tests	
***If data/info data frames are specified* (in this case, recodeVCF is not used)**		
data	dataframe that includes the genotypes (in columns) and individuals (in rows)	Required if *vcfname* is left empty
SNPinfo	dataframe that includes the information for each variant (in rows), must include the following columns:<br>"chr", "pos", "variant", "ref", "alt", “multialSNP”, "multialSNP.rec","freq.alt"<br>"multialSNP": binary variable=0, if the SNP is bi-allelic (two alleles), or 1 if more than two alleles<br>"multialSNP.rec": indicates if the multi-allelic SNP has been recoded b recodeVCF (such that baseline allele = major allele)<br>“freq.alt": frequency of the alternate allele (minor allele)<br>This input can be generated using the auxiliary function *recodeVCF.*	Required if *vcfname* is left empty
Used only by the auxiliary function recodeVCF if data and SNPinfo are not specified		
vcfname	name of the VCF file (if required)	NULL
qcmachr2	threshold used to filter out SNPs with low Mach R2 imputation quality score	NULL
qcinput	dataframe that includes at least 2 columns: variant & info_score (for filtering based on imputation quality score)	NULL
info_score	threshold used to filter out SNPs with low Info imputation quality score – If this option is specified, “qcinput” must be specified, and “info_score” must be a column in “qcinput”	NULL
Options to include multiallelic SNPs		
multiallelic	If FALSE: extract & process only the biallelic SNPs,<br>If TRUE: include multiallelic SNPs in addition to the biallelic SNPs.	FALSE
multial_nmaxalleles	For extraction of SNPs with less than multial_nmaxalleles alleles (e.g. 2 to extract bi-allelic SNPs (default),<br>3 to extract bi-allelic and tri-allelic SNPs, etc).	2
Options for pruning/filtering of the SNPs in each region		
mafcut	threshold to filter out the SNPs with MAF ≤ *mafcut*	0.05
LDpruning	option to prune out the SNPs within each region based on the absolute value of the region-level genotypes correlation matrix; recommended to reduce the multi-collinearity issues in the multi-SNP region-level regression models (see **section 2.1.2**).	TRUE
rcut	threshold to prune out the correlated SNPs (works only if LDpruning=TRUE)	0.99
Other options		
singleSNPall	Produces an additional output including the single-SNP results (and LD-bin level information) for all the SNPs before LD pruning/alias identification.<br>– can increase computational time.	FALSE

firthreg	If firthreg ="TRUE", use a Jeffreys-prior penalized-likelihood regression implemented in the R package **brlmgm2**(Kosmidis *et al.*, 2020; Kosmidis and Firth, 2021) instead of the default maximum-likelihood logistic regression. This option is recommended for unbalanced case-control data / small minor allele count.	FALSE
parallel	By default, process & run region-level analysis sequentially; if parallel ="TRUE" proceed in parallel.	FALSE
regionlist	list of the region names from REGIONinfo input to be analyzed for the analysis of a subset of regions.	NULL
alltests	By default, output region-level tests: Wald, MLCB, PC80, SKAT, SKATO, LCB, GATES, SimpleM; If alltests="TRUE" reports additional region-level tests: MLCZ, LCZ	FALSE
edgecut	parameter for clustering of the SNPs in LD bins (based on SNP correlation) using the CLQ algorithm applied before MLC test.	0.5
tol	tolerance parameter to deal with convergence issues in regression models.	1e-16
MLCheatmap	For each region, produces four heatmap plots of the region-level SNP correlation matrix, with SNPs ordered by positions & by LD bin, before and after LD pruning (if applicable) as illustrated in **Fig S2.** We recommend using this option for investigation of a subset of regions of interest specified with the option regionlist.	FALSE
SKAT_kernel	type of kernel used for SKAT and SKATO tests. There are 6 types of pre-specified kernels: "linear", "linear.weighted", "IBS", "IBS.weighted", "quadratic" and "2wayIX". See "kernel" argument in SKAT function in for details https://cran.r-project.org/web/packages/SKAT/SKAT.pdf.	linear.kernel
SKAT_weights	a numeric vector of weights for the weighted kernels. When it is NULL, the beta weight with the "SKAT_weights_beta" parameter is used.	NULL
SKAT_weights_beta	a numeric vector of parameters for the beta weights for the weighted kernels. If you want to use your own weights, please use the "weights" parameter. It will be ignored if "weights" parameter is not null.	c(1,25)

Table 3. Region-level output

Column name	Description
chr	chromosome # (as provided in *REGIONinfo* input)
region	region # or name (as provided in *REGIONinfo* input)
start.bp	start region position in bp (as provided in *REGIONinfo* input)
end.bp	end region position in bp (as provided in *REGIONinfo* input)
NSNPs	NSNPs in region (before pruning on LD/perfect linear dependency)
NSNPs.kept	NSNPs analyzed in region (after pruning on LD/perfect linear dependency)
max.VIF	Maximum VIF value among the NSNPs.kept
Wald	Generalized Wald statistic
Wald.df	degree of freedom of the Generalized Wald statistic (= NSNPs.kept)
MLCB	MLCB test statistic
MLCB.df	degree of freedom of the MLCB test (= # of LD bins in the region)
MLCB.p	*P*-value for the MLCB test
LCB	LCB test statistic
LCB.df	degree of freedom of the LCB test (=1 for all the regions)
LCB.p	*P*-value for the LCB test
PC80	PC80 test statistic
PC80.df	degree of freedom of the PC80 test
PC80.p	*P*-value for the PC80 test
SKAT	SKAT statistic
SKAT.pDavies	*P*-values from SKAT computed using Davies's method
SKAT.pLiu	*P*-values from SKAT computed using Liu's method
SKATO.p	SKATO test statistic
GATES.df	degree of freedom for the GATES test
GATES.p	*P*-value for the GATES test
SimpleM.p	*P*-value for the simpleM test
single_Wald.p	Minimum *P*-value from single-SNP analysis among the NSNPs.kept
**If alltests=TRUE**	
MLCZ	MLC statistic based on Z-scores from multi-SNP region-level regression model (rather than on SNP effects as used for MLCB)
MLCZ.p	*P*-value for MLCZ test statistic from multi-SNP region-level regression model (rather than on SNP effects as used for MLCB)
LCZ	LC statistic based on Z-scores (rather than SNP effects) from multi-SNP region-level regression model (rather than on SNP effects as used for MLCB)
LCZ.p	*P*-value for LCZ test statistic

Table 4. Bin-level output (specific to the MLC test)

Column name	Description
chr	chromosome # (as provided in input *REGIONinfo)*
region	region # or name (as provided in input *REGIONinfo)*
start.bp	start region position in bp (as provided in input *REGIONinfo)*
end.bp	end region position in bp (as provided in input *REGIONinfo)*
bin	LDbin # assigned within each region (bin #1 corresponds to the bin with largest bin.size)
bin.size	# of SNPs in LD bin
bin.size.keptSNPs	# of SNPs in LD bin (after pruning on MAF & LD (if applicable),<br>and complete linear dependency)
deltabinB	Bin-level test statistic
deltabinB.p	*P*-value for 1 df Wald test of deltabinB
**If alltests=TRUE**	
deltabinZ	Same as deltabinB but using Z statistics instead of Betas
deltabinZ.p	Same as deltabinB but using Z statistics instead of Betas

Table 5. Variant-level output

Column name	Description
chr	chromosome # (as provided in *REGIONinfo* input)
region	region # or name (as provided in *REGIONinfo* input)
start.bp	start region position in bp (as provided in *REGIONinfo* input)
end.bp	end region position in bp (as provided in *REGIONinfo* input)
variant	variant name (as provided in *SNPinfo* input)
pos	SNP position (as provided in *SNPinfo* input)
multiallelicSNP	indicator variable to flag multiallelic SNPs (as provided in *SNPinfo* input)
ref	Reference allele (as specified in *SNPinfo* input)
alt	Alternate allele (as specified in *SNPinfo* input)
maf	Minor allele frequency
LDbin	LDbin # assigned within each region (numbered by decreasing # of SNPs)
LDbin.size	# of SNPs analyzed in each LDbin (kept after pruning on MAF & LD)
MLC.flip	Flag the SNPs recoded for MLC/LCbin tests
LC.flip	Flag the SNPs recoded for the LCbin tests
sg.beta	SNP effect estimate from single-SNP regression models
sg.pval	*P*-value for 1 *df* Wald test of SNP effect from single-SNP regression models
VIF	Variance inflation factor (VIF) values based on *all SNPs* analyzed in each region
glm.beta	SNP effect estimate from the region-level multi-SNP regression model
glm.pval	*P*-value for 1 *df* Wald test of SNP effect the region-level multi-SNP regression model

Table 6. List of SNPs excluded from the region-level tests and reasons for exclusion

Column name	Description
chr	Chromosome # (as provided in input *REGIONinfo)*
region	region # (as provided in input *REGIONinfo)*
start.bp	end position in bp (as provided in input *REGIONinfo)*
end.bp	start position in bp (as provided in input *REGIONinfo)*
variant	variant name (as provided in input *REGIONinfo)*
multiallelicSNP	indicator variable to flag multiallelic SNPs (as provided in input *REGIONinfo)*
pos	SNP position (as provided in input *SNPInfo)*
MAF	Minor allele frequency
reason	Reason of exclusion:<br>"mafcut" – MAF < *mafcut* (if applicable)<br>"rcut" – high correlation (if applicable)<br>"alias" – complete linear dependency<br>"multial" – multiallelic SNP (if applicable)

Table 7. Optional output produced if singleSNPall=TRUE. This output includes single-SNP results and bin-level information for all SNPs in regions before LD pruning

Column name	Description
chr	Chromosome # (as provided in input *REGIONinfo)*
region	region # (as provided in input *REGIONinfo)*
start.bp	end position in bp (as provided in input *REGIONinfo)*
end.bp	start position in bp (as provided in input *REGIONinfo)*
variant	variant name (as provided in input *SNPinfo*)
pos	SNP position (as provided in input *SNPinfo*)
multiallelicSNP	indicator variable to flag multiallelic SNPs (as provided in input *SNPinfo*)
major.allele	Major allele (as provided in input *SNPinfo*)
minor.allele	Minor allele (as provided in input *SNPinfo*)
maf	Minor allele frequency
LDbin	Bin # within each region (numbered by decreasing # of SNPs)
sg.beta	SNP effect estimate from single-SNP regression models
sg.pval	*P*-value for 1 *df* Wald test of SNP effect from single-SNP regression models
rmcorr	Indicator variable to flag SNPs excluded because of LD pruning
